# Live imaging of neolymphangiogenesis identifies acute antimetastatic roles of dsRNA mimics

**DOI:** 10.15252/emmm.202012924

**Published:** 2021-11-11

**Authors:** David Olmeda, Daniela Cerezo‐Wallis, Cynthia Mucientes, Tonantzin G Calvo, Estela Cañón, Direna Alonso‐Curbelo, Nuria Ibarz, Javier Muñoz, José L Rodriguez‐Peralto, Pablo Ortiz‐Romero, Sagrario Ortega, María S Soengas

**Affiliations:** ^1^ Melanoma Laboratory Molecular Oncology Programme Spanish National Cancer Research Centre (CNIO) Madrid Spain; ^2^ Proteomics Unit Biotechnology Programme, ProteoRed‐ISCIII Spanish National Cancer Research Centre (CNIO) Madrid Spain; ^3^ Instituto de Investigación i+12 Hospital 12 de Octubre Universidad Complutense Madrid Medical School Madrid Spain; ^4^ Department of Dermatology Hospital 12 de Octubre Universidad Complutense Madrid Medical School Madrid Spain; ^5^ Mouse Genome Editing Core Unit Spanish National Cancer Research Centre (CNIO) Madrid Spain; ^6^ Present address: Spanish National Center for Cardiovascular Research (CNIC) Madrid Spain; ^7^ Present address: Memorial Sloan Kettering Cancer Centre New York NY USA

**Keywords:** dsRNA nanoplexes, GEMM melanoma models, midkine, neolymphangiogenesis, premetastatic niche, Cancer, Skin, Vascular Biology & Angiogenesis

## Abstract

Long‐range communication between tumor cells and the lymphatic vasculature defines competency for metastasis in different cancer types, particularly in melanoma. Nevertheless, the discovery of selective blockers of lymphovascular niches has been compromised by the paucity of experimental systems for whole‐body analyses of tumor progression. Here, we exploit immunocompetent and immunodeficient mouse models for live imaging of Vegfr3‐driven neolymphangiogenesis, as a versatile platform for drug screening *in vivo*. Spatiotemporal analyses of autochthonous melanomas and patient‐derived xenografts identified double‐stranded RNA mimics (dsRNA nanoplexes) as potent inhibitors of neolymphangiogenesis, metastasis, and post‐surgical disease relapse. Mechanistically, dsRNA nanoplexes were found to exert a rapid dual action in tumor cells and in their associated lymphatic vasculature, involving the transcriptional repression of the lymphatic drivers Midkine and Vegfr3, respectively. This suppressive function was mediated by a cell‐autonomous type I interferon signaling and was not shared by FDA‐approved antimelanoma treatments. These results reveal an alternative strategy for targeting the tumor cell‐lymphatic crosstalk and underscore the power of Vegfr3‐lymphoreporters for pharmacological testing in otherwise aggressive cancers.

## Graphical abstract

This study reports the “Lymphoreporter‐MetAlert” mice as a platform for non‐invasive imaging and screening for antimetastatic agents. The focus is on melanoma, but these animals can be used for other tumor types and other pathologies that involve an aberrant activation of the lymphatic vasculature.

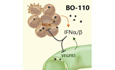

**The paper explained**

**Problem**

Imaging of metastatic niches before and after treatment and pre‐ and post‐surgical removal is a main need in the cancer field. This is particularly the case for malignant melanomas, where lesions barely of millimeters in depth have an intrinsically high metastatic potential. A large fraction of metastatic melanoma patients are or become resistant to current therapies. Therefore, a platform for pharmacological screens *in vivo*, and the identification of novel anticancer agents will have important basic and clinical implications.

**Results**

We have previously generated a “*MetAlert* mice” whereby (pre)metastatic niches can be visualized by whole‐body imaging of the lymphatic vasculature (neolymphangiogenesis). Here, we used these mice as models for preclinical studies of anticancer agents. The *MetAlert* mice revealed an inefficient antilymphangiogenic activity of anticancer agents (inhibitors of BRAF of the immune checkpoint blocker PD‐L1) with limited efficacy in clinical settings. Further screening identified nanoplexes of dsRNA (B0‐110) as potent systemic antilymphangiogenic blockers, with activity observed just after a single administration. This acute effect of BO‐110 was found with a dual repressive action on melanoma cells and the lymphatic vasculature, and we described the underlying mechanism via an IFN‐driven repression of MDK and VEGFR3, respectively.

**Impact**

Live imaging of tumor progression and drug response can facilitate the identification of novel anticancer agents. Here, we discovered an unexpected dual role of the dsRNA mimic BO‐110 in the control of MDK and VEGFR3, which, being so rapid, could aid in assessing drug uptake and therapeutic response in clinical trials. These results set the proof of principle for the *MetAlert* mice as a cost‐effective platform for drug screening in melanoma, as well as in other diseases that involve a pathogenic activation of the lymphatic vasculature.

## Introduction

Clinical intervention in the cancer field has been revolutionized by the identification of (epi)genetic alterations in tumor cells as the basis for rational drug design (Van Allen *et al*, 2014). Prime example of this success is malignant melanoma, where BRAF mutations have led to the generation of effective inhibitors (Robert *et al*, 2019). Unfortunately, these targeted therapies are characteristically transient due to a plethora of mechanisms of resistance (Luebker & Koepsell, 2019; Rossi *et al*, 2019). Immune checkpoint blockers (e.g., anti‐PD1, anti‐PD‐L1, or anti‐CTLA4) are providing unprecedented response rates, particularly in combination with targeted therapies (Herrscher & Robert, 2020). Nevertheless, toxicities can be limiting, and median progression‐free survival remains below 3 years (Herrscher & Robert, 2020). Therefore, the field is actively seeking for more effective agents to treat and prevent metastatic disease (Atkins *et al*, 2021). The expansion of the tumoral lymphatic vasculature (neolymphangiogenesis) is an attractive target for drug development, as this process is one of the earliest events in the dissemination of a variety of aggressive neoplasms (Achen *et al*, 2005; Stacker *et al*, 2014). Moreover, active mechanisms of crosstalk can be established between lymphatic endothelial cells and cancer cells that ultimately create tumor‐permissive lymphovascular niches (Ma *et al*, 2018; Farnsworth *et al*, 2019). A variety of antibodies and small molecules have been developed to trap lymphangiogenic ligands (i.e., VEGFC/D) and/or block the interaction with and subsequent activation of their receptors (VEGFR family members) (Jain *et al*, 2006; Stacker & Achen, 2008; Zheng *et al*, 2014; Maisel *et al*, 2017). Still, none of these antilymphangiogenic treatments has been approved for clinical use, although they are being actively pursued in combination with targeted and immune‐based therapies (Yamakawa *et al*, 2018). Compounds designed for a dual impact on the tumor cells and their associated pathogenic lymphatic vasculature have not yet been described.

A main limitation for the pharmacological assessment of antilymphangiogenic compounds has been the lack of animal models to define when and where lymphovascular niches are activated *in vivo* at distal premetastatic sites (Atkins *et al*, 2021; Patton *et al*, 2021). This has complicated longitudinal analyses of drug response, particularly after surgical removal of primary tumors, which may recapitulate adjuvant treatments that are under active clinical testing (Herrscher & Robert, 2020). In melanoma, a variety of genetically engineered mouse models (GEMMs) have been reported to recapitulate main genetic alterations in this disease, including, but not limited to, the activation of the *Braf* oncogene and the loss of the *Pten* tumor suppressor (Dankort *et al*, 2009; Dhomen *et al*, 2010). Crosses of these animals to various strains that allow for lineage tracing have been highly informative, for example, to define the cell of origin of melanomas (Kohler *et al*, 2017; Moon *et al*, 2017; Soengas & Patton, 2017; Sun *et al*, 2019). Nevertheless, these models do not have the sensitivity for the visualization of micrometastases *in vivo*.

Among lymphatic biomarkers, VEGFR3 represents an attractive candidate for drug screening using imaging techniques. VEGFR3 is highly expressed in lymphatic endothelial cells during development, but becomes downregulated in the adult, being maintained at low levels unless induced by pathological situations such as cancer (Petrova *et al*, 2008; Martinez‐Corral *et al*, 2012). We have exploited this inducibility of VEGFR3 to develop melanoma “lymphoreporter” mice (Olmeda *et al*, 2017). These animals are based on a *knock‐in* strategy whereby an EGFP‐Luciferase cassette is coupled to the endogenous expression of *Flt4 /Vegfr3* (Martinez‐Corral *et al*, 2012). In particular, EGFP was useful to visualize the lymphatic vasculature in the embryo, while luciferase imaging allowed for longitudinal analyses of systemic tumor‐associated neolymphangiogenesis in adult animals (Martinez‐Corral *et al*, 2012). Moreover, spatiotemporal imaging of luciferase in these animals (herein referred to Vegfr3^Luc^ for simplicity) revealed long‐range‐acting mechanisms of neolymphangiogenesis induced already at very early stages of tumor development. Specifically, Vegfr3‐coupled luciferase imaging could be detected in sentinel lymph nodes and multiple visceral sites preceding melanoma metastasis; therefore, these mice were coined as *MetAlert* (Olmeda *et al*, 2017). These *MetAlert* mice, together with loss‐ and gain‐of‐function studies in melanoma cell lines and histopathological studies in human clinical biopsies, ultimately identified the growth factor Midkine (MDK) as a key driver of neolymphangiogenesis and metastasis (Olmeda *et al*, 2017). MDK is expressed in a variety of tumor types (Jono & Ando, 2010; Sakamoto & Kadomatsu, 2012; Sorrelle *et al*, 2017), but it had not been pharmacologically targeted to prevent lymphovascular premetastatic niche activation (Olmeda *et al*, 2017; Sorrelle *et al*, 2017). Therefore, these results highlighted the MetAlert *Vegfr3^Luc^*‐lymphoreporters as a cost‐effective platform for gene discovery (Hoshino & Lyden, 2017; Karaman & Alitalo, 2017; Perez‐Guijarro & Merlino, 2017; Watch, 2017). Here, we define the potential of these mice for *in vivo* testing of anticancer agents. We characterized patient‐derived xenografts and tumors induced by human melanoma cell transplants. In parallel, we used immunocompetent *Vegfr3^Luc^* mice for the assessment of autochthonous melanomas driven by oncogenic *Braf* ^V600E^ and *Pten* loss. Drug‐induced responses were analyzed in two scenarios that recapitulate main clinical needs: (i) established melanomas and (ii) progressive disease after surgical excision of primary lesions. This strategy identified a distinctive therapeutic action of dsRNA‐based nanoparticles in blocking metastasis and preventing tumor relapse. Mechanistically, we found these dsRNA polyplexes to act by dual transcriptional inhibition of *MDK* and *VEGFR3* in both tumor cells and their associated activated lymphatic vasculature, respectively. While this study focused on melanoma, our results underscore the therapeutic potential of targeting the tumor–lymphatic crosstalk in other cancer types and support the *Vegfr3^Luc^* mice as a versatile platform for pharmacological screening of antimetastatic agents.

## Results

### Identification of a potent antilymphangiogenic activity of dsRNA mimics in MetAlert‐lymphoreporter mice

**Figure 1:**
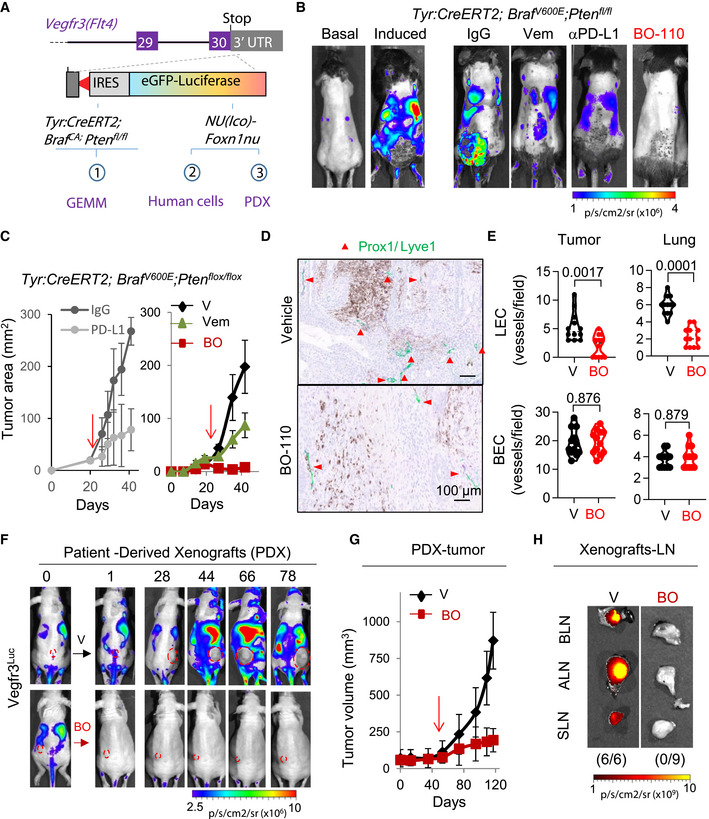
Identification of antilymphangiogenic compounds in *Vegfr3^Luc^* genetically engineered mouse models (GEMMs) Schematic representation of the *Vegfr3^Luc^*‐GEMM (*MetAlert*) mice to assess melanomas driven by melanocytic‐specific induction of oncogenic *Braf^V600E^* in a *Pten*‐deficient background (1), as well as to monitor xenografts of human cells (2) and patient‐derived specimens (PDX, 3).Luciferase‐based imaging of drug response in *Vegfr3^Luc^;Tyr:CreERT2;Braf^V600E^; Pten^flox^* ^/^ *^flox^* mice. Panels labeled as "basal" and "induced" correspond to the bioluminescence of animals prior and 5 weeks after administration of 4OH‐tamoxifen (5 mM, topical administration, 3 consecutive days) for the induction of melanomas. Right panels: Treatment with anti‐PD‐L1 antibody (αPD‐L1; clone 10F.9G2, 3 weeks) or the corresponding control IgG (200 µg/dose, twice per week, 3 weeks); vemurafenib (Vem, 50 mg/kg, oral once per day, 3 weeks), or BO‐110 (BO, 0.8 mg/kg, twice per week, 3 weeks). Scale: p/s/cm2/sr (×10^6^).Growth curves of *Vegfr3^Luc^;Tyr:CreERT2;Braf^V600E^; Pten^flox^* ^/^ *^flox^* melanomas treated with αPD‐L1 or IgG (200 µg/dose, 2 doses/week; left panel), or with vemurafenib (Vem, 50 mg/kg, daily dose), BO‐110 (BO, 0.8 mg/kg, 2 doses/week) or vehicle control (V, daily dose) as indicated. Data correspond to the average tumor size ± SD at the indicated time points. Red arrows mark the initiation of treatment (*n* = min 5 mice per condition). Two‐way ANOVA statistics. *P* = 0.0012 (αPD‐L1), *P* = 0.0007 (BO‐110) and *P* = 0.0353 (vemurafenib).Histological visualization of lymphatic vessel density (dual Lyve11 Prox1 staining) in representative sections of tumors of *Vegfr3^Luc^;Tyr::CreERT2;Braf^V600E^;Pten^flox^* ^/^ *^flox^* melanomas in mice treated with vehicle (V) or 4 doses of BO‐110 (BO, 0.8 mg/kg). Double‐positive (Lyve1, Prox1) vessels were pseudocolored to green to ease the visualization. See also images for lung lymphatic vessels in Fig EV1C. Red arrowheads indicate Lyve1‐Prox1‐positive lymphatic vessels.Quantification of lymphatic and blood vessels density in tumors and lungs of *Vegfr3^Luc^; Tyr::CreERT2*;*Braf^V600E^;Pten^flox^* ^/^ *^flox^* melanomas after treatment as indicated in B, C. Data correspond to the quantification of four fields per tumor, performed in biological triplicates. Statistical significance was determined by the Mann–Whitney *t*‐test.Treatment with BO‐110 of human patient‐derived xenografts (PDX) implanted in *Vegfr3^Luc^ nu/nu*. 42 days after implantation (when systemic luciferase was detected), animals were randomized for treatment with vehicle (V) or with 0.8 mg/kg BO‐110 (BO, twice per week), and luciferase emission was acquired at the indicated times. Scale, p/s/cm^2^/sr (×10^6^).Quantification of the inhibitory effect of BO‐110 (BO, 0.8 mg/kg, 2 doses/week I.P. administration, 11 weeks) on the growth of melanoma PDXs. Red arrows mark the initiation of treatment. Shown are mean tumor size in mm^3^ ± SD in biological triplicates. Statistical significance was determined by two‐way ANOVA. *P* = 0.0009.Representative sentinel, axillary, and brachial lymph nodes (SLN, ALN, and BLN, respectively) of mCherry‐SK‐Mel‐147‐driven xenografts in *Vegfr3^Luc^ nu/nu* mice treated with vehicle (V) or four doses of BO‐110 (BO, 0.8 mg/kg) and imaged for mCherry fluorescence to assess metastatic potential as a function of treatment. Numbers in parenthesis correspond to mice with positive metastases in at least one LN (lymph node) with respect to the total animals analyzed per condition. Scale, p/s/cm^2^/sr (×10^8^). See also Fig EV1D and E for images and quantification of lymphatic vessels in lymph nodes. Source data are available online for this figure.

Given the impact of neolymphangiogenesis on the conditioning and colonization of distal visceral sites in melanoma (Olmeda *et al*, 2017), we hypothesized that the "*MetAlert‐lymphoreporter*" mice could serve as a tractable platform for preclinical studies of anticancer agents. To test autochthonous melanomas, immunocompetent *Vegfr3^Luc^* mice were crossed with *Tyr:CreERT2; Braf^V600E^; Pten^flox^* ^/^ *^flox^* (Dankort *et al*, 2009), a melanoma GEMM broadly used in the melanoma field (see a schematic of the reporter construct and the different strains used in this study in Fig 1A). *Vegfr3^Luc^* mice were also crossed into nude (*nu/nu*) mice as previously described (Olmeda *et al*, 2017), to generate hosts for whole‐body imaging of tumors generated by human cell lines or human patient‐derived xenografts (PDX; Fig 1A). To assess clinically relevant immunomodulators and genetically targeted agents, treatments were performed with a standard anti‐PD‐L1 (αPD‐L1) blocking antibody or with the BRAF inhibitor vemurafenib, respectively (Fig 1B). Treatments were first tested in the *Vegfr3^Luc^; Tyr:CreERT2; Braf^V600E^; Pten^flox^* ^/^ *^flox^* animals (herein referred to *Vegfr3^Luc^‐GEMM*) where melanomas were induced by topical administration of 4‐hydroxytamoxifen (5 mM, 3 consecutive days). Once melanomas reached ˜20 mm^2^, αPD‐L1 antibody (10F.9G2, 200 µg/dose, twice per week) or vemurafenib (50 mg/kg, seven doses per week) was administered systemically for 3 weeks as described in the Methods section. As shown in Fig 1B, both agents delayed tumor growth (see also Fig 1C), thus reducing Vegfr3‐Luc emission. However, and as the case for a large set of patients in the clinic (Robert *et al*, 2019; Herrscher & Robert, 2020), this response was incomplete, as reflected by a residual Vegfr3^Luc^ emission at the implantation site and, importantly, at distal lymphovascular niches (Fig 1B). Therefore, the *MetAlert Vegfr3^Luc^‐GEMM* can be used to monitor drug response *in vivo*, but also emphasized the need for additional treatments with more durable efficacy.

**Figure EV1:**
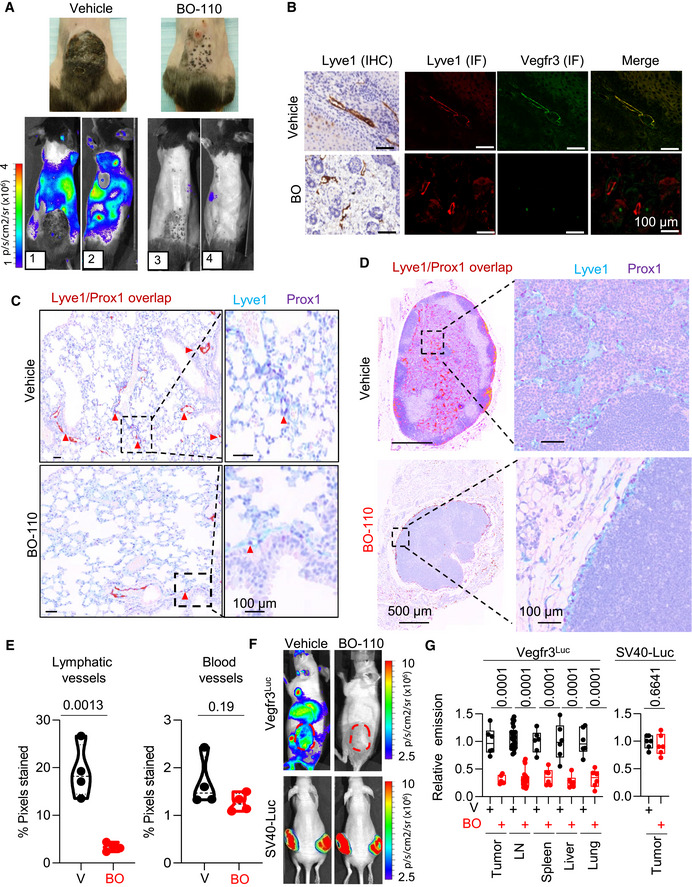
Identification of BO‐110 as an antilymphangiogenic agent in *MetAlert mice*, with controls for selective inhibition of Vegfr3‐Luc signaling Impact of BO‐110 on *Tyr:CreERT2*; *BRAF^V600E^;Pten^flox^* ^/^ *^flox^; Vegfr3^Luc^* mice. Upper panels correspond to optical photographs of animals treated with vehicle or with 6 doses of BO‐110 (0.8 mg/kg, twice a week, 3 weeks), and depilated to ease in the imaging. These same animals are shown in the bottom panels for luciferase emission centering on the tumor (1, 3) or on sentinel LN (2, 4). Scale, p/s/cm^2^/sr (×10^6^).Histological assessment of the impact of BO‐110 (BO) on neolymphangiogenesis in melanomas generated in *Vegfr3^Luc^;Tyr::CreERT2*;*BRAF^V600E^;Pten^fl^* ^/^ *^fl^Pten^flox^* ^/^ *^flox^* mice. Panels correspond to Lyve1 detected by immunohistochemistry (IHC, brown signal) or to dual fluorescence staining for Lyve1 (red) or Vegfr3 (green). Right panel corresponds to merged signal of Lyve1 and Vegfr3 IF.Right panel, histological visualization of lymphatic vessel density by costaining for Lyve1 (blue) and Prox1 (purple) in representative lungs of *Vegfr3^Luc^;Tyr::CreERT2*;*Braf^V600E^;Pten^flox^* ^/^ *^flox^* melanomas treated with vehicle (V) or 4 doses of BO‐110 (BO, 0.8 mg/kg). Left panel, pseudocoloring in red of overlapped staining with anti‐Lyve1 and Prox1 antibodies (cells positive for both markers are highlighted with red arrowheads).Right panel, histological analyses of lymphatic vessel density by Lyve1 and Prox1 dual staining in representative lymph nodes of animals in Fig 2B processed at the endpoint of the experiment (four doses of BO‐110 or vehicle control). Left panel, dual‐positive Lyve1 and Prox1 cells pseudocolored in red.Quantification of lymphatic vessel (left) and blood vessel (right) density in lymph nodes of animals in Fig 2B (SK‐Mel‐147 tumors) processed at the endpoint of the experiment (four doses of BO‐110 or vehicle control). Data correspond to the quantification of four fields per tumor, performed in biological triplicates. Statistical significance was determined by the Mann–Whitney *t*‐test.
*In vivo* imaging of the comparative impact of BO‐110 (0.8 mg/kg) (BO) or vehicle (V) on luciferase emission driven from the Vegfr3^Luc^ MetAlert mice bearing a SK‐Mel‐147 tumor, or from an unrelated promoter (SV40‐Luc) stably expressed in SK‐Mel‐147 melanoma cells.Quantification of luciferase emission 24 h after treatment in the tumor and the indicated organs in animals treated as in (F). *N* = 6 mice per condition. Boxplots show the median, 25^th^ and 75^th^ percentiles, and the maximum and minimum signal. Luciferase signal was normalized to the corresponding vehicle control. Statistical significance was determined by ANOVA. Source data are available online for this figure.

We then questioned whether the *MetAlert* mice could identify compounds with a stronger antitumoral activity and, possibly, new modes of action. Lymphatic endothelial cells can secrete and respond to a variety of immunomodulators (Farnsworth *et al*, 2019), which in the context of aggressive tumors may contribute to immune tolerance (Alitalo & Detmar, 2012; Zheng *et al*, 2014). We considered of interest agonists of pathogen‐activated molecular pattern receptors (PAMPs), particularly dsRNA sensors, as these compounds are being actively pursued for their ability to target tumor cells and shift immunologically "cold" into "hot" tumors (Aznar *et al*, 2019; Hur, 2019). However, whether PAMP inducers impact on lymphovascular niches is unknown. We thus chose to study synthetic (poly)inosinic:polycytidylic acid. This is a mimic of long viral dsRNA, which we had previously demonstrated that can be efficiently delivered to tumor cells when packed into bioavailable nanocomplexes of about 100–150 nm (Besch *et al*, 2009; Tormo *et al*, 2009), herein referred to as BO‐110. BO‐110 is relevant, as a derivative (BO‐112) is currently under clinical testing (Aznar *et al*, 2019), and information in its mode of action may also be of relevance for other dsRNA‐based therapies (Ming Lim *et al*, 2013; Rapoport *et al*, 2014; Salazar *et al*, 2014). First, we tested BO‐110 activity on autochthonous melanomas generated in the Vegfr3^Luc^‐GEMM mice. As shown in Fig 1B, systemic treatment with BO‐110 (0.8 mg/kg, intravenous injections, twice per week, 3 weeks) abrogated Vegfr3^Luc^ emission and tumor growth in a significantly more efficient manner than vemurafenib or αPD‐L1 (Fig 1C; see additional detail in Expanded View Fig 1A). Histological staining for Vegfr3 (Fig EV1B) and for the lymphatic markers Prox1 and Lyve1 confirmed the inhibitory effect of BO‐110 in tumor‐driven neolymphangiogenesis at the cutaneous melanomas (Fig 1D) or at distal organs (see lungs in Fig EV1C) of *Vegfr3^Luc^‐GEMM mice*. Importantly, the inhibitory effect of BO‐110 on lymphatic cells was selective, as we did not observe significant differences in blood endothelial cells neither in tumor sections nor in lungs (see quantifications in Fig 1E).

The *MetAlert* mice were also found highly informative in the immune‐suppressed backgrounds. Thus, we could also visualize an effective inhibitory effect of BO‐110 in Vegfr3^Luc^ emission and tumor growth in the context of patient‐derived xenografts (PDX) that were minimally expanded in culture (Fig 1F and G). Prelabeling well‐characterized aggressive melanoma cell lines (SM‐Mel‐147) with fluorescent agents (mCherry), we could observe that this antitumoral activity of BO‐110 resulted in an effective blockade of metastatic dissemination (see different lymph nodes in Fig 1H), also associated with inhibition of tumor lymphangiogenesis (Fig EV1D), again, without affecting the blood vasculature (Fig EV1E). Therefore, these results validate *Vegfr3^Luc^* reporters as a tractable platform for drug testing *in vivo*, and point to BO‐110 as a distinct blocker of tumor‐induced lymphovascular niches. Importantly, the antitumoral effect of BO‐110 in the immune‐compromised strains used here (which are T‐cell‐deficient) suggested additional roles of this compound beyond reported effects of PAMP inducers on T‐cell function (Aznar *et al*, 2019; Hur, 2019).

### Acute inhibitory action of BO‐110 on prolymphangiogenic factors in lymphatic endothelial cells and melanoma cells

**Figure 2:**
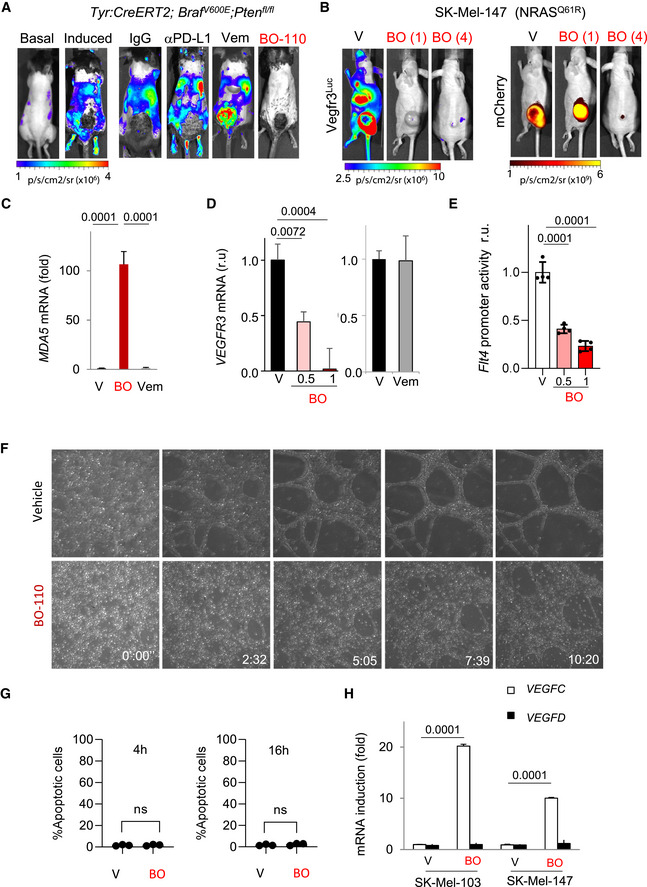
Inhibitory effects of BO‐110 on prolymphangiogenic factors Luciferase‐based imaging of short‐term drug response in *Vegfr3^Luc^;Tyr::CreERT2; Braf^V600E^; Pten^flox^* ^/^ *^flox^* mice. Panels labeled as "basal" and "induced" correspond to the bioluminescence of animals prior and 5 weeks after administration of 4OH‐tamoxifen (5 mM, topical administration, three consecutive days) for the induction of melanomas. Right panels: images of mice that were treated the day before with one dose of the indicated compounds: αPD‐L1 antibody (clone 10F.9G2) or the corresponding control IgG (200 µg/dose); vemurafenib (Vem, 50 mg/kg); or BO‐110 (BO, 0.8 mg/kg). Scale: p/s/cm2/sr (×10^6^).Response of xenografts of mCherry‐labeled SK‐Mel‐147 in *Vegfr3^Luc^* nu/nu lymphoreporter mice treated with one dose (24 h) or 4 doses of BO‐110 (BO, 0.8 mg/kg). Left panels correspond to Vegfr3‐Luciferase (neolymphangiogenesis) and right panels to mCherry fluorescence emission (tumor content). Scale, Vegfr3^Luc^: p/s/cm^2^/sr (×10^6^) and mCherry: p/s/cm^2^/sr (×10^9^).qRT–PCR analysis of relative mRNA levels of *MDA5* 16 h after treatment of HLEC with 0.5 µg/ml BO‐110 (BO), 10 µM vemurafenib (Vem), or vehicle control (V). Data correspond to the mean ± SD of three biological replicates. Statistical significance was determined by the *t*‐test.qRT–PCR analysis of relative mRNA levels of *VEGFR3* 16 h after treatment of HLEC with 0.5 or 1 µg/ml BO‐110 (VO), 10 µM vemurafenib (Vem), or the corresponding vehicle control (V). Data correspond to the mean ± SD of three biological replicates. Statistical significance was determined by ANOVA.Luciferase signal driven by *FLT4 (*VEGFR3*)*‐promoter transduced into HLEC treated with vehicle (v) or BO‐110 (BO) at the indicated doses (µg/ml) as indicated in Methods. Results were normalized to vehicle control. *N* = 4 biological replicates. Error bars correspond to mean ± SD. Statistical significance was determined by ANOVA.Tubulogenic activity of HLECs in the presence of BO‐110. Images correspond to cells plated in Matrigel and imaged at the indicated time points after treatment with 0.5 µg/ml BO‐110. Complete time‐lapse imaging of this process is shown in Movie EV1 (Appendix).Analysis of apoptotic cells at the indicated time points. HLEC cells were treated with vehicle (V) or 0.5 µg/ml BO‐110 (BO) for the indicated time points. Cells were collected and apoptosis was analyzed by flow cytometry as indicated in Methods. Data correspond to the mean ± SD of three experiments. Statistical significance was determined by the *t*‐test.Relative mRNA levels of *VEGFC* and *VEGFD* in the indicated melanoma cell lines 8 h after treatment with vehicle (V) or 0.5 µg/ml BO‐110 (BO), as determined by qRT–PCR. Data correspond to the mean ± SD of three experiments. Statistical significance was determined by the *t*‐test.

In the course of time‐dependent analyses of BO‐110 in the *MetAlert* mice, we observed a reduction of over 80% Vegfr3^Luc^ emission 24 h after a single administration of BO‐110, even before detectable effects on tumor size (see Fig 2A and B). This was the case both for autochthonous GEMMs (Fig 2A) and for xenografts of aggressive melanoma cells (see Fig 2B for mCherry‐labeled SK‐Mel‐147). As a comparison, one dosing of αPD‐L1 antibody or BRAF inhibitor treatment showed virtually no effect on Vegfr3‐Luc emission (Fig 2A).

Being so acute and effective, we looked for potential unspecific effects of BO‐110 that may be linked to luciferase stability, rather than on‐target effects on neolymphangiogenesis. To this end, we tested the effect of BO‐110 on melanoma xenografts where the luciferase signal was driven by an unrelated SV40 promoter, instead of by the endogenous Vegfr3^Luc^. As summarized in Fig EV1F, BO‐110 had no effect on luciferase emission from this SV40 promoter, while parallel studies showed a nearly complete blockade of Vegfr3‐Luc signal (see Fig EV1G for quantifications). We therefore set to question the molecular basis underlying this rapid and effective antilymphangiogenic activity of BO‐110.

We first questioned whether lymphatic endothelial cells (LECs) could directly uptake BO‐110 and respond by repressing this receptor and blunt lymphangiogenesis. HLECs (human lymphatic endothelial cells) were then incubated with BO‐110 for subsequent testing of dsRNA sensors and VEGFR3 expression (note that herein we use the standard Vegfr3 and VEGFR3 nomenclature for mouse and human genes, respectively—this applying also to other genes tested in this study). For dsRNA recognition, we focused on the MDA5 helicase, as we had previously demonstrated that this protein is a key effector of BO‐110 in melanoma cells (Tormo *et al*, 2009). As a reference, we compared BO‐110 with the BRAF inhibitor vemurafenib, because in other systems, this compound activates compensatory effects in the vasculature (Beazley‐Long *et al*, 2015). Doses of these compounds were selected on the basis of previous analyses in melanoma cells (Tormo *et al*, 2009; Bollag *et al*, 2010). This strategy revealed that BO‐110 resulted in a 100‐fold increase of *MDA5* mRNA in LECs, while vemurafenib had virtually no effect (Fig 2C). Moreover, BO‐110, but not vemurafenib, efficiently repressed *VEGFR3* mRNA (see *P*‐values in Fig 2D). We demonstrated this repressive effect of BO‐110 by cloning the *Flt4/VEGFR3* promoter into a luciferase‐based reporter plasmid, and showing a 70% dose‐dependent reduction in emission after treatment (Fig 2E).

Next, we tested the impact of BO‐110 on the functionality of LECs, this assessed by monitoring the formation of tubulogenic structures in three‐dimensional matrices (see Fig 2F for still images and Movie EV1 for a video in real time). Cell viability was analyzed in parallel, to rule out unspecific cytotoxic effects of BO‐110 (Fig 2G). This strategy showed that BO‐110 blocked LEC tube formation (Fig 2F) in conditions with no detectable impact on cell death (Fig 2G).

Being so potent as a blocker of neolymphangiogenesis, we considered the possibility of BO‐110 acting also upstream of VEGFR3. We thus questioned classical activators of VEGFR3 such as VEGFC and VEGFD, which can be secreted by aggressive tumor cells and play key roles in neolymphangiogenesis in cancer (Karaman & Detmar, 2014; Stacker *et al*, 2014). Intriguingly, RNA‐based analyses (RT–PCR) indicated that melanoma‐driven *VEGFD* was not altered by BO‐110 (Fig 2H). Moreover, *VEGFC* was even induced by this compound (see quantifications for two melanoma cell lines in Fig 2H).

**Figure 3:**
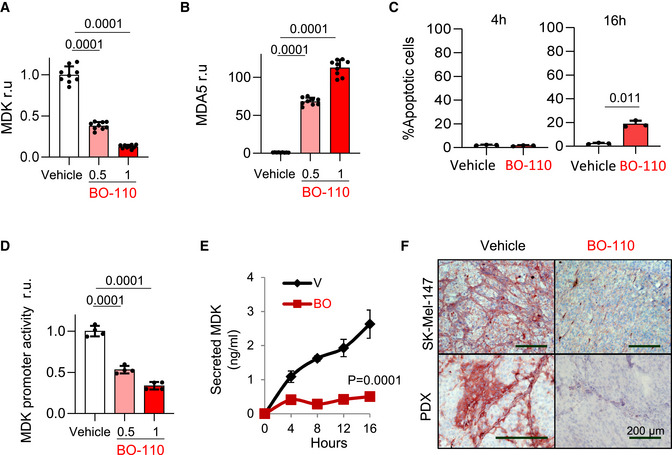
Inhibitory effects of BO‐110 on Midkine Inhibitory effect of the indicated doses of BO‐110 (in µg/ml) or vehicle (V) on *MDK* mRNA expression determined by qRT–PCR in SK‐Mel‐147 (16 h after treatment). Data correspond to average mRNA levels in three experiments with technical replicates normalized to vehicle control ± SD. Statistical significance was determined by ANOVA.qRT–PCR analysis of relative mRNA levels of *MDA5* 16 h after treatment of SK‐Mel‐147 with the indicated doses of BO‐110 (in µg/ml) (BO). Data correspond to the mean ± SD of three experiments with three technical replicates. Statistical significance was determined by ANOVA.Analysis of apoptotic cells at the indicated time points. SK‐Mel‐147 cells were treated with vehicle (V) or 0.5 µg/ml BO‐110 (BO) for the indicated time points. Cells were collected, and apoptosis was analyzed by flow cytometry as indicated in Methods. Data correspond to the mean ± SD of three experiments. Statistical significance was determined by the *t*‐test.Luciferase signal driven by *MDK* promoter transduced into SK‐Mel‐147 cells treated with vehicle (v) or BO‐110 (BO) as indicated in Materials and Methods. Results were normalized to vehicle control. Data correspond to the mean ± SD of four biological replicates. Statistical significance was determined by ANOVA.MDK secretion by ELISA in SK‐Mel‐147 melanoma cells treated with vehicle (V) or 0.5 µg/ml BO‐110 (BO). Data correspond to the mean ± SD of three biological replicates. Statistical significance was determined by two‐way ANOVA.Immunohistochemical analysis of MDK repression (pink staining) in SK‐Mel‐147 xenografts and PDX lesions after treatment with BO‐110 (BO, 0.8 mg/kg, 2 doses/week). Histological staining in tumors extracted from animals treated with vehicle control (V) is included as a reference. Nuclei were counterstained with hematoxylin.

**Figure EV2:**
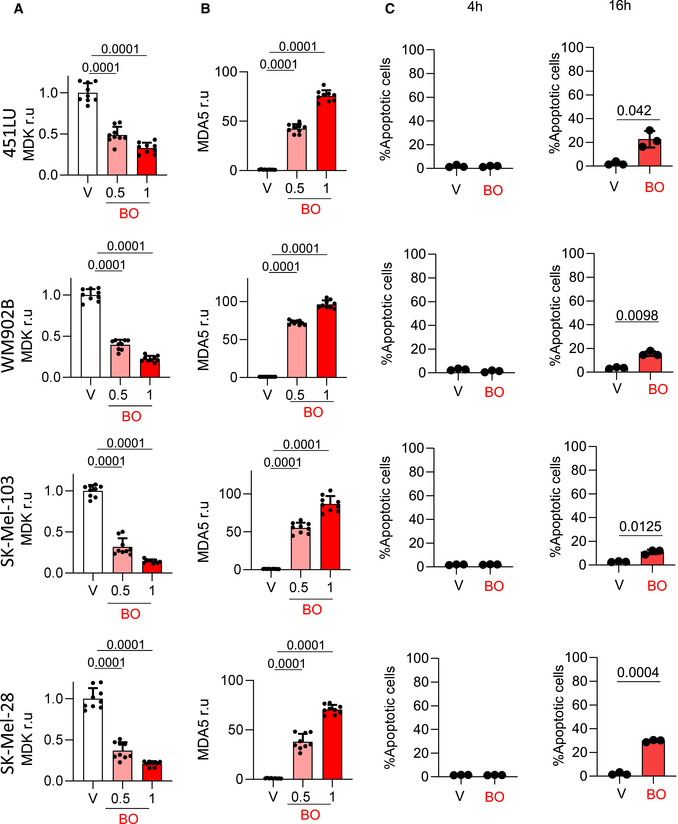
MDA5 induction and Mdk repression by BO‐110 in a panel of melanoma cell lines Inhibitory effect of the indicated doses of BO‐110 (in µg/ml) on *MDK* mRNA expression determined by qRT–PCR in the indicated melanoma cell lines 16 h after treatment. Data correspond to average mRNA levels of three experiments with three technical replicates normalized to vehicle control (V) ± SD. Statistical significance was determined by ANOVA.qRT–PCR analysis of relative mRNA levels of *MDA5* 16 h after treatment of the indicated melanoma cell lines with the 0.5 or 1 µg/ml of BO‐110 (BO) or vehicle (V). Data correspond to the mean ± SD of three experiments with three technical replicates. Statistical significance was determined by ANOVA.Percentage of apoptotic cells (Annexin‐V staining detection by flow cytometry) after treatment of the indicated cell lines with vehicle (V) or 0.5 µg/ml BO‐110 (BO) for the indicated time points. Data correspond to the mean ± SD of three experiments. Statistical significance was determined by the *t*‐test.

We then questioned the growth factor Midkine (MDK) as putative new target of BO‐110. We interrogated MDK as this is a new inducer of neolymphangiogenesis and melanoma metastasis we recently described with prognostic features in human clinical biopsies (Olmeda *et al*, 2017). MDK was relevant as it is overexpressed in a broad variety of cancer types (Sorrelle *et al*, 2017), and no pharmacological agents have been described to block its expression. BO‐110 was found to reduce about 70–60% *MDK* mRNA levels in melanoma cell lines of different genetic backgrounds that recapitulate main protumorigenic mutations characteristic of this disease. Specifically, see Fig 3A for results in SK‐Mel‐147, expressing *NRAS^Q61R^* and Fig EV2A for data in 451LU; WM902B; (*BRAF^V600E^; PTEN^WT^*), SK‐Mel‐28 (*BRAF^V600E^; PTEN^mut^*; P53*^mut^*), or SK‐Mel‐103 (*NRAS^Q61R^*). This inhibitory effect of BO‐110 on *MDK* was parallel to an induction of *MDA5* in all tested cell lines (see the corresponding quantifications and *P*‐values in Figs 3B and EV2B). Importantly, this new activity of BO‐110 on *MDK* was detected at early time points in which tumor cell viability was maintained over 80% (Figs 3C and EV2C). Cloning the *MDK* promoter into a reporter vector confirmed a direct inhibitory effect of BO‐110 on *MDK* transcription (Fig 3D).

This repressive activity of BO‐110 on *MDK* mRNA expression results in a marked, and also early, blockade of MDK secretion by melanoma cells (Fig 3E). Moreover, histological analyses demonstrated that BO‐110 resulted in a potent abrogation of MDK expression *in vivo*, both in xenografts generated by human cell lines and from tumor biopsies (Fig 3F). Together, these data illustrate the versatility of *Vegfr3^Luc^* reporters to discover new mechanisms of action of anticancer agents, including repressors of tumor‐driven MDK.

### IFN‐based inhibition of MDK and VEGFR3 downstream of BO‐110

**Figure EV3:**
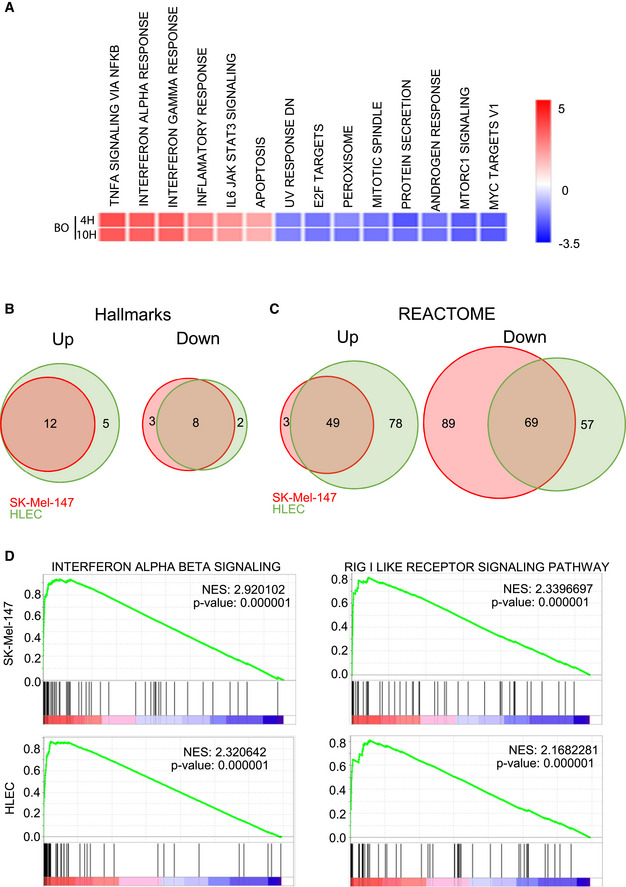
Omic analyses of the BO‐110 mechanism of action Heatmap showing differentially deregulated signaling cascades in SK‐Mel‐147 treated with 1 µg/ml B0‐110 for 4 or 10 h (versus vehicle‐treated controls). Data correspond to mRNA expression profiles analyzed by GSEA using the Hallmark gene sets. The scale indicates the normalized enrichment score (NES).Venn diagrams depicting common and specific Hallmarks gene sets significantly enriched in a GSEA in cell lines SK‐Mel‐147 and HLEC upon BO‐110 treatment (left: upregulated; right: downregulated; FDR < 0.25). See also Datasets EV3 and EV4 for additional detail.Venn diagrams as in B, but estimated for REACTOME gene sets. See also Datasets EV5 and EV6 for the complete gen set list.Enrichment plots for the indicated signaling cascades gene sets upon BO‐110 treatment in SK‐Mel‐147 or in HLEC.

The results above illustrate a dual role of BO‐110 repressing prolymphangiogenic factors both at the tumor and at the LEC level (via MDK and VEGFR3, respectively). These findings have translational implications because although various signaling cascades have been described to upregulate MDK or VEGFR3 (Karaman & Detmar, 2014; Stacker *et al*, 2014; Zheng *et al*, 2014), repressors of these genes are less understood. In fact, to our knowledge, no mechanism has been reported to affect both genes at the mRNA level. We then started with RNA expression analyses in melanoma cells. Profiling SK‐Mel‐147 at early time points after BO‐110 treatment (i.e., 4 and 10 h) revealed marked changes in the transcriptome of these cells (Fig EV3A). These included the downregulation of a series of cell cycle‐associated signaling cascades inhibited by BO‐110, consistent with previous reports by our group and others on antiproliferative activities of dsRNA mimics (Tormo *et al*, 2009; Aznar *et al*, 2019). A variety of proinflammatory signals were also induced, enriched in particular, in interferon (IFN)‐response pathways (Fig E3VA).

**Figure 4:**
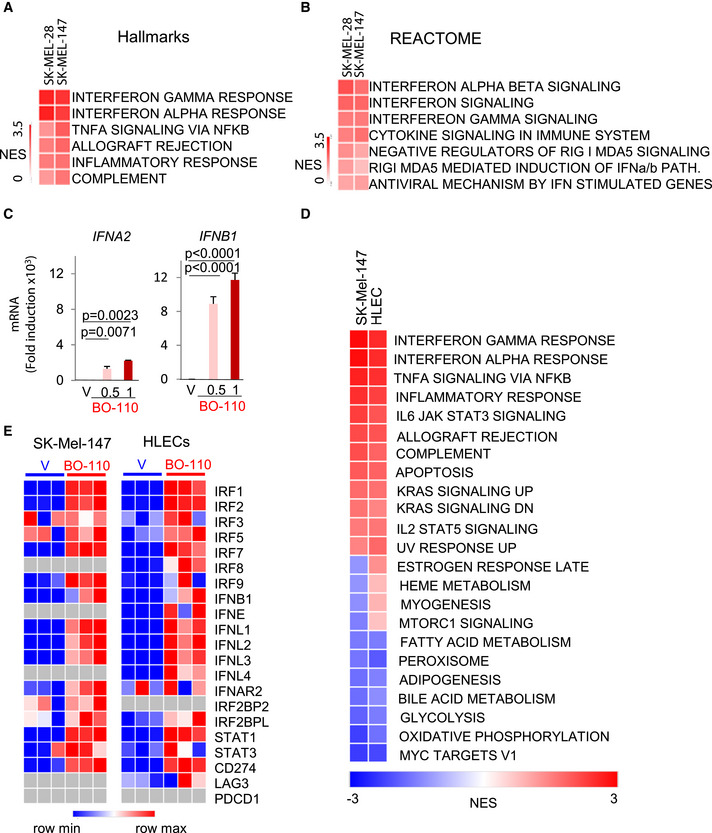
High‐throughput analysis reveals IFN induction as a key component of the BO‐110 mechanism of action Heatmaps summarizing proteomic analyses (iTRAQ) performed in SK‐Mel‐28 and SK‐Mel‐147 after treatment with BO‐110 (1 µg/ml, 10 h). Shown are Hallmark gene sets with NES > 1. See also Datasets EV1 and EV2 for additional information.REACTOME gene set analysis of protein changes in SK‐Mel‐28 and SK‐Mel‐147 cell lines treated with BO‐110 (1 µg/ml, 10 h).Type I IFN mRNA induction (*IFNA2* and *IFNB1*) in SK‐Mel‐147 melanoma cells treated for 16 h with the indicated amounts of BO‐110 (in mg/ml). Data correspond to the mean ± SD of three experiments with three technical replicates normalized to vehicle control. Statistical significance was determined by ANOVA.Heatmap showing differentially deregulated signaling cascades in SK‐Mel‐147 and HLEC treated with 1 µg/ml B0‐110 for 10 h (versus vehicle‐treated controls). Data correspond to mRNA expression profiles analyzed by GSEA using the Hallmark gene sets. The scale indicates the normalized enrichment score (NES). See also Dataset EV3.Heatmap depicting expression changes in interferon‐related genes (GO:0034340) in SK‐Mel‐147 melanoma cells (left panel) and HLEC (right panel) treated with vehicle or 0.5 g/ml of BO‐110 for 10 h. CD274, LAG3 and PDCD1 genes were also included as a reference.

Although dsRNA mimics are well‐known inducers of IFN‐dependent transcriptional programs in cancer cells (Tormo *et al*, 2009; Aznar *et al*, 2019), melanoma cells (as other tumor cell types) express a variety of inhibitory feedback loops (Luke *et al*, 2017). Consequently, mRNA levels may not necessarily translate into significant effects in protein expression. To our knowledge, genome‐wide proteomic analyses have not been performed for dsRNA mimics in cells from tumors or other cell types. Therefore, proteomic analyses were performed by isobaric tag for absolute quantitation (iTRAQ) in SK‐Mel‐147, as well as in SK‐Mel‐28, to test melanoma cells of different genetic backgrounds. Differentially expressed genes were then assessed by GSEA through the Hallmarks (Fig 4A) and REACTOME (Fig 4B) gene set collections. Top ranking upregulated pathways in these analyses were related to type I‐IFN, as well as to dsRNA sensors (MDA5 and RIG1), as summarized in Fig 4A and B (see full lists of up‐ and downregulated gene sets in Datasets EV1 and EV2; FDR < 0.05). RT–PCR validated an efficient induction of *IFNA2* and most notably *IFNB1* by BO‐110 in melanoma cells (see for SK‐Mel‐147 in Fig 4C). Therefore, B0‐110 can overcome intrinsic mechanisms of control of IFN signaling in melanoma cells.

Next, we interrogated the expression profile of hLECs to determine to which extent the response to BO‐110 involved signaling cascades shared (or not) with melanoma cells, as a strategy to identify possible common modulators of MDK and VEGFR3 in the two cell types. GSEA using Hallmarks revealed an > 70% overlap of the pathways induced by BO‐110 in both cell types (Fig EV3B; Datasets EV3 and EV4). These include various inflammatory and IFN‐associated signals (Fig 4D). These data therefore illustrate the ability of BO‐110 to contribute to immune modulation also at the level of the lymphatic vasculature. Analyses through the REACTOME dataset identified a broad spectrum of additional signaling networks deregulated by BO‐110 in the two cell types (Fig EV3C; Datasets EV5 and EV6). Pathways involving mTOR signaling, estrogen response, or protein secretion were differentially regulated in HLEC and melanoma cells (see examples in Figs 4D and EV3 and full gene sets in Dataset EV5). Still, the overlap of pathways found by REACTOME in response to BO‐110 of melanoma cells and HLEC was superior to 50% (Fig EV3C), emphasizing the reactive nature of these two cell types to BO‐110. The top‐5 upregulated gene sets found this approach both HLEC and SK‐Mel‐147 involved a large list of IFN‐related factors that include a variety of cytokines and transcription factors (Fig 4D). Of note, BO‐110 was found to induce immune checkpoint blockers (ICB) such as CD274 (PD‐L1) in both cell types, and LAG3 additionally in HLECs (Fig 4E), which may be of interest for ongoing clinical trials of dsRNA‐based agents in combination with various ICBs (Aznar *et al*, 2019; Kalbasi *et al*, 2020).

**Figure 5:**
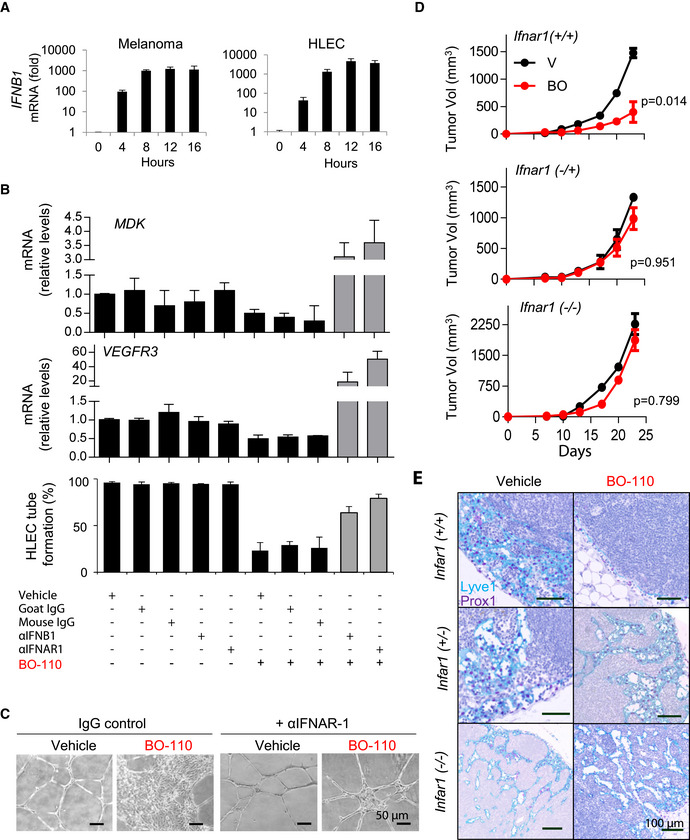
Mechanistic analyses of the repressive activity of BO‐110 on melanoma‐induced neolymphangiogenesis *IFNB1* mRNA induction analyzed by qPCR at the indicated times after BO‐110 treatment (0.5 µg/ml) of SK‐Mel‐147 melanoma cells or HLEC (left and right graphs, respectively). Data correspond to the mean ± SD of three experiments with three technical replicates normalized to vehicle control.Quantification of the impact of BO‐110 as single agent or in the presence of the indicated blocking antibodies for type I interferon (IFNB1 or IFNAR1). Upper graphs show the effect of these agents on *MDK* mRNA levels in SK‐Mel‐147 melanoma cells. Similar treatments were performed on HLEC for the analysis of *VEGFR3* mRNA (middle graphs) and tube formation capacity (lower graphs). Data correspond to the mean ± SD of 3 biological replicates in triplicate.BO‐110‐driven blockade of the tube‐forming capacity of HLEC and rescue with anti‐IFNAR1 blocking antibodies. Images correspond to cells plated in Matrigel and imaged 8 h after treatment with 0.5 µg/ml BO‐110. See also Fig EV4B, for additional results with anti‐IFNAR1 and anti‐IFN‐β blocking antibodies.Growth of B16 melanoma xenografts in siblings of *Ifnar1* ^+/+^, *Ifnar1* ^+/−^, or *Ifnar1* ^−/−^ mice. Treatment started 10 days after tumor cell implantation. BO‐110 was administered at 0.8 mg/kg, every third day for 2 weeks. *N* = 6 mice per condition. Graphs show the mean tumor size ± SD at each time point. Statistical significance was determined by two‐way ANOVA.Histological analyses of lymphatic vessel density by Lyve1 (blue) and Prox1 (purple) in representative lymph nodes of animals in (D) processed at the endpoint of the experiment (four doses of BO‐110 or vehicle control). *N* = 6 mice per experimental condition. See Fig EV4C for a more complete view of these lymph nodes, where dual Prox1‐Lyve1‐positive cells were pseudocolored in red.

**Figure EV4:**
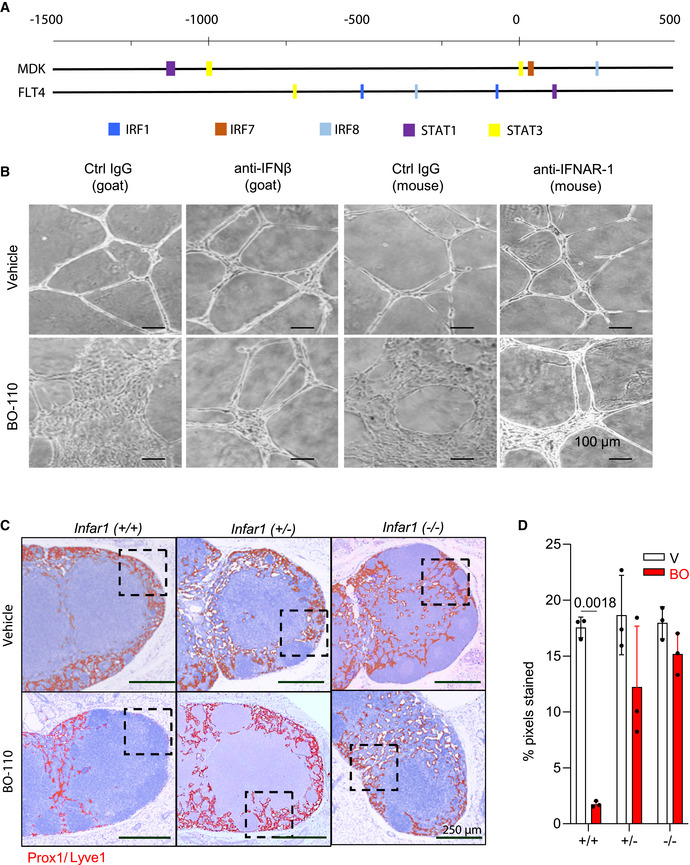
IFN‐dependent inhibition of lymphangiogenesis by BO‐110 Binding sites for experimentally validated IRF‐related transcription factors in the promoters of *MDK* and *FLT4* (*VEGFR3*). Data from Interferome and TRANSFAC (www.genexplain.com).BO‐110‐driven blockade of the tube‐forming capacity of HLEC and rescue with anti‐IFNAR1 blocking antibodies (anti‐IFN‐β and anti‐INFAR1) or corresponding controls. Images correspond to cells plated in Matrigel and imaged 8 h after treatment with 0.5 µg/ml BO‐110.Histological analyses of lymphatic vessel density defined by dual staining for Lyve1 (blue) and Prox1 (purple). To facilitate the visualization, cells positive for both markers were pseudocolored in red. Large magnifications of the doted squared areas can be found in Fig 5E.Quantification of Lyve1/Prox1 staining in lymph nodes of animals in Fig 5D and E, processed at the endpoint of the experiment (4 doses of BO‐110 or vehicle control). Data correspond to the mean ± SD of four biological replicates. Statistical significance was determined by the *t*‐test. Source data are available online for this figure.

Importantly, and emphasizing acute (fast‐acting) roles of IFN responses, we validated an over 1,000‐fold induction of IFNB1 4h after treatment in HLEC with similar kinetics than for melanoma cells (Fig 5A). Therefore, an attractive possibility was that this signaling cascade was responsive for the early coordinated repression of *VEGFR3* and *MDK* mRNA we found, respectively, in these two cell types. Therefore, we checked the INTERFEROME database (Rusinova *et al*, 2013) and the Encyclopedia of DNA Elements (ENCODE) (Davis *et al*, 2018) for genes regulated by IFN‐related transcription factors in other systems. This approach revealed IFN‐response elements in the promoters of *VEGFR3* and *MDK*, with binding sites for transcription factors such as IRF1, IRF7, IRF8, STAT1, or STAT3 (Fig EV4A), were induced by BO‐110 in both cell types (Fig 4E).

To demonstrate the relevance of IFN‐α/β signaling in the inhibitory effect of BO‐110 on *MDK* and *VEGFR3*, we used blocking antibodies against IFN‐β or the IFN‐α/β receptor chain 2 (IFNAR1). As shown in Fig 5B, these two blocking antibodies prevented the reduction of *MDK* expression by BO‐110 in melanoma cells, even increasing its levels. Similarly, anti‐IFN‐β or anti‐IFNAR1 overturned the repression of *VEGFR3* mRNA by BO‐110 in HLEC (Fig 5B), and rescued the tubulogenic activity of these cells, otherwise blocked by BO‐110 (Fig 5C; see additional controls in Fig EV4B). Therefore, these results link BO‐110 to *MDK* and *VEGFR3* mRNA expression via IFN signaling.

A corollary of the data above is that BO‐110 should not be able to blunt tumor‐induced neolymphangiogenesis in an IFN‐defective background. To demonstrate this hypothesis, drug response was analyzed on mice that carry mono‐ or biallelic deletions of the IFN‐α/β receptor 1 (*Ifnar1*) (Muller *et al*, 1994). The cell line used for tumor implants in these studies was B16‐F10, isogenic to the Bl6 background of this *Ifnar1*‐strain. Consistent with the multiple roles of IFN in immune surveillance (Hargadon, 2021), melanoma xenografts grew at a significantly faster rate in the homozygous *Ifnar*‐deficient mice than in the heterozygous or wild‐type littermates (Fig 5D). Interestingly, losing one copy of *Ifnar1* was already sufficient to nearly abrogate the ability of BO‐110 to inhibit melanoma‐driven neolymphangiogenesis (see staining for Lyve1 and Prox1 in Fig 5E, and for more detail in Fig EV4C, and quantifications of this effect in Fig EV4D).

### Spatiotemporal analyses of drug response in the MetAlert mice

**Figure 6:**
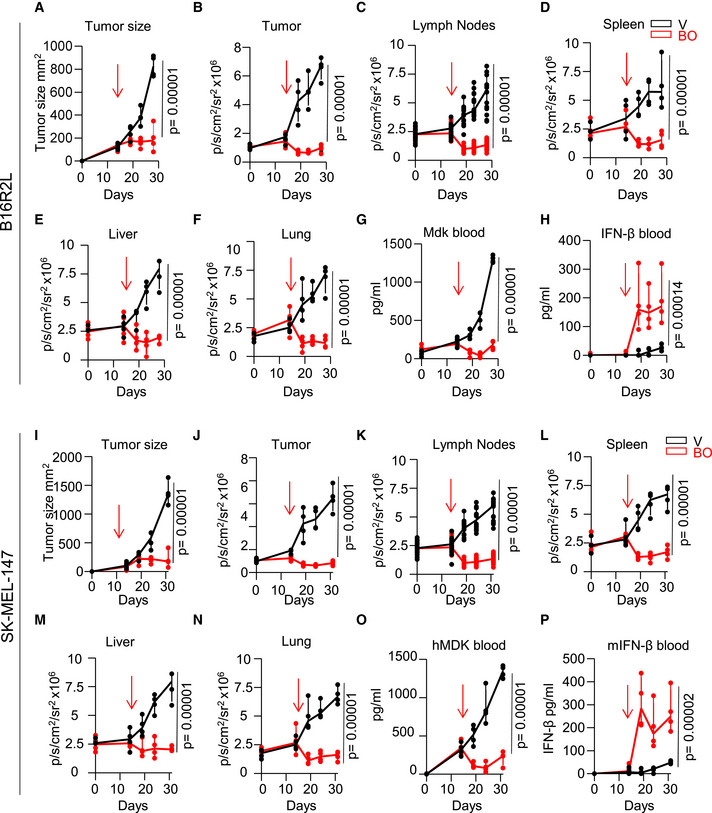
BO‐110 induces a systemic interferon response that inhibits Mdk blood levels and tumor‐induced lymphangiogenesis AImpact of BO‐110 on tumor‐bearing mice implanted with syngeneic B16R2L (5 × 10^5^ cells). When tumors have an average size on 100 mm^3^, mice were randomized into two groups and treated with BO‐110 (BO) or vehicle (V), every second day for 2 weeks. The arrow indicates the start of the treatment. Tumor size was measured with a caliper at the indicated time points.BQuantification of Vegfr3^Luc^ emission in the tumor area in mice in experiment (A).CVegfr3^Luc^ emission in the inguinal and brachial lymph nodes of mice in experiment (A).D–FQuantification of Vegfr3^Luc^ emission in the spleen, liver, and lung of mice in experiment (A), respectively.GELISA analysis of Mdk blood levels in mice in experiment (A).HELISA analysis of mouse Ifn‐β blood levels in mice in experiment (A).IAntitumoral effect of BO‐110 on xenografts of SK‐Mel‐147 (1 × 10^6^ cells) implanted in the back of *Vegfr3^Luc; nu^* ^/^ *^nu^* nude mice. When tumors had an average size on 150 mm^3^, mice were randomized into two groups and treated with BO‐110 (BO, 0.8 mg/kg) or vehicle (V), every second day for 2 weeks. The arrow indicates the start of the treatment. Tumor size was measured with a caliper at the indicated time points, and tumor volume was calculated as indicated in Materials and Methods.JQuantification of Vegfr3^Luc^ emission in the tumor area in mice in experiment (I).K–NQuantification of Vegfr3^Luc^ emission in the lymph nodes, spleen, liver, and lung of mice in experiment (I), respectively.OELISA analysis of Mdk blood levels in mice from (I).PELISA analysis of mouse Ifn‐β blood levels in mice from (I). Data information: For all panels in this figure, *N* = 4 mice per condition. Statistical significance was determined by two‐way ANOVA.

Collectively, the data above illustrate how the *Vegfr3^Luc^ MetAlert* mice can be geared to the discovery of antilymphangiogenic factors, BO‐110 in this case, with novel modes of action in tumor cells and HLEC, with an IFN‐dependent inhibition of *MDK* and *VEGFR3*, respectively. The ability to monitor tumor‐driven luciferase emission at the whole body level adds yet further versatility to these mice, for example, for otherwise quite challenging spatiotemporal pharmacological studies. Specifically, we set to test two clinically relevant aspects of the antitumoral activity of BO‐110: (i) organ‐dependent efficacies (namely, tumor vs lymph node and visceral sites), and (ii) the possibility of exploiting MDK as a biomarker of response in liquid biopsies (blood samples). To this end, we generated subcutaneous xenografts of high‐MDK‐expressing melanoma cells: the murine B16R2L (Fig 6A–H) and the human SK‐Mel‐147 (Fig 6I–P) for analyses of drug response in immune‐competent and T‐cell‐deficient *MetAlert* mice, respectively. Treatment with BO‐110 started when the cutaneous lesions were palpable (around 100 mm^3^; see arrows for all panels in Fig 6) and proceeded for 2 weeks. In the absence of BO‐110, both of these lines are very aggressive as previously reported (Olmeda *et al*, 2017; Cerezo‐Wallis *et al*, 2020). In these conditions, Vegfr3^Luc^ emission correlated with tumor growth at the site of implantation (Fig 6B and J), and was induced systemically in the lymph nodes (Fig 6C and K), spleen (Fig 6D and L), lung (Fig 6F and M), and liver (Fig 6E and N). Both lines responded very efficiently to BO‐110, reducing tumor growth and neolymphangiogenesis at the skin and all the organs tested (Fig 6A–F and I–N). Circulating MDK and IFN‐β were then assessed by ELISA in blood specimens collected at different time points from the control vs BO‐110‐treated groups. This approach revealed that MDK could indeed be detected in blood, reflecting tumor size (Fig 6G and O). Importantly, BO‐110 acutely reduced circulating MDK (Fig 6G and O) with a concomitant induction of IFN‐β also occurring at early time points after treatment (Fig 6H and P). Note that for SK‐Mel‐147, the immunoadsorption assay detects the human form of MDK (i.e., not from the host), thus allowing to monitor tumor‐driven effects of this protein. Therefore, these results support an efficient antitumoral activity of BO‐110 exerted at the whole‐body level, with tumor‐secreted MDK as a biomarker of response (more tightly associated with the melanoma cells than IFN, which can be produced by multiple tumor types).

### Long‐term impact of BO‐110 preventing metastatic relapse after surgery

**Figure EV5:**
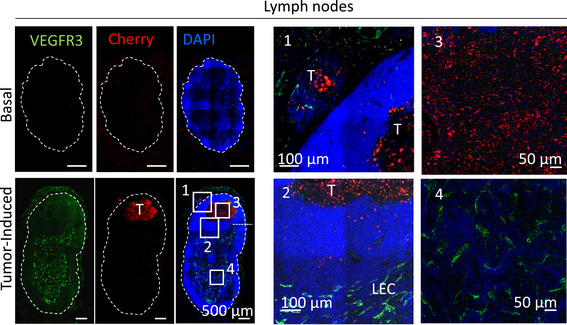
Disseminated tumor cells at lymph node and lung lymphovascular metastatic niches Histological analysis of lymph nodes in basal conditions or in mice implanted s.c with mCherry‐labeled SK‐Mel‐147 performed before tumor excision as in Fig 7A. Vegfr3 and mCherry were detected by immunofluorescence (green and red signaling, respectively). Panels 1–4 correspond to a larger magnification of different areas in these lymph nodes. Note that Vegfr3 concentrates in lymphatic endothelial cells (LECs), with undetectable levels in melanoma cells. T, tumor cells.

One of the main clinical complications of primary melanomas is their potential for metastasis already from seemingly thin lesions. Thus, at the time diagnosis is performed and the cutaneous lesions are removed, tumor cells may be already be disseminated to distal organs (Khoja *et al*, 2015; Scatena *et al*, 2021). A main need in the field is to develop models to monitor this metastatic relapse after surgical excision (Patton *et al*, 2021). Therefore, we questioned whether monitoring neolymphangiogenesis via the *Vegfr3^Luc^* mice could serve as a platform to monitor (and attack) metastatic relapse after surgery. To this end, xenografts of human mCherry‐labeled SK‐Mel‐147 melanoma cells were implanted subcutaneously. Primary lesions were excised when Vegfr3^Luc^ was detectable in a systemic manner, at time points where micrometastases confirmed histopathologically in parallel studies in the lymph nodes (see Fig EV5) mimicking patients with melanomas at stage III that would be considered for adjuvant therapy (Han *et al*, 2021).

**Figure 7:**
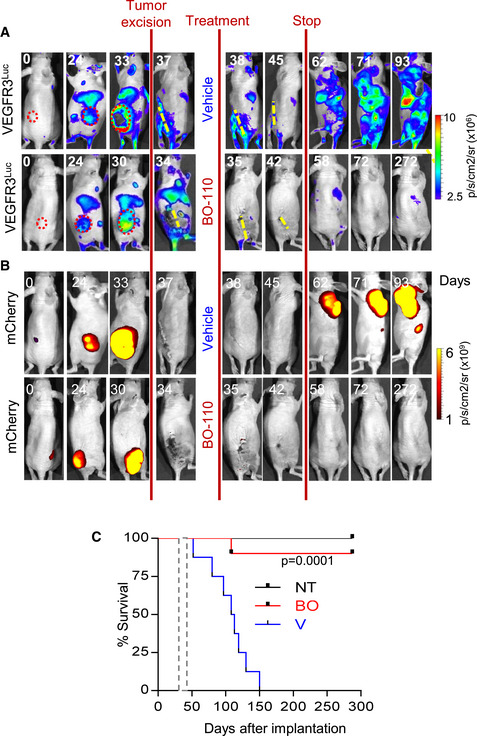
BO‐110 adjuvant treatment prevents metastatic melanoma relapse Efficacy of BO‐110 as an adjuvant (preventing relapse after surgical removal of the primary lesion). Shown are representative examples of *Vegfr3^Luc^* mice implanted with mCherry‐SK‐Mel‐147 and imaged for luciferase emission (prior to and after tumor removal). Animals were left to recover from surgery (4 days) and then treated for 2 weeks (four doses) with 0.8 mg/kg BO‐110 or vehicle control (*n* = 8 for control and *n* = 10 for treatment arm). Scale, p/s/cm^2^/sr ×10^6^.mCherry emission from tumor cells of the animals in (A). Scale, p/s/cm^2^/sr ×10^9^.The Kaplan–Meier survival curves of animals treated as in (A). 8 of 8 animals treated with vehicle (V) control had to be sacrificed for humane reasons 110 days after surgery. 9 of 10 animals in the BO‐110 arm (BO) remained tumor‐free 8 months after stopping treatment. The gray box marks the period of treatment with BO‐110. Statistical significance was determined by the logrank test.

As shown in Fig 7A, surgery resulted in a progressive reduction in systemic Vegfr3^Luc^ emission, consistent with the need for tumor‐driven MDK secretion to activate premetastatic niches (Olmeda *et al*, 2017). Interestingly, Vegfr3‐bioluminescence was regained with time (Fig 7A, right panels), and macrometastases ultimately developed as observed by monitoring tumor cell burden by mCherry fluorescence (Fig 7B). Importantly, 4 doses of BO‐110 (every third day, starting 4 days after surgery) prevented both the re‐acquisition of lymphangiogenesis and the subsequent tumor relapse (Fig 7A). Importantly, the efficacy of BO‐110 was long‐lasting, as 90% of animals remained tumor‐free 8 months after treatment, whereas the average survival for the control group was 3 months (see Kaplan–Meier survival curves in Fig 7C; *P* = 0.0001). Together, these results emphasize the versatility of *Vegfr3^Luc^*‐GEMM reporter mice for non‐invasive studies of melanoma initiation and progression, as well as a tractable platform for pharmacological screening of compounds to prevent and attack tumor metastasis.

## Discussion

Here, we present immunocompetent and immunodeficient *Vegfr3^Luc^* “*MetAlert”* mice as an *in vivo* screening platform for anticancer agents. Specifically, our data support the value of these models as a versatile resource for spatiotemporal analyses of drug response on established tumors, as well as in adjuvant settings that involve surgical excision of cutaneous lesions. The potential for the identification of potent antilymphangiogenic factors is illustrated by the discovery of unanticipated therapeutic activities of the dsRNA mimic BO‐110 (see schematic in Synopsis). We had initially identified BO‐110 as a potent inducer of hyperactivated autophagy in tumor cells (Tormo *et al*, 2009; Alonso‐Curbelo & Soengas, 2010). The Vegfr3^Luc^ reporters have now revealed an acute ability of BO‐110 to blunt lymphangiogenesis at primary and distal sites, in conditions preceding tumor cell death. Guided by these results, we proceeded to a mechanistic study that ultimately uncovered a dual IFN‐dependent repressive role of BO‐110 on tumor cells (inhibiting *MDK* expression and secretion) and on lymphatic endothelial cells (repressing on *Vegfr3* mRNA levels). These functions are distinct from the reported action of lymphangiogenic factors in clinical testing (Stacker *et al*, 2014; Yamakawa *et al*, 2018), and were not recapitulated by FDA‐approved therapies based on BRAF inhibition and checkpoint blockade. Of note, BO‐110 did not promote detectable damaging effects on normal lymphatic vessels. Therefore, the *Vegfr3^Luc^* reporters have uncovered differential effects of dsRNA mimics not only in cancer cells and their associated vasculature, but on pathological versus normal lymphatic vessels as well. This information is timely, because a large range of factors have been found to promote lymphangiogenesis, but endogenous blockers of this process are less characterized (Farnsworth *et al*, 2019).

Perhaps one of the most unexpected results of this study was the finding that one single administration of BO‐110 nearly abrogated Vegfr3^Luc^ emission *in vivo* in melanomas of different genetic backgrounds. This information could potentially be used in clinical trials that are currently testing derivatives of BO‐110 and other formulations of dsRNA (Ming Lim *et al*, 2013; Rapoport *et al*, 2014; Salazar *et al*, 2014; Aznar *et al*, 2019; Hur, 2019). For example, our data provide the proof of concept for using VEGFR3 and/or MDK inhibition (e.g., in needle biopsies of tumors or lymph nodes), as early markers to gauge drug delivery and response to dsRNA mimics in treated patients. In particular, our findings revealing circulating MDK as a biomarker that reflects reduced tumor burden after BO‐110 treatment may prove useful to assess the antitumoral activity of this compound beyond less specific IFN‐associated markers. Importantly, we have recently reported that MDK exerts potent immune‐suppressive roles on macrophages and cytotoxic T cells (Cerezo‐Wallis *et al*, 2020). Therefore, pharmacological blockade of MDK may have the added value of not only interfering with tumor neolymphangiogenesis and metastasis, but also impinging on the immune milieu.

It is tempting to speculate that the dual ability of BO‐110 to target both MDK in cancer cells, and VEGFR3 in lymphatic cells, may represent an advantage with respect to other antilymphangiogenic agents. Thus, various tyrosine kinase inhibitors, VEGFC/D traps or VEGFR3‐VEGFC/D interaction competitors, have been reported to interfere with VEGFR3 function (not its expression), but they exert incomplete responses or are limited by secondary toxicities (Stacker *et al*, 2014; Yamakawa *et al*, 2018). In this context, it would be interesting to further address the multiple cytokines and immunomodulatory agents we found here to be under induced transcriptionally by BO‐110 in both cell types, for combined treatments of BO‐110 and immune checkpoint blockers.

The dual effect of BO‐110 on VEGFR3 and of MDK via IFN signaling raises other practical considerations. For example, BO‐110 and other dsRNA‐based polyplexes are being actively pursued for their ability to (re)activate the innate immune system in aggressive cancers, with a particular interest in cytotoxic CD8^+^ T cells, among others (Akira & Takeda, 2004; Hervas‐Stubbs *et al*, 2011; Aznar *et al*, 2019). Here, we showed that even if T‐cell functions were aberrant in patients (namely, recapitulating the *Foxn1^nu^* nude mice used here), BO‐110 would still be incorporated by tumors and lymphatic endothelial cells, activating IFN and blunting protumorigenic signals in both compartments.

Of note, this study focused on BO‐110 and FDA‐approved BRAF inhibitors and immune checkpoint blockers. However, the ability to monitor whole‐body responses in the *Vegfr3^Luc^* mice could represent a cost‐effective strategy to interrogate new compounds and drug combinations. Thus, these animals reveal when and where neolymphangiogenesis is induced *in vivo* and therefore can be used to set treatment regimens before or after this process is activated (i.e., to mimic preventing, adjuvant, or curative settings in the clinic). Moreover, it is important to consider that VEGFR3 and MDK are deregulated in a variety of tumor types and inflammatory diseases (Jones, 2014; Sorrelle *et al*, 2017; Weckbach *et al*, 2018; Yamakawa *et al*, 2018; Yuan *et al*, 2019). Therefore, data here expand the range of neoplasms where the *Vegfr3^Luc^* mice could be exploited for gene discovery and pharmacological analyses. This could include functional analyses of prolymphangiogenic factors identified in genomic screens that have yet to be validated (Williams *et al*, 2017). In addition, live imaging with the *Vegfr3^Luc^* mice could be used to assess other poorly understood functions of lymphatic endothelial cells in vascular patterning during development and wound healing, or a variety of pathologies that include type 2 diabetes and organ transplant, among others (Alitalo, 2011; Farnsworth *et al*, 2019). Similarly, assessing and importantly inhibiting MDK expression may also be of relevance in the context of proinflammatory roles of this protein in autoimmune and degenerative diseases (Aynacioglu *et al*, 2019; Herradon *et al*, 2019; Weckbach *et al*, 2019). The ability to compare immunocompetent and immunosuppressed backgrounds in the *Vegfr3^Luc^* MetAlert reporter adds yet further physiological relevance to whole‐body imaging of lymphangiogenesis in basal conditions and pathogenic situations.

## Materials and Methods

### Mouse breeding, induction of nevi and melanomas in Vegfr3^Luc^‐GEMM, and drug treatments *in vivo*

The *Vegfr3^Luc^* nu/nu immunodeficient mice and the *Vegfr3^Luc^; Tyr:CreERT2; Braf^V600E^; Pten^flox^* ^/^ *^flox^* animals were generated as described before (Olmeda *et al*, 2017) and maintained in a specific pathogen‐free (SPF) area with a 12‐h light–dark cycle at room temperature. All groups had ad libitum access to food and water throughout the whole study. Only females were used for experimentation to avoid basal Vegfr3‐Luc emission in testis. Specifically, strains used in this study are as follows: *Vegfr3^EGFPLuc^* (*Flt4^tm1.1Sgo^*) (Martinez‐Corral *et al*, 2012); *nu/nu* (*Crl:NU(Ico)Foxn1^nu^*); *Tyr::CreERT2/1Lru* (Yajima *et al*, 2006); *Braf^CA^* (*Braf^tm1Mmcm^*) (Dankort *et al*, 2009); and *Pten^tm2Mak^* (Marino *et al*, 2002). Melanomas in the *Tyr::CreERT2* strains were induced in 14‐week‐old mice by topical treatment with 5 µl of 5 mM 4‐hydroxytamoxifen. BO‐110 (a polyplex of pIC complexed with polyethylene imine) was prepared as described before (Tormo *et al*, 2009). When indicated, 0.8 mg/kg BO‐110 was injected intravenously every 3 days (for a total of 4 administrations, unless indicated otherwise). αPD‐L1 antibody (Bioxcell, Lebanon, NH) was injected IP twice per week (200 µg/dose) unless indicated otherwise. Vemurafenib (Selleck Chemicals, TX) was administered at 50 mg/kg (orally, once a day, during 3 weeks) as previously described (Yang *et al*, 2010). Animals were randomized into the different treatment arms before the start of the treatment. No blinding was performed.

### Non‐invasive imaging of tumor growth and neolymphangiogenesis *in vivo*

Non‐invasive imaging of luciferase in the *Vegfr3^Luc^*‐GEMM was performed using an IVIS‐SPECTRUM imaging system (PerkinElmer, Baesweiler, Germany) essentially as described before (Olmeda *et al*, 2017). Animals were anesthetized with isoflurane and injected intraperitoneally with 150 mg/kg luciferin (PerkinElmer). First, mCherry‐tumor cell images were captured using the appropriate filters. Sequential images of luciferase emission were obtained every minute afterward, and the maximum light emission was determined for each animal as previously described (Martinez‐Corral *et al*, 2012; Olmeda *et al*, 2017). Photons emitted from specific regions were quantified using Living Image software 4.3 (Perkin Helmer). *In vivo* luciferase activity is presented in photons per second per square centimeter per steradian (radiance). All experiments with mice were performed in accordance with protocols approved by the Institutional Ethics Committee of the CNIO and the Instituto de Salud Carlos III.

### Melanoma cells

Melanoma cells (SK‐Mel‐28, SK‐Mel‐147, 451LU, WM902B, B16R2L, and B16‐F10), obtained from (ATCC), were cultured in DMEM (Invitrogen) supplemented with 10% FBS. All cell lines were authenticated using The GenePrint 10 System (Promega, MA). Cells were tested for mycoplasma contamination (Mycoplasma Detection Kit (LT07‐318), Lonza, Basel, Switzerland) regularly and before injection in mice. When indicated, cells were stably infected with mCherry pLV‐puro lentiviral vectors as described before (Tormo *et al*, 2009).

### Melanoma cell xenografts, patient‐derived xenografts, and spontaneous metastasis assays

Xenografts of melanoma cell lines were generated in 14‐week‐old female *Vegfr3^Luc^ nu/nu* mice by subcutaneous implantation of 1 × 10^6^ cells. Tumor growth was recorded by measuring the two orthogonal external diameters using a caliper. Tumor volume was calculated using the formula (a × b^2^ × 0.52). Tumors were excised and processed for histological analysis when they reached 1.5 cm^3^. For spontaneous metastasis assays, SK‐Mel‐147 tumors were grown in the same conditions as described above until they reached a size of 1.2 cm^3^. Surgical excision was then performed under analgesics (buprenorphine 0.05 mg/day), and animals were left to recover for subsequent imaging of tumor growth and luciferase emission at increasing time periods. When indicated, treatments for the prevention of metastatic relapse were initiated 4 days after surgery.

Patient‐derived xenografts (PDX) were generated from biopsies of skin metastases obtained from the Hospital 12 Octubre, Madrid, under their appropriate ethical protocols and provided to the investigators as anonymized lesions. These biopsies were excised into 4‐mm cubes, embedded in Matrigel (BD), and implanted in the back of highly immunodeficient NSG mice (NOD. Cg‐*Prkdc^scid^ Il2rg^tm1Wjl^*/SzJ). Once the tumors reached 1,000 mm^3^, they were excised, processed again in 4‐mm cubes, and reimplanted in the back of 3–6 NSG mice for amplification and subsequent reimplantation in *Vegfr3^Luc^ nu/nu*.

### Histological analyses of gene expression in mouse tumors

Histological analyses of tissue architecture and expression of lymphangiogenic markers were performed on biopsies fixed in formalin and embedded in paraffin. Sections were prepared for hematoxylin‐and‐eosin (H&E) staining. For immunostaining, 3‐µm paraffin sections were deparaffinized and placed in PBS. Slides were incubated with the indicated primary antibodies as described below and developed with Ultravision ONE Detection System Kit (Thermo Scientific, TL‐015‐HAJ) using Permanent Mounting Medium (Prolong, Thermo Scientific). Analyses of lymphatic density were performed in an automated immunostaining platform (Autostainer (AS) Link 48, Dako, Agilent; Discovery XT, Ventana, Roche). For double Prox1/Lyve1 staining, antigen retrieval was first performed with a low pH buffer, and endogenous peroxidase was blocked with 3% peroxide hydrogen. Then, slides were incubated with goat anti‐Prox1 (see provider below, at a 1/2,000 dilution). The slides were subsequently incubated with the corresponding secondary antibody (anti‐goat) conjugated with horseradish peroxidase. The immunohistochemical reaction was developed in purple using 3, 30‐diaminobenzidine tetrahydrochloride (FLEX DAB, Dako, Agilent). Next, for Lyve1 staining, antigen retrieval was performed with CC1 buffer (Ventana Medical Systems, Inc. Santa Clara, CA). Slides were then incubated with the rabbit anti‐Lyve1 (1/250 dilution). After the incubation, slides were incubated with the corresponding secondary antibody (anti‐rabbit) conjugated with horseradish peroxidase. The immunohistochemical reaction was developed in blue using Teal chromogen (Discovery Teal HRP Kit, Ventana Medical Systems). Nuclei were counterstained with Carazzi's hematoxylin. Slides were then dehydrated, cleared and mounted with a permanent mounting medium (Prolong, Thermo Fisher) for microscopic evaluation. For immunofluorescence, tissue sections were deparaffinized, incubated overnight with primary antibodies at 4°C in a humidified chamber, and then rinsed and incubated with fluorescent secondary antibodies for 1 h at room temperature. Nuclei were counterstained with Prolong Gold + DAPI (Invitrogen, concentration 5 µg/ml).

Antibodies were used as follows:

**Table:** 

Antibody	Code	Species	Dilution	Provider
mVegfr3	AF743	Goat	1:25	R&D Systems
mProx1	AF2727	Goat	1:2,000	R&D Systems
mLyve1	ab14917	Rabbit	1:250	Abcam
CD31	ab28364	Rabbit	1:250	Abcam
MDK	sc‐46701	Mouse	1:50	Santa Cruz Biotechnology

### Lymphatic and blood vessel quantification and pseudocoloring

For tumor and lung lymphatic and blood vessel quantifications in tumors and lung sections, pictures were captured using an Olympus AX70 microscope (10× objective). The density of lymphatic endothelial cells was defined by dual Lyve1/Prox1 staining. Blood vessels were visualized with an anti‐CD31 antibody (ab14917, Abcam). The amount of positive cells was estimated by different investigators blind to experimental conditions. A minimum of 4 areas per mice and organ and 3 mice per condition were counted.

In the case of lymph nodes, paraffin‐embedded sections were stained for Lyve1 and Prox1 as indicated before and scanned using an Axio Scan.Z1 slide scanner using a 40× objective (Zeiss, Oberkochen, Germany). The number of positively stained pixels for Lyve1/Prox1 in relation to the total number of pixels in the lymph node image was quantified using Fiji (ImageJ) software (Rasband, W.S., ImageJ, U. S. National Institutes of Health, Bethesda, Maryland, USA). When indicated, cells with dual Lyve1 and Prox1 staining were pseudocolored using Photoshop CC2019 (Adobe Inc.) color‐replace tool.

### Quantitative reverse transcription–PCR

RNA purification from melanoma tissue samples and real‐time reverse transcription–PCR (qRT–PCR) were performed essentially as described (Tormo *et al*, 2009) using the following primers:

**Table:** 

hVEGF‐C	hVEGF‐C‐F	TGCCAGCAACACTACCACAG
	hVEGF‐C‐R	GTGATTATTCCACATGTAATTGGTG
hVEGF‐D	hVEGF‐D‐F	GGAGGAAAATCCACTTGCTG
	hVEGF‐D‐R	GCAACGATCTTCGTCAAACA
hVEGF‐R3 (Flt4)	hVEGF‐R3‐F	CAAGAAAGCGGCTTCAGGTA
	hVEGF‐R3‐R	GCAGAGAAGAAAATGCTGACG
IFN α2	hIFNA2‐F	TCCTGCTTGAAGGACAGACA
	hIFNA2‐R	TCCTGCTTGAAGGACAGACA
IFN β1	hIFNB1‐F	GCTAGAGTGGAAATCCTAAG
	hIFNB1‐R	ACAGCATCTGCTGGTTGAAG
MDA5	hMDA5‐F	GCGCACACCGCAGAGTCCAA
	hMDA5‐R	TCCACAGGGCTCTCAGGCCG
18S	18S‐F	TTGGAGGGCAAGTCTGGTG
	18S‐R	CCGCTCCCAAGATCCAACTA

### Functional analyses in human lymphatic endothelial cells (HLECs)

Normal human lymphatic microvascular endothelial cells (HMVEC‐dLy) (Lonza, MD) referred in the text as HLEC were grown as tissue culture monolayers in EGM™‐2 medium supplemented with MV BulletKit (Lonza). For tube formation assays, three‐dimensional cultures were prepared with 2.5 × 10^5^ cells seeded in MW6 plates covered by a layer of Matrigel (BD, NJ). When indicated, cells were pretreated for 12 h with 0.5 µg/ml BO‐110 or vehicle control. Treatment was maintained thereafter. Pictures were acquired 10 h after seeding the cells. Time‐lapse videos were acquired in the same experimental conditions using a Leica Thunder widefield microscope with a 10× objective 0.45NA and LASX v3.7 acquisition software. Images were captured every 45 min. Videos were processed using Fiji (ImageJ) software. For fluorescence detection of HLEC in this time‐lapse imaging, cells were labeled with CellTracker™ Green CMFDA Dye (Thermo Fisher, C7025, Waltham, MA) following the manufacturer’s instructions.

### Proteomic analyses: iTRAQ and LC‐MS/MS

For proteomic analyses, cells were cultured for 24 h in 10‐cm culture plates and then treated with 0.5 µg/ml B0‐110 or vehicle control. For total cell extract profiling, proteins were subjected to isobaric labeling analysis using iTRAQ 8‐plex as described before (Perez‐Guijarro *et al*, 2016), using isobaric amine‐reactive tag according to the manufacturer's instructions (AB SCIEX). Labeled samples were pooled and evaporated in a vacuum centrifuge. The sample was cleaned up using a Sep‐Pak C18 cartridge (Waters). Eluted peptides were vacuum‐dried and reconstituted in OFFGEL solution before electrofocusing with a 3100 OFFGEL Fractionator (Agilent). 24 fractions were collected. Peptides were separated by RP chromatography using a nanoLC Ultra System (Eksigent), directly coupled with an Impact (Bruker) mass spectrometer, equipped with a CaptiveSpray ion source. Two micrograms of each fraction was loaded onto a reversed‐phase C18, 5 µm, 0.1 × 20 mm trapping column (NanoSeparations). The peptides were eluted at a flow rate of 300 nl/min onto an analytical column packed with ReproSil‐Pur C18‐AQ, 2.4 μm, 75 μm × 50 cm (Dr. Maisch GmbH), heated to 45°C. Solvent A was 4% ACN in 0.1% FA and Solvent B acetonitrile in 0.1% FA. Peptides were separated using the following gradient 0–2 min 2% B, 2–119 min 2–20% B, 119–129 min 20‐34% B, 129–140 min 98% B, and 140–145 min 2% B. The spray voltage was set to 1.35 kV, and the temperature of the source was set to 180°C. The MS survey scan was performed at a spectrum rate of 2.5 Hz in the TOF analyzer (80–1,600 *m/z*). The minimum signal for triggering MS/MS was set to 500 counts. The 20 most abundant isotope patterns with z ≥ 2 and *m/z* > 350 from the survey scan were sequentially isolated and fragmented using a collision energy of 23–56 eV as a function of the *m/z* value. Dynamic exclusion was set to 30 s using the *rethinking* option. Raw data were processed using Proteome Discoverer 1.4 and MaxQuant 1.5 using standard workflows, and results were filtered at 1% FDR.

### Assessment of cell death

Apoptosis was determined by staining cells with Annexin‐V‐APC (BD Biosciences Pharmingen, San Jose, CA, USA), TMRE (tetramethylrhodamine ethyl ester; Sigma), and DAPI (4′,6‐diamidino‐2‐phenylindole; Sigma). To this end, 4 × 10^5^ cells were seeded in 6‐well plates (Corning, NY, USA) and were incubated with BO‐110 at the doses stated in the figure legends. After the indicated times, floating and adherent cells (these detached with trypsin) were centrifuged for 5 min at 300 *g*, followed by a PBS wash. Cells were then resuspended in prewarmed TMRE (37°C) 40 nM in 1× PBS and incubated for 10 min in the dark. After centrifuging a 5‐min spin at 300 *g*, the cells were resuspended in 200 µl of Annexin‐V binding buffer (10 mM HEPES/NaOH pH 7.4, 140 mM NaCl, 2.5 mM CaCl_2_) containing 0.1 µg/ml Annexin‐V and 0.5 µg/ml PI, and incubated for 20 min at 4°C. Cells were then counterstained with DAPI 1 µg/ml and analyzed by flow cytometry with a FACSCalibur (Becton, Dickinson and Company, BD Biosciences; USA). Data were analyzed using FlowJo Software V.10 (Tree Star Inc., Ashland, OR, USA).

### MIDKINE and Flt4 (VEGFR3) promoter assays

The *MDK* promoter (encompassing 1,063 bp upstream and 204 downstream of the Midkine starting site; NM_001012333; chr11+: 46358816‐46360083) and the *Flt4* promoter (encompassing 1364 bp upstream and 19 downstream of the *Flt4* starting site; NM_002020; chr5‐: 180009187‐180010570) were cloned into pNL2.1 vector (#N1061; Promega, MA). These reporter plasmids were transfected into SK‐Mel‐147 cells or HLEC. Cells were co‐transduced with pGL4.52‐Luc2 (#E1320; Promega) vector as transfection control. Luciferase activity was monitored 16 h thereafter (in the presence or absence of BO‐110) using the Nano‐Glo^®^ Dual‐Luciferase assay (#N1610; Promega). Cell viability was kept over 80% in all conditions analyzed.

### Type I IFN blocking assays

For type I interferon blocking assays, human IFN‐β blocking antibody (Clone AF814; R&D) and its corresponding isotype (Goat IgG, R&D Systems) were used at a concentration of 0.2 µg/ml. Anti‐Interferon‐α/β Receptor Chain 1 Antibody (IFNAR1) (clone MAR1‐5A3; Biolegend, CA) and its corresponding isotype (Mouse IgG2a, clone GC270; Millipore) were used at 0.1 µg/ml. Where indicated, these reagents were added simultaneously with BO‐110 to the culture media.

### ELISA analyses of IFN‐β and Midkine in mouse plasma

For the analysis of Ifn‐β and mouse Midkine in plasma, 5 × 10^5^ B16R2L cells (Liersch *et al*, 2012) were injected in the flank of 14‐week‐old female *Vegfr3^Luc^* immunocompetent mice. For circulating Ifn‐β and human Midkine, 1 × 10^6^ SK‐Mel‐147 cells were injected in the flank of 14‐week‐old female *Vegfr3^Lu^*; *nu/nu* mice as previously reported (Olmeda *et al*, 2017). In all cases, tumor growth was recorded by measuring the two orthogonal external diameters using a caliper. Tumor volume was calculated using the formula (a × b^2^ × 0.52). *Vegfr3‐Luc* emission was measured in an IVIS system as indicated above. When tumors reached a size of 100 mm^3^, mice were randomized and treated with 0.8 mg/kg BO‐110 or vehicle control. 500 µl of blood was extracted 8 h after the indicated treatments. Blood was then allowed to clot at room temperature for 30 min. The clot was removed by spinning at 2,000 ×*g* for 10 min in a refrigerated centrifuge. The supernatant was collected and stored at −80°C until analysis. ELISA determination of Midkine and Ifn‐β levels was performed in duplicates using the following kits: Mouse Mdk (Mouse Midkine ELISA Kit PicoKine™; EK2015, Boster, Pleasanton, CA), Human MDK (Human Midkine ELISA Development Kit, 900‐K190, Peprotech Rocky Hill, NJ), and mouse Ifn‐β (Mouse IFN‐β DuoSet^®^ ELISA, DY8234, R&D Systems).

### Bioinformatics analyses

GSEA tests were performed using GSEA 4.1 software (Broad Institute). Gene set collections were retrieved from annotations of the Broad Institute Library of Molecular Signature Databases v7.2 (MSigDB). Gene sets were tested for false discovery rate (FDR). After the Kolmogorov–Smirnoff correction for multiple testing, only those pathways with FDR < 0.25 were considered as significant. Heatmaps were created by Morpheus Heatmaps (https://software.broadinstitute.org/morpheus).

### Statistical analyses

Cell proliferation and tumor growth were analyzed by one‐way and two‐way ANOVA, respectively. Survival curves were estimated with the Kaplan–Meier product‐limit method and compared using logrank test. *P*‐values are indicated in each figure, with *P* < 0.05 considered significant. For *t*‐test parametric analyses, the normal distribution of the data was tested using the Shapiro–Wilk test. Statistical processing of transcriptomic and proteomic data was performed as previously described (Tormo *et al*, 2009; Karras *et al*, 2019). All statistical analyses were performed using GraphPad 8 software.

## Author contributions

MSS and DO conceived and designed all the studies in this work. SO conceived and developed the *Vegfr3^EGFPLuc^* (*Flt4^tm1.1Sgo^*) and *Vegfr3^Luc^ nu/nu* mouse models. DO developed the protocols for the analysis of tumor‐induced lymphangiogenesis *in vivo* (pre‐ and post‐surgery, and pre–post‐treatment with BO‐110), and was in charge of the histological assessment of Vegfr3, Lyve1, Prox1, and CD31. DC‐W contributed to the antimelanoma drug treatment experiments and performed the treatments on *Ifnar1*‐deficient models; she helped with data analysis of the *in vivo* experiments and revised the manuscript. PO‐R and JLR‐P provided fresh melanoma biopsies for the generation of PDX. NI and JM contributed to data analysis. TGC and EC were in charge of animal breeding and genotyping and provided technical assistance. CM cloned *MDK* and *FLT4* promoters. DA‐C and CM contributed with technical assistance. The manuscript was written by MSS and DO, revised by SO and DA‐C, and approved by all authors. MSS and DO supervised the project.

## Conflict of interest

María S. Soengas is a co‐founder of BiOncotech Therapeutics (now Highlight Therapeutics), a biotechnology company interested in the development of dsRNA‐based treatments for aggressive cancers. She does not sit on the administrative board nor is she involved in decisions regarding clinical trials or commercial development of Highlight Therapeutics's compounds.

## Supporting information



Expanded View Figures PDF

Dataset EV1

Dataset EV2

Dataset EV3

Dataset EV4

Dataset EV5

Dataset EV6

Movie EV1

Source Data for Expanded View

Source Data for Figure 1

## Data Availability

The cDNA array data for BO‐119 discussed in this publication were generated as previously reported (Tormo *et al*, 2009), and are deposited in NCBI's Gene Expression Omnibus database, with accession number GSE14445. The RNAseq data are deposited also in NCBI's Gene Expression Omnibus database, with accession number GSE180629 (https://www.ncbi.nlm.nih.gov/geo/). The mass spectrometry proteomics data have been deposited to the ProteomeXchange Consortium via the PRIDE partner repository with the dataset identifier PXD007000 (https://www.ebi.ac.uk/pride/).
