# Supplementary material for: Live imaging of neolymphangiogenesis identifies acute antimetastatic roles of dsRNA mimics
Source: EMBO Mol Med. 2021 Nov 11;13(12):e12924. doi: 10.15252/emmm.202012924 (PMC8649872; doi:10.15252/emmm.202012924)
Supplement: Supplementary file 1 — Expanded View Figures PDF [file EMMM-13-e12924-s002.pdf]

Expanded View Figures

Figure EV1. Identification of BO-110 as an antilymphangiogenic agent in *MetAlert mice*, with controls for selective inhibition of Vegfr3-Luc signaling.

A Impact of BO-110 on *Tyr:CreERT2*; *BRAF*^{V600E};*Pten*$^{flox/flox}$; *Vegfr3*^{Luc} mice. Upper panels correspond to optical photographs of animals treated with vehicle or with 6 doses of BO-110 (0.8 mg/kg, twice a week, 3 weeks), and depilated to ease in the imaging. These same animals are shown in the bottom panels for luciferase emission centering on the tumor (1, 3) or on sentinel LN (2, 4). Scale, p/s/cm^2/sr (×10^6).

B Histological assessment of the impact of BO-110 (BO) on neolymphangiogenesis in melanomas generated in *Vegfr3*^{Luc};*Tyr::CreERT2*;*BRAF*^{V600E};*Pten*$^{fl/fl}$*Pten*$^{flox/flox}$ mice. Panels correspond to Lyve1 detected by immunohistochemistry (IHC, brown signal) or to dual fluorescence staining for Lyve1 (red) or Vegfr3 (green). Right panel corresponds to merged signal of Lyve1 and Vegfr3 IF.

C Right panel, histological visualization of lymphatic vessel density by costaining for Lyve1 (blue) and Prox1 (purple) in representative lungs of *Vegfr3*^{Luc};*Tyr::CreERT2*; *Braf*^{V600E};*Pten*$^{flox/flox}$ melanomas treated with vehicle (V) or 4 doses of BO-110 (BO, 0.8 mg/kg). Left panel, pseudocoloring in red of overlapped staining with anti-Lyve1 and Prox1 antibodies (cells positive for both markers are highlighted with red arrowheads).

D Right panel, histological analyses of lymphatic vessel density by Lyve1 and Prox1 dual staining in representative lymph nodes of animals in Fig 2B processed at the endpoint of the experiment (four doses of BO-110 or vehicle control). Left panel, dual-positive Lyve1 and Prox1 cells pseudocolored in red.

E Quantification of lymphatic vessel (left) and blood vessel (right) density in lymph nodes of animals in Fig 2B (SK-Mel-147 tumors) processed at the endpoint of the experiment (four doses of BO-110 or vehicle control). Data correspond to the quantification of four fields per tumor, performed in biological triplicates. Statistical significance was determined by the Mann–Whitney *t*-test.

F *In vivo* imaging of the comparative impact of BO-110 (0.8 mg/kg) (BO) or vehicle (V) on luciferase emission driven from the Vegfr3^{Luc} MetAlert mice bearing a SK-Mel-147 tumor, or from an unrelated promoter (SV40-Luc) stably expressed in SK-Mel-147 melanoma cells.

G Quantification of luciferase emission 24 h after treatment in the tumor and the indicated organs in animals treated as in (F). $N = 6$ mice per condition. Boxplots show the median, 25th and 75th percentiles, and the maximum and minimum signal. Luciferase signal was normalized to the corresponding vehicle control. Statistical significance was determined by ANOVA.

Source data are available online for this figure.

© 2021 The Authors

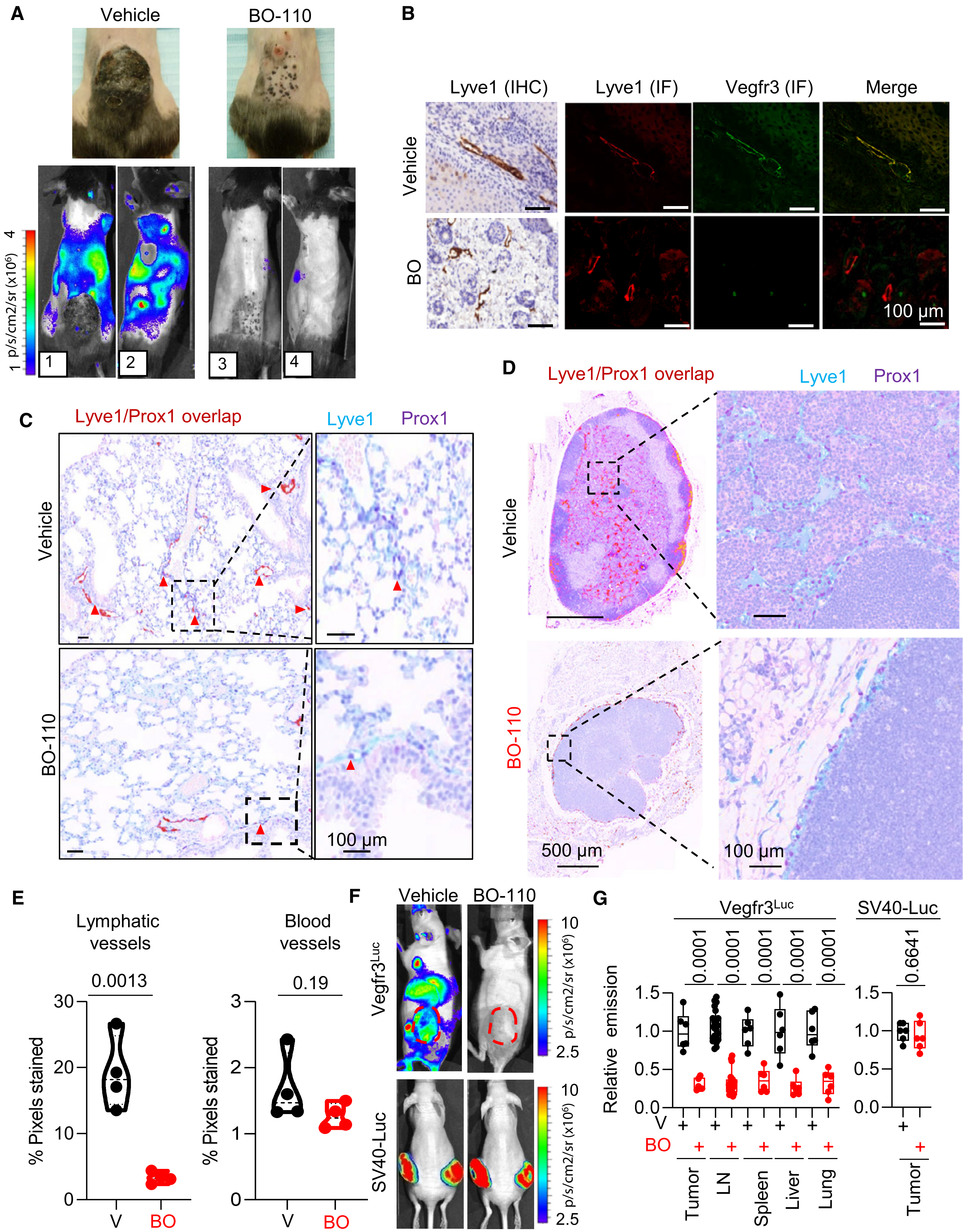
A
Vehicle
BO-110
p/s/cm2/sr (x10^6)
1
4
1
2
3
4
B
Lyve1 (IHC)
Lyve1 (IF)
Vegfr3 (IF)
Merge
Vehicle
BO
100 µm
C
Lyve1/Prox1 overlap
Lyve1
Prox1
Vehicle
BO-110
100 µm
D
Lyve1/Prox1 overlap
Lyve1
Prox1
Vehicle
BO-110
500 µm
100 µm
E
Lymphatic vessels
Blood vessels
0.0013
0.19
% Pixels stained
V
BO
F
Vehicle
BO-110
Vegfr3Luc
SV40-Luc
p/s/cm2/sr (x10^6)
10
2.5
G
Vegfr3Luc
SV40-Luc
0.0001
0.6641
Relative emission
V
BO
Tumor
LN
Spleen
Liver
Lung

Figure EV1.

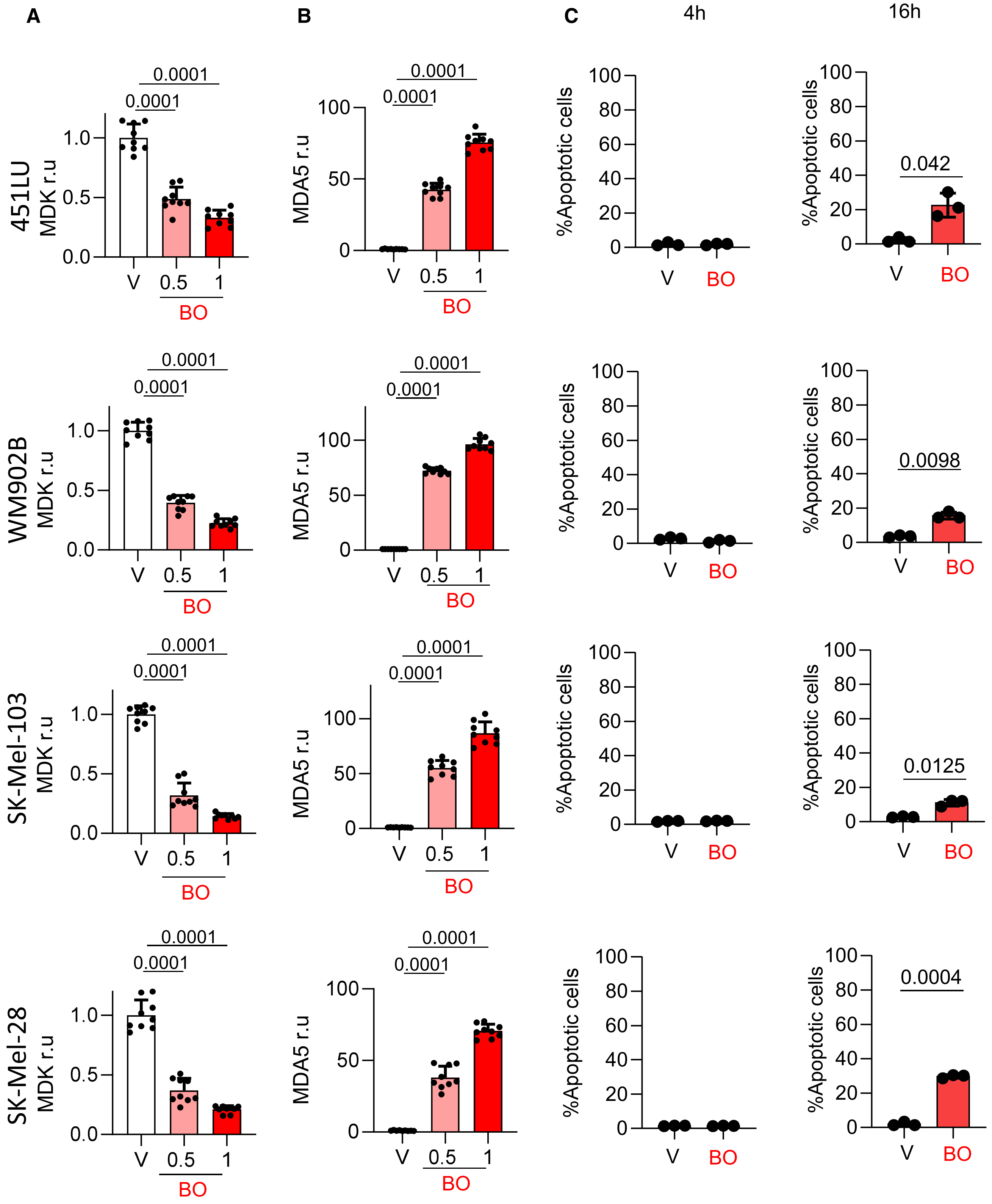
A
B
C
4h
16h
451LU
MDK r.u
MDA5 r.u
%Apoptotic cells
0.0001
0.042
0.0098
0.0125
0.0004
WM902B
SK-Mel-103
SK-Mel-28
V
0.5
1
BO

Figure EV2.

Figure EV2. MDA5 induction and Mdk repression by BO-110 in a panel of melanoma cell lines.

A Inhibitory effect of the indicated doses of BO-110 (in μg/ml) on *MDK* mRNA expression determined by qRT–PCR in the indicated melanoma cell lines 16 h after treatment. Data correspond to average mRNA levels of three experiments with three technical replicates normalized to vehicle control (V) ± SD. Statistical significance was determined by ANOVA.

B qRT–PCR analysis of relative mRNA levels of *MDA5* 16 h after treatment of the indicated melanoma cell lines with the 0.5 or 1 μg/ml of BO-110 (BO) or vehicle (V). Data correspond to the mean ± SD of three experiments with three technical replicates. Statistical significance was determined by ANOVA.

C Percentage of apoptotic cells (Annexin-V staining detection by flow cytometry) after treatment of the indicated cell lines with vehicle (V) or 0.5 μg/ml BO-110 (BO) for the indicated time points. Data correspond to the mean ± SD of three experiments. Statistical significance was determined by the *t*-test.

Figure EV3. Omic analyses of the BO-110 mechanism of action.

A Heatmap showing differentially deregulated signaling cascades in SK-Mel-147 treated with 1 μg/ml B0-110 for 4 or 10 h (versus vehicle-treated controls). Data correspond to mRNA expression profiles analyzed by GSEA using the Hallmark gene sets. The scale indicates the normalized enrichment score (NES).

B Venn diagrams depicting common and specific Hallmarks gene sets significantly enriched in a GSEA in cell lines SK-Mel-147 and HLEC upon BO-110 treatment (left: upregulated; right: downregulated; FDR < 0.25). See also Datasets EV3 and EV4 for additional detail.

C Venn diagrams as in B, but estimated for REACTOME gene sets. See also Datasets EV5 and EV6 for the complete gen set list.

D Enrichment plots for the indicated signaling cascades gene sets upon BO-110 treatment in SK-Mel-147 or in HLEC.

 © 2021 The Authors

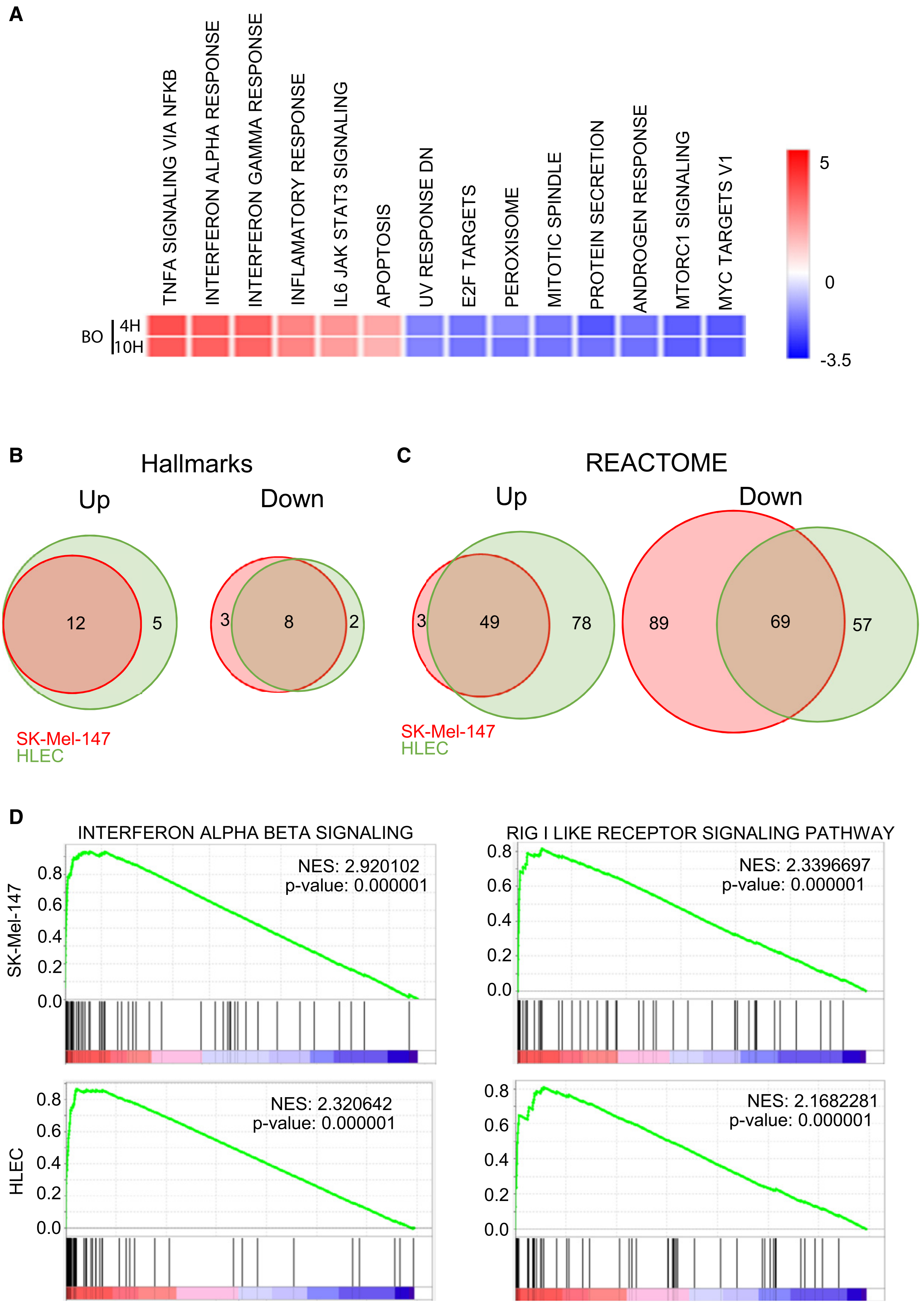
A
TNFA SIGNALING VIA NFKB
INTERFERON ALPHA RESPONSE
INTERFERON GAMMA RESPONSE
INFLAMATORY RESPONSE
IL6 JAK STAT3 SIGNALING
APOPTOSIS
UV RESPONSE DN
E2F TARGETS
PEROXISOME
MITOTIC SPINDLE
PROTEIN SECRETION
ANDROGEN RESPONSE
MTORC1 SIGNALING
MYC TARGETS V1
BO
4H
10H
5
0
-3.5
B
Hallmarks
Up
Down
12
5
3
8
2
SK-Mel-147
HLEC
C
REACTOME
Up
Down
3
49
78
89
69
57
SK-Mel-147
HLEC
D
INTERFERON ALPHA BETA SIGNALING
RIG I LIKE RECEPTOR SIGNALING PATHWAY
SK-Mel-147
HLEC
NES: 2.920102
p-value: 0.000001
NES: 2.3396697
p-value: 0.000001
NES: 2.320642
p-value: 0.000001
NES: 2.1682281
p-value: 0.000001
0.8
0.6
0.4
0.2
0.0

Figure EV3.

Figure EV4. IFN-dependent inhibition of lymphangiogenesis by BO-110.

A Binding sites for experimentally validated IRF-related transcription factors in the promoters of *MDK* and *FLT4* (*VEGFR3*). Data from Interferome and TRANSFAC (www.genexplain.com).

B BO-110-driven blockade of the tube-forming capacity of HLEC and rescue with anti-IFNAR1 blocking antibodies (anti-IFN-β and anti-INFAR1) or corresponding controls. Images correspond to cells plated in Matrigel and imaged 8 h after treatment with 0.5 μg/ml BO-110.

C Histological analyses of lymphatic vessel density defined by dual staining for Lyve1 (blue) and Prox1 (purple). To facilitate the visualization, cells positive for both markers were pseudocolored in red. Large magnifications of the doted squared areas can be found in Fig 5E.

D Quantification of Lyve1/Prox1 staining in lymph nodes of animals in Fig 5D and E, processed at the endpoint of the experiment (4 doses of BO-110 or vehicle control). Data correspond to the mean ± SD of four biological replicates. Statistical significance was determined by the *t*-test.

Source data are available online for this figure.

© 2021 The Authors

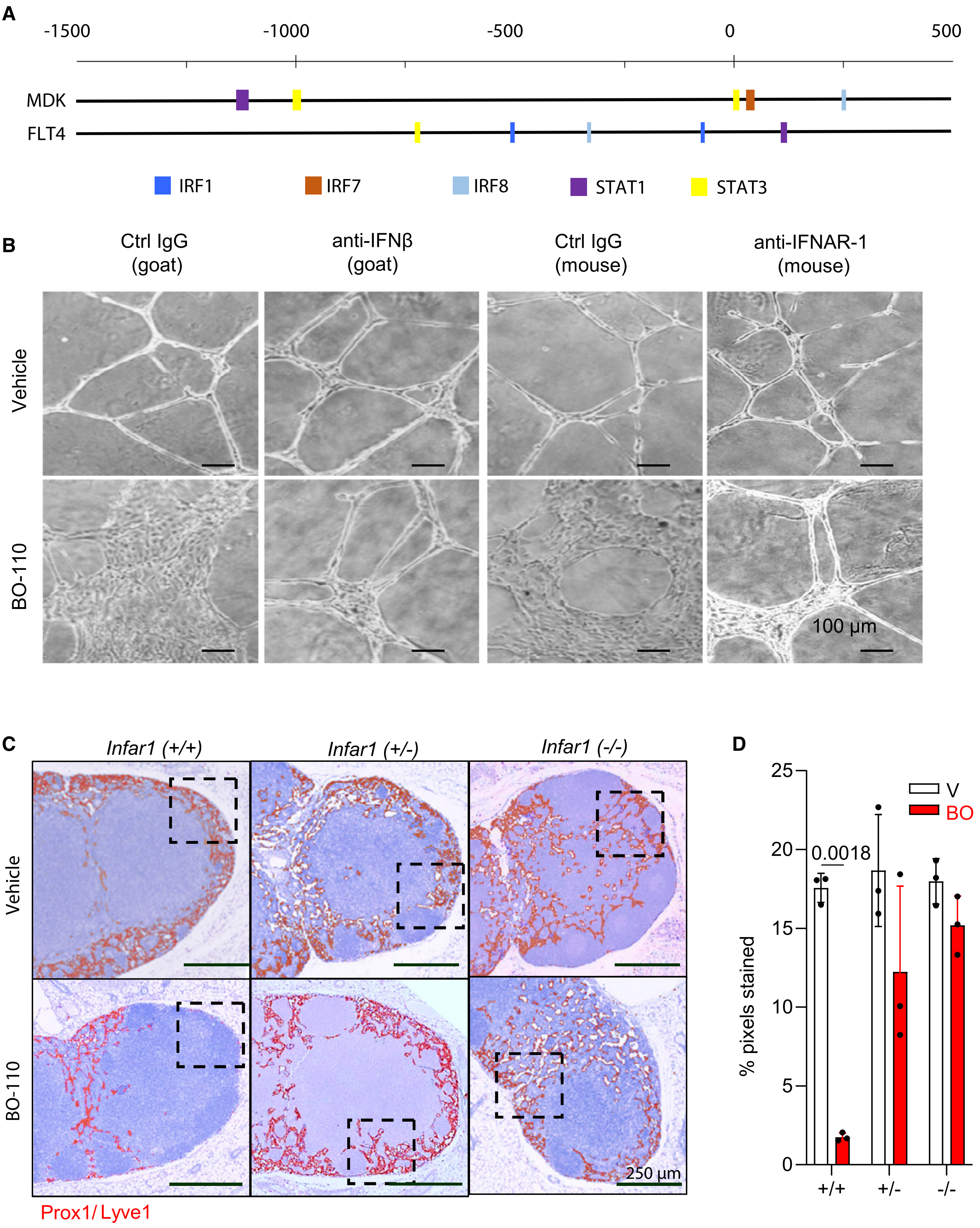
A
-1500
-1000
-500
0
500
MDK
FLT4
IRF1
IRF7
IRF8
STAT1
STAT3
B
Ctrl IgG (goat)
anti-IFNβ (goat)
Ctrl IgG (mouse)
anti-IFNAR-1 (mouse)
Vehicle
BO-110
100 μm
C
Infar1 (+/+)
Infar1 (+/-)
Infar1 (-/-)
Vehicle
BO-110
250 μm
Prox1/ Lyve1
D
% pixels stained
0
5
10
15
20
25
V
BO
0.0018
+/+
+/-
-/-

Figure EV4.

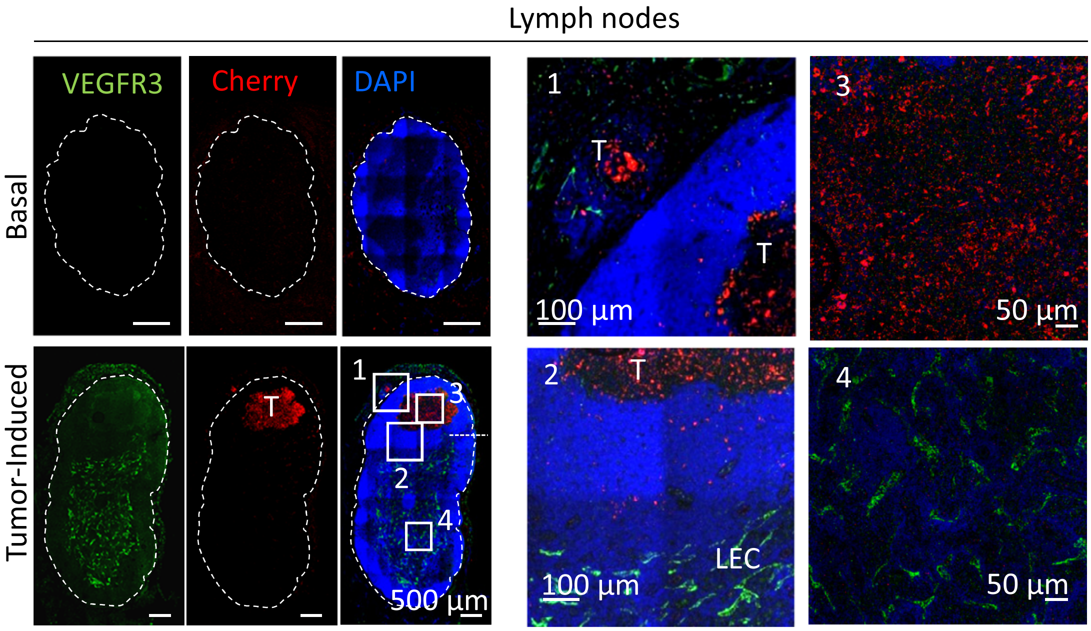

Figure EV5. Disseminated tumor cells at lymph node and lung lymphovascular metastatic niches.

Histological analysis of lymph nodes in basal conditions or in mice implanted s.c with mCherry-labeled SK-Mel-147 performed before tumor excision as in Fig 7A. Vegfr3 and mCherry were detected by immunofluorescence (green and red signaling, respectively). Panels 1–4 correspond to a larger magnification of different areas in these lymph nodes. Note that Vegfr3 concentrates in lymphatic endothelial cells (LECs), with undetectable levels in melanoma cells. T, tumor cells.

 © 2021 The Authors
