# Supplementary material for: Live imaging of neolymphangiogenesis identifies acute antimetastatic roles of dsRNA mimics
Source: EMBO Mol Med. 2021 Nov 11;13(12):e12924. doi: 10.15252/emmm.202012924 (PMC8649872; doi:10.15252/emmm.202012924)
Supplement: Supplementary file 8 — Movie EV1 [file EMMM-13-e12924-s001.zip › Movie EV1 legend.docx]

**Movie EV1:** BO-110-driven blockade of the tube-forming capacity of HLEC and rescue with anti-IFNAR1 blocking antibodies. Images correspond to cells plated in matrigel and imaged every 45 minutes after treatment with 0.5 µg/ml BO-110. See also EV Fig. 4B, for additional results with anti-IFNAR1 and anti-IFNb blocking antibodies.
