# Supplementary figures and images for: Live imaging of neolymphangiogenesis identifies acute antimetastatic roles of dsRNA mimics
Source: EMBO Mol Med. 2021 Nov 11;13(12):e12924. doi: 10.15252/emmm.202012924 (PMC8649872; doi:10.15252/emmm.202012924)

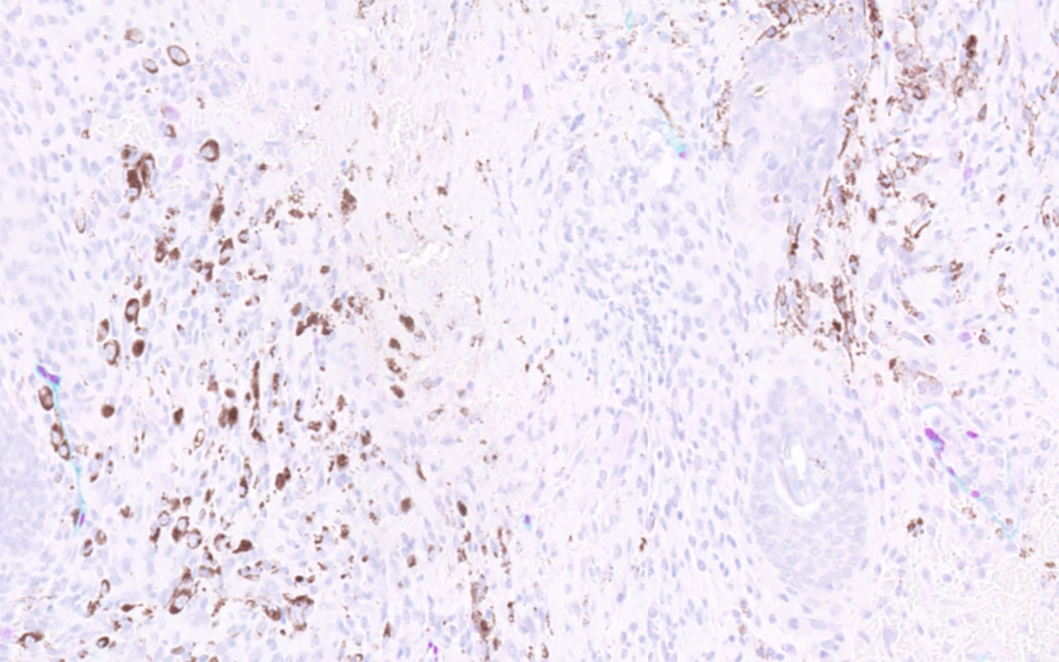

Supplement: Supplementary file 10 — Source Data for Figure 1 [file EMMM-13-e12924-s011.zip › Figure 1D/BO110.jpg]

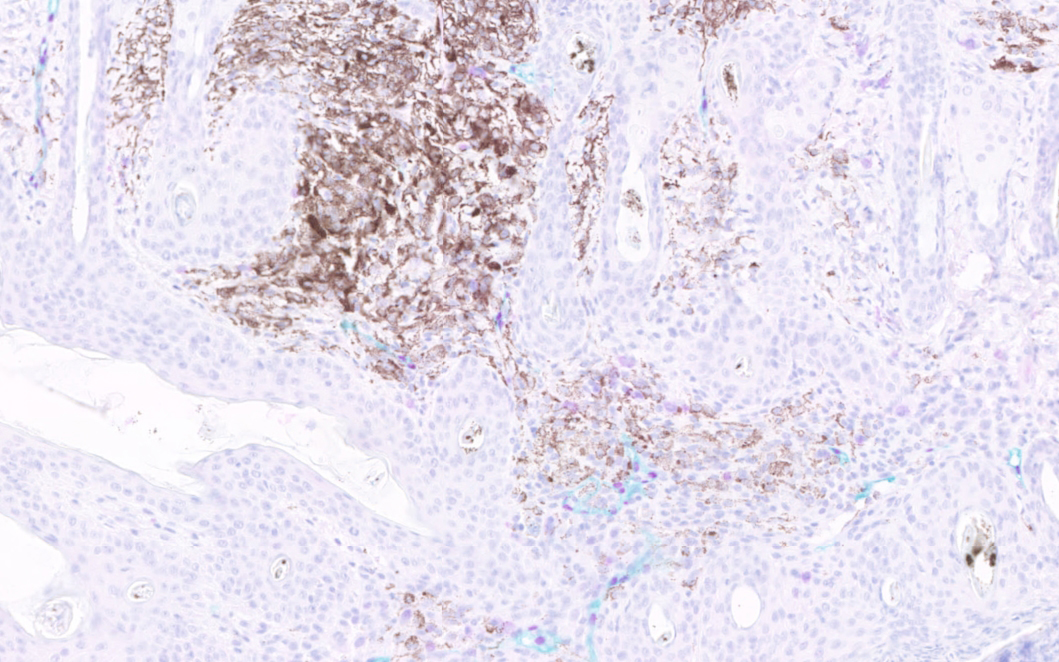

Supplement: Supplementary file 10 — Source Data for Figure 1 [file EMMM-13-e12924-s011.zip › Figure 1D/Vehicle.jpg]
