# Supplementary material for: Live imaging of neolymphangiogenesis identifies acute antimetastatic roles of dsRNA mimics
Source: EMBO Mol Med. 2021 Nov 11;13(12):e12924. doi: 10.15252/emmm.202012924 (PMC8649872; doi:10.15252/emmm.202012924)
Supplement: Supplementary file 11 [file EMMM-13-e12924-s004.pdf]

EMBO Molecular Medicine

# Live imaging of neolymphangiogenesis identifies acute antimetastatic roles of dsRNA mimics

David Olmeda, Daniela Cerezo-Wallis, Cynthia Mucientes, Tonantzin Calvo, Estela Cañón, Direna Alonso-Curbelo, Nuria Ibarz, Javier Muñoz, José Luis Rodriguez-Peralto, Pablo Ortiz-Romero, Sagrario Ortega, and Maria Soengas
**DOI: 10.15252/emmm.202012924**

*Corresponding authors: Maria Soengas (msoengas@cnio.es) , David Olmeda (dolmeda@cnio.es)*



## Review Timeline:

| | |
|---|---|
| Submission Date: | 11th Jun 20 |
| Editorial Decision: | 10th Jul 20 |
| Revision Received: | 6th Sep 21 |
| Editorial Decision: | 6th Oct 21 |
| Revision Received: | 12th Oct 21 |
| Accepted: | 15th Oct 21 |

*Editor: Lise Roth*


## Transaction Report:

(Note: With the exception of the correction of typographical or spelling errors that could be a source of ambiguity, letters and reports are not edited. Depending on transfer agreements, referee reports obtained elsewhere may or may not be included in this compilation. Referee reports are anonymous unless the Referee chooses to sign their reports.)

10th Jul 2020

Dear Dr. Soengas,

Thank you for the submission of your manuscript to EMBO Molecular Medicine. We have now received feedback from the three reviewers who agreed to evaluate your manuscript. As you will see from the reports below, the referees acknowledge the interest and novelty of the study, and are overall supporting publication of your work pending appropriate major revisions.

In particular, the discrimination between the therapeutic and adjuvant therapy modalities needs to be precisely presented and requires a better analysis of the metastasis and lymphatics in your mouse model over time. Additionally, further investigation into the mechanism of action of BO-110 is required.

Addressing these and the other reviewers' concerns in full will be necessary for further considering the manuscript in our journal, and acceptance of the manuscript will entail a second round of review. EMBO Molecular Medicine encourages a single round of revision only and therefore, acceptance or rejection of the manuscript will depend on the completeness of your responses included in the next, final version of the manuscript. For this reason, and to save you from any frustrations in the end, I would strongly advise against returning an incomplete revision.

***
When submitting your revised manuscript, please carefully review the instructions that follow below. Failure to include requested items will delay the evaluation of your revision:

1) A .docx formatted version of the manuscript text (including legends for main figures, EV figures and tables). Please make sure that the changes are highlighted to be clearly visible.

2) Individual production quality figure files as .eps, .tif, .jpg (one file per figure).

3) A .docx formatted letter INCLUDING the reviewers' reports and your detailed point-by-point responses to their comments. As part of the EMBO Press transparent editorial process, the point-by-point response is part of the Review Process File (RPF), which will be published alongside your paper.

4) A complete author checklist, which you can download from our author guidelines (https://www.embopress.org/page/journal/17574684/authorguide#submissionofrevisions). Please insert information in the checklist that is also reflected in the manuscript. The completed author checklist will also be part of the RPF.

5) Please note that all corresponding authors are required to supply an ORCID ID for their name upon submission of a revised manuscript.

6) We would also encourage you to include the source data for figure panels that show essential data. Numerical data should be provided as individual .xls or .csv files (including a tab describing the data). For blots or microscopy, uncropped images should be submitted (using a zip archive if multiple images need to be supplied for one panel). Additional information on source data and instruction on how to label the files are available at
.

8) We replaced Supplementary Information with Expanded View (EV) Figures and Tables that are collapsible/expandable online. A maximum of 5 EV Figures can be typeset. EV Figures should be cited as 'Figure EV1, Figure EV2" etc... in the text and their respective legends should be included in the main text after the legends of regular figures.

- For the figures that you do NOT wish to display as Expanded View figures, they should be bundled together with their legends in a single PDF file called *Appendix*, which should start with a short Table of Content. Appendix figures should be referred to in the main text as: "Appendix Figure S1, Appendix Figure S2" etc.

- Additional Tables/Datasets should be labeled and referred to as Table EV1, Dataset EV1, etc. Legends have to be provided in a separate tab in case of .xls files. Alternatively, the legend can be supplied as a separate text file (README) and zipped together with the Table/Dataset file.
See detailed instructions here:
.

9) For more information: There is space at the end of each article to list relevant web links for further consultation by our readers. Could you identify some relevant ones and provide such information as well? Some examples are patient associations, relevant

databases, OMIM/proteins/genes links, author's websites, etc...

10) Every published paper now includes a 'Synopsis' to further enhance discoverability. Synopses are displayed on the journal webpage and are freely accessible to all readers. They include a short stand first (maximum of 300 characters, including space) as well as 2-5 one-sentences bullet points that summarizes the paper. Please write the bullet points to summarize the key NEW findings. They should be designed to be complementary to the abstract - i.e. not repeat the same text. We encourage inclusion of key acronyms and quantitative information (maximum of 30 words / bullet point). Please use the passive voice. Please attach these in a separate file or send them by email, we will incorporate them accordingly.

Please also suggest a striking image or visual abstract to illustrate your article. If you do please provide a png file 550 px-wide x 400-px high.

11) As part of the EMBO Publications transparent editorial process initiative (see our Editorial at http://embomolmed.embopress.org/content/2/9/329), EMBO Molecular Medicine will publish online a Review Process File (RPF) to accompany accepted manuscripts.

In the event of acceptance, this file will be published in conjunction with your paper and will include the anonymous referee reports, your point-by-point response and all pertinent correspondence relating to the manuscript. Let us know whether you agree with the publication of the RPF and as here, if you want to remove or not any figures from it prior to publication.

Please note that the Authors checklist will be published at the end of the RPF.

EMBO Molecular Medicine has a "scooping protection" policy, whereby similar findings that are published by others during review or revision are not a criterion for rejection. Should you decide to submit a revised version, I do ask that you get in touch after three months if you have not completed it, to update us on the status.

I look forward to receiving your revised manuscript.

Yours sincerely,

Lise Roth

Lise Roth, PhD
Editor
EMBO Molecular Medicine

***** Reviewer's comments *****

Referee #1 (Comments on Novelty/Model System for Author):

The "MetAlert" mouse model is a very elegant approach to visualize and monitor lymphatic premetatastatic niche formation.

Referee #1 (Remarks for Author):

Olmeda and colleagues present a significant study which is of great interest for melanoma research.
A Previously published mouse model (Olmeda et al., Nature, 2017), which is expressing an eGFP-luciferase cassette under the control of the VEGFR3 promotor, was used as a tool to study lymphatic premetastatic niche formation. Drug testing of a double-stranded RNA (dsRNA) mimic, BO-110, was then performed in two melanoma models, an inducible Tyr:CreERT2;BRAFV600E;Ptenflox/flox and a xenotransplant model in nude mice. Taking advantage of the highly sensitive bioluminescence imaging technique this mouse model is a very elegant approach to visualize and monitor the lymphatic premetatastatic niche.
Besides the overwhelming success of immunotherapy or checkpoint blockade for melanoma therapy, the authors clearly state that there is still unmet need in melanoma research, since many patients develop therapy resistances or suffer from severe therapy toxicities. In the clinical setting, immunotherapy is used for the treatment of stage IV melanoma patients but is also approved by the FDA and EMA as adjuvant therapy. By choosing two adequate mouse models even these two therapy modalities are carefully considered and addressed.
The overall concept of the study is well designed and of great novelty for the field of melanoma research.

Thus, I recommend this study for publication in EMBO Molecular Medicine. However, there are certain major limitations that I would like to mention and like to be addressed by the authors.
- The discrimination between the therapeutic and adjuvant therapy modality needs to be precisely presented.
Your previous publication (Olmeda et al., 2017, Nature - Figure 2) indicates that mice start to develop LN metastases at around 2 weeks and distant metastases at around 4 weeks in the xenotransplant model with SK-Mel-147-mCherry cells.
When do mice in the Tyr:CreERT2;BRAFV600E;Ptenflox/flox model usually show lymphatic melanoma metastases? Do they even develop distant melanoma metastases at a certain time point in these models?
Please stain for melanoma cells in the dissected lymph nodes to prove that it is a premetastatic state or to showwhether there are regional lymph node metastases. Moreover, please verify that there are no distant metastases, since this would then not be an adjuvant treatment setting. Suitable markers for immunohistochemistry or immunofluorescence stainings would be MART-1 or TRP-2.
If there have been any distant metastases found, I would recommend repeating the experiment with an earlier timepoint of tumor excision, for example day 14, to really make sure that the melanomas are treated in an adjuvant setting.
- It would be of great interest to investigate MDK levels in the plasma of tumor bearing mice of both mouse models. Can the findings of the in vitro studies be translated to the mouse models presented in Figure2? And if not, what could be the reason?
- The authors discuss and suggest that treatment with BO-110 could help to improve outcomes of immunotherapy or checkpoint inhibition. An in vivo combinatorial approach of anti-PD-L1 + BO-111 and checkpoint inhibition + BO-110 would be very nice.
- The authors used Lyve1 to stain for lymphatic vessels. Please add Prox1 staining as second, highly sensitive marker to confirm the data.
- Please state, why you only used SK-Mel-147 xenografts and no SK-Mel-103 as a second control? I would not demand all in vivo experiments to be repeated with SK-Mel-103, but the authors should clearly explain the choice of the cell line in the corresponding experiments. For Figure 2G, please add MDH mRNA levels in SK-Mel-103, too.

In general, the manuscript is written precisely. There are minor points I would like to point out to further improve its clarity.

Methods:
As indicated in some figure legends, a t-test was performed. Please add this to your methods section.
Did you check for normal distribution of these groups?

Figures:
Fig. 1A: please show pictures of the isotype control for anti-PD-L1 therapy and vehicle controls for Vemurafenib and BO in the suppl. figures.
Fig. 1C + G: details of the scale bar are just stated in the figure legends. Please put the correct scale and unit aside.
Fig 1F: Striking effect of BO therapy! However, the pictures are far too small. Please provide details with higher magnification. Please put a legend with "Lyve1" aside to be able to read the figure even without the figure legends.
Fig. 2 E: Please provide pictures with a higher magnification and an overlay of Lyve1 + Vegfr3.
Fig 2 G + H: Please mind the alignment of the letters.
Fig. 4A: Please adjust the scale of melanoma to be able to directly compare mRNA levels of melanoma and HLEC.
Fig 4E: see commentary Fig 1F

Text:
Please pay attention to a consistent spelling of "anti-PD-L1" instead of "anti-PDL1".
p. 6, l. 6: There are two blanks in front of VEGFR3.
p. 6, l. 15: "hence the concept...": please revise the phrasing of this sentence

p. 6, l. 25: Please precisely state that it is oncogenic BrafV600E
p. 9, l. 13: "we questioned whether mechanistically...": please revise the phrasing of this sentence
p. 11, l. 27: There is a break in between "Ifnar1-/-"
p. 13, l. 5: Please emphasize that Vegfr3 mRNA levels are downregulated by writing "repressing" or "downregulating" instead of "acting on"
p. 13, l. 21: "distant lymphovascular" instead of "distal lymphovascular"
p. 14, l. 22: Please remove the comma after GEMM in the subheading
p. 15, l. 1: Fullstop in front of Vemurafenib. Two separate sentences, please.
p. 15, l. 2: The "A" of "Animals" seems to have a different style.
p. 15: Paragraph "non-invasive imaging": the company is "Perkin Elmer" not "Perkin Helmer"
p. 16, l. 3: "metastases" instead of "metastasis"
Figure legends: I really do not like the wording of the statistical description in all the legends. Please, write a plain sentence instead, like "statistical significance was determined by ANOVA, p="
p. 25, l. 8: "immunofluorescence of Lyve1 (IF, red signal) or Vegfr3 (IF, green signal)" instead of "immunofluorescence (IF, red signal), as well as to immune-detection of Vegfr3 (IF, green signal)"

Thank you very much for this interesting study.

Referee #2 (Comments on Novelty/Model System for Author):

The work by Olmeda et al. expands upon their previous introduction of Vegf3Luc reporter mice (Olmeda D et al. Nature. 2017) to demonstrate the efficacy of a dsRNA nanoplex (BO-110) in preventing melanoma growth and metastasis. Use of the luciferase reporter mouse presents a state-of-the-art system that allows for investigation on activation at (pre-)metastatic niches, and reporter activity correlates well with drug efficacy and survival.

Referee #2 (Remarks for Author):

The manuscript by Olmeda et al. report live imaging of vascular endothelial growth factor receptor 3 (Vegfr3)-driven lymphangiogenesis in immunocompetent and immunodeficient mouse models of tumor growth, and present this as a novel platform for drug screening in vivo. Their previous work introduced MetAlert mice as a novel transgenic line (Olmeda D et al. Nature. 2017), and in this paper, they validate this platform for the spatiotemporal analysis of Vegfr3 reporter activity in autochthonous melanomas and patient-derived xenografts, using double-stranded RNA mimics (dsRNA nanoplexes) to test the inhibition of lymphangiogenesis and tumor metastasis. Their dsRNA polyplex (synthetic (poly)inosinic:polycytidylic acid; BO-110) acts via dual transcription inhibition of midkine (MDK) in melanoma cells and Vegfr3 in lymphatic endothelial cells (LECs). Their thorough analysis demonstrates the efficacy of BO-110 in both established melanomas and progressive disease after surgical excision of primary lesions. The correlation of Vegfr3Luc signal and tumor growth/response is very impressive, however, mechanistically, it is unclear the direct relationship between Vegfr3Luc activity and de novo lymphangiogenesis, and the contribution of this pathological lymphangiogenesis on tumor growth and metastasis, rather than direct effects of BO-110 on tumor cell, or other stromal cell types. Further clarification would improve our understanding of the role of de novo lymphangiogenesis in these processes.

SPECIFIC COMMENTS:
1. The paper presents live imaging of pathogenic de novo lymphangiogenesis, however, it is unclear whether the Vegfr3Luc reporter activity actually correlates with lymphangiogenesis, rather than simply Vegfr3Luc reporter activity related to function. As well, it may be possible that Vegfr3 transcriptional activation may occur in non-LEC cell types. The high level of Luc reporter signal in Vegfr3Luc mice appears to correlate with areas/tissues which do not have a particularly high density of LECs, but may contain other cell types that can express Vegfr3. While their demonstration of the direct effects of BO-110 on LECs, in vitro, the regulatory mechanism described in LECs may also occur in other cell types. Quantitation of LEC density in tumors (peri- and intratumoral) and other metastatic sites would be helpful to demonstrate in vivo de novo lymphangiogenesis. Figs. 1F and 4E demonstrates immunostaining for Lyve1 in lymph nodes, but it is unclear which lymph nodes, and whether there are early differences in tumor lymphatic density (or at other sites of Vegfr3Luc activity). As well, Fig. 1G shows mCherry-SK-Mel-147 cells in the brachial, axillary and sentinel lymph nodes, but it is unclear at what time point these lymph nodes were collected/analyzed. It would be interesting to understand whether early activation of Vegfr3Luc and de novo lymphangiogenesis may precede metastatic seeding. Further, are there differences in lymphatic vessel morphology in addition to increased lymphatic density? Is de novo lymphangiogenesis present in other pre-metastatic niches (i.e. lung, skin, liver, brain)?
1. In their use of αPDL1 and vemurafenib in Vegfr3Luc mice crossed with Tyr:CreERT2;BrafV600E;Ptenflox/flox (immunocompetent) mice, there is an apparent preserved Vegfr3Luc activity at metastatic sites. Is there metastatic growth at these sites? Further, using this immunocompetent model, it would be of interest to see the de novo lymphangiogenesis at the primary tumor site and (pre-)metastatic niches with and without BO-110 treatment. In this setting, how does BO affect immune cell infiltration/trafficking?
2. What are the direct effects of BO-110 on melanoma growth? While Fig. 2D shows BO-110 does not significantly reduce SK-Mel-147 cell viability in vitro, is there an effect on proliferation? Previous work from this group has demonstrated use of BO-110 induces self-killing of melanoma cells (Tormo D et al. Cancer Cell. 2009; Alonso-Curbelo D & Soengas MS. Autophagy. 2010).

While BO-110 reduces MDK mRNA expression in vitro and in vivo, could there be another factor modulating tumor cell growth/function in this study (downstream of type 1 interferon signaling)?
3. For the data related to tumor excision, followed by BO-110 treatment, were there established (micro)metastases? Are the striking survival effects due to combined effects on tumor growth? Karg M et al. (Invest Ophthalmol Vis Sci. 2020) report loss of MDK function reduce melanoma cell proliferation, viability and increase apoptosis, and that MDK promotes tumor cell migration, while their previous work using shRNA directed against MDK did not demonstrate an effect on primary tumor growth.
4. In Fig. 4B, the authors demonstrate VEGFR3 mRNA levels correlated to HLEC tube formation. In the representative image in Fig. 4C, it appears as though BO-110 with IgG control actually disrupts tube formation, as the cells in the BO-110 micrograph appear to be growing as a monolayer rather than tubes (also seen in the micrographs in Fig. 2C and EV Fig. 3A), however, it actually appears there is a greater number of LECs in the field of view, which suggested increased LEC proliferation in BO-110-treated groups, which is at odds with reduced VEGFR3 mRNA. It would be useful to differentiate LEC proliferation from LEC migration/tube formation, and to demonstrate whether downstream VEGFR3 signaling is actually reduced as a result of BO-110. Does BO-110 alter LEC function, in particular immunomodulation?
5. Is there altered type 1 interferon levels in metastatic sites? Does this precede Vegfr3 activation and/or de novo lymphangiogenesis?
6. Lucas ED et al. (J Immunol. 2018) demonstrated that type 1 IFN (along with PDL1) coordinate LEC expansion and contraction during an inflammatory immune response, and it would be of interest to understand whether PDL1 is also modulated by BO-110, considering the efficacy of PDL1 blocking antibodies.
7. After administration of BO-110, where do these dsRNA nanoplexes accumulate? Are they present at metastatic sites? Or could effects be due to systemic IFN/MDK modulation?

Referee #3 (Comments on Novelty/Model System for Author):

The analysis of the lymphatic vasculature as well as the blood vasculature in the VEGFR-LUC/melanoma models is poor and needs to be improved greatly to support the authors' interpretations of their data and model.

Referee #3 (Remarks for Author):

The authors here describe a novel therapy, the dsRNA mimic BO-110 (BO) to inhibit tumor lymphangiogenesis and tumor growth in both genetic and xenograft mouse models.
These studies take advantage of their previously described VEGFR-Luc lymphatic reporter mouse line which they state can be used to look at tumor cell metastasis via live imaging. An extensive number of mouse models and supporting in vitro studies were done to support their studies. However, the paper is hard to follow as written as it is missing critical information and jumps from idea to idea without linking them. In addition, essential analysis and quantification of both the blood a lymphatic vasculature is missing from their studies. As such, the authors often overinterpret the data that is presented. Overall the manuscript is of a topic that is of high interest to a broad audience, but a major revision is necessary that required additional assessments of their model.

Major Comments:

1) In the first paragraph of the Introduction, the authors state, "Rather unexplored from a therapeutic perspective (in melanoma and other tumor types) is the lymphatic system." This is not true as several groups have looked at blocking lymphangiogenesis, VEGFR3 in melanoma models in particular, to prevent metastasis in mouse models. Many of the references describing this work is missing from the paper. Reliance on reviews should be minimized and primary literature cited.

2) A better description of the VEGFR3-Luc mouse. Where are the lymphovascular niches developing in the models? What are these lymph nodes? What are the organs? Does this model also have a signal for tumor angiogenesis as VEGFR3 is also expressed and required for tumor angiogenesis?

3) In Figure 1A, it is unclear what the authors are trying to say. It appears that anti-PD-L1 and vemurafenib were anti-lymphangiogenic in their models as there is less luciferase expressed in the long treatment group. What did lymphangiogenesis look like in the organs with signal? Was the presence of tumor cells assessed?

4) The fact that anti-PD-L1 and vemurafenib were 100% successful at suppressing primary tumor growth is not unexpected as its growth should not be dependent on neo-lymphangiogenesis. Could reduced tumor growth be due to reduced angiogenesis?

5) Figure 1F is not sufficient to show reduced lymphangiogenesis in the lymph node. 1) LYVE1 is not specific to the lymphatic endothelium and is expressed by macrophages and blood endothelial cells. 2) The low magnification of the image makes it impossible to assess cell type and specificity of staining as the images are pixilated when blown up.

6) The interpretation of the data presented in Figure 2C is not accurate. 1) the Matrigel assay is transient and only assesses HLEC migration and proliferation and does not assess tube formation. Thus, it is not a tubulogenesis assay. 2) It appears that

BO induced HLEC proliferation in this assay at the expense of network formation.

7) How was cell viability assessed in Figure 2D? It was not described in the methods and text in figure legend is not sufficient. There are also better more accurate ways to assess cell viability.

8) BO effect on LEC proliferation in vitro and in vivo should be assessed. The Matrigel assays throughout suggest BO promotes LEC proliferation. Moreover, the authors show data that BO downregulates VEGFR3 expression. Thus, the loss of LUC in the mouse model may be due to reduced VEGFR3 expression and not due to reduced lymphangiogenesis. In fact, the authors show that there are LYVE1+ vessels that lose VEGFR3 expression after BO treatment.

9) "compromised sprouting upon BO-110 treatment" is not demonstrated in Figure 2E. Moreover, quantification of lymphatic density would needs to be done to be able to comment on BO's effect on lymphangiogenesis.

10) VEGF C is also expressed by HLECs. Did BO treatment affect VEGF-C transcripts in HLECs? Could increased VEGF-C be the reason for increased HLEC density in the Matrigel assay Figure 2C?

11) What is presented in Figure 3F? The figure legend states it is HLECs treated with BO, while the text states that it was melanoma tumor cells?

12) In Figure 5, expression of Cherry should be presented to show that increased lymphangiogenesis correlated with metastatic growth. As currently presented, the authors cannot comment on growth of metastasis in this model.

Minor Comments:

1) The manuscript (abstract) is hard to follow as it uses a large number of "jargon words" making more a collection of key words rather than presenting an easy to follow story.

2) A description and definition of lymphovascular niche should be provided as it is the basis of their entire study.

3) Description of metastasis is missing when describing VEGFR3-Luc mouse. Where are the lymphovascular niches developing in the models? What are these lymph nodes? What are the organs?

4) The authors state the t the MetAlert model could be used to identify compounds to block tumor growth and lymphangiogenesis. However, they do not use the model as a drug screening method, but as a preclinical model to test specific drugs. Its use as a screening tool would be time consuming and expensive.

5) It is unclear why the authors chose PAMP (BO-110). Was there the rationale besides it

**Olmeda et al. REBUTTAL LETTER**

It is our pleasure to resubmit a substantially revised version of our **EMM-2020-12924** manuscript on live imaging of pathogenic neolymphangiogenesis and the identification of compounds that block this process, here shown for the dsRNA mimic BO-110.

In a tour-de-force collaborative effort, we present 28 new panels in the Primary Figures and 13 additional ones in Expanded View Figures. We also include a Video and two Tables to address experimentally each of the critiques of the Reviewers. These centered primarily on the three main points as indicated by the Editors:

1. "Define therapeutic and adjuvant therapy modalities"
2. "Better analysis of the metastasis and lymphatics in your mouse model over time"
3. "Further investigation into the mechanism of action of BO-110"

Completing these studies involved time (about a year) because the animal facility at our institute is quite strict and was closed for safety reasons for non-essential, non-COVID related experimentation. Breeding had to be reactivated, which required effort for some of the long-term assays here, which include genetically modified mice involving 4 alleles (*Vegfr3$^{Luc}$;TyrCre::ERT2;Braf$^{V600E}$,Pten$^{fl/fl}$*). We are however, glad to provide new data on comprehensive kinetic analyses of different therapeutic modalities in our *MetAlert* mice. We also include new information on the induction (and blockade) of neolymphangiogenesis and its impact on metastasis at different anatomical sites. Moreover, we also carried out proteomic and transcriptomic studies to assess the mode of action of BO-110, followed by validation in tumor and lymphatic endothelial cells. We also present a more detailed explanation of previous publications of our *MetAlert* mice (in *PNAS* and *Nature* as indicated below), where we had addressed many of the technical questions of the Referees on the monitorization of tumor-driven neolymphangiogenesis *in vivo*.

We provide below an **Index** to have all new data at a glance (in **blue and bold** fonts). Figures that have been rearranged to present the results in a more straightforward manner are highlighted in **brown and bold**:

**Figure 1. Identification of anti-lymphangiogenic compounds in *Vegfr3$^{Luc}$* GEMM mice**

**Fig. 1A** Schematic representation of the *Vegfr3$^{Luc}$* GEMM (MetAlert) mice.

**Fig. 1B** Luciferase-based imaging of long term drug response in *Vegfr3$^{Luc}$; Tyr: CreERT2; Braf$^{V600E}$; Pten$^{flox/flox}$* mice.

**Fig. 1D** Histological visualization of lymphatic vessel density in tumors of *Vegfr3$^{Luc}$; Tyr: CreERT2; BrafV600E; Pten$^{flox/flox}$* melanomas treated with vehicle or BO-110.

**Fig. 1E** Quantification of lymphatic vs angiogencic vessel density in tumors and lungs of *Vegfr3$^{Luc}$; Tyr: CreERT2; Braf$^{V600E}$; Pten$^{flox/flox}$* mice treated with vehicle or BO-110.

**Fig. 1F** Luciferase-based imaging of long term drug response to BO-110 in *MetAlert-PDX* models.

**Figure 2. Inhibitory effects of BO-110 on pro-lymphangiogenic factors**

**Fig. 2A** Luciferase-based imaging of short term drug response in *Vegfr3$^{Luc}$; Tyr: CreERT2; Braf$^{V600E}$; Pten$^{flox/flox}$* immunocompetent mice.

**Fig. 2B** Response to BO-110 of melanoma xenografts implanted in *Vegfr3$^{Luc}$* nu/nu lymphoreporter.

**Fig. 2E** Reporter assays in HLEC to define the impact of BO-110 on the *Flt4 (*VEGFR3*)*-promoter.

**Fig. 2F** Time-lapse imaging of the tubulogenic activity of HLECs in the presence of BO-110 (see **Extended View Video 1** for additional detail).

**Fig. 2G** Lack of cell death in HLEC cells treated with BO-110.

**Figure 3. Inhibitory effects of BO-110 on *MDK***

**Fig. 3A** Inhibitory effect of BO-110 on *MDK* mRNA expression on human melanoma cells (SK-Mel-147). See **Fig EV2A** for four additional melanoma cell lines of different genetic background.

**Fig. 3B** *MDA5* induction by BO-110 in SK-Mel-147. See **Fig EV2B** for additional cell lines.

**Fig. 3C** Quantification of cell death in SK-Mel-147 treated with BO-110. See **Fig EV2C** for additional cell lines.
**Fig. 3D** Reporter assays to define the inhibitory effect of BO-110 on the *MDK*-promoter.

**Figure 4. –Omic analyses for genome-wide characterization of the mode of action of BO-110 in melanoma cells and LECs**

**Fig. 4D** Heatmaps comparing GSEA Normalized Enrichment Score of the indicated REACTOME genesets in melanoma SK-Mel-147 and HLEC. See **Fig. EV4B-D** for additional information on signaling cascades altered in these cells, and **Table II** for additional detail.
**Fig. 4E** Heatmaps depicting immune-related genes found differentially expressed by RNAseq in BO-110 treated SK-Mel-147 and LECs.

**Figure 5. IFN-based repressive activity of BO-110 on melanoma-induced neo-lymphangiogenesis**

**Fig. 5E** Histological analyses of lymphatic vessel density in lymph nodes of *Ifnar1 wt, het and KO mice* bearing MDK-expressing tumors and treated with or without BO-110.

**Figure 6. BO-110 induces a systemic interferon response that reduces MDK blood levels and tumor-driven lymphangiogenesis**

**Fig. 6A-H** Time course analyses to the define the impact of BO-110 on the response of tumor-bearing mice (B16R2L implants).

- **Fig. 6A** BO-110 on tumor size.
- **Fig. 6B** BO-110 on Vegfr3-dependent luciferase (Vegfr3-Luc) emission at the tumor area.
- **Fig. 6C** BO-110 on Vegfr3-Luc emission at lymph nodes.
- **Fig. 6D** Vegfr3-Luc emission in the spleen.
- **Fig. 6E** Vegfr3-Luc emission in the liver.
- **Fig. 6F** Vegfr3-Luc emission in the lung.
- **Fig. 6G** Inhibitory effect of BO-110 on circulating MDK.
- **Fig. 6H** Activating effect of BO-110 on circulating IFN-$\beta$.

**Fig. 6I-P** as for **Fig. 6A-H**, but on mice implanted with SK-Mel-147.

**Figure 7. BO-110 prevents metastatic melanoma relapse after surgery**

**Fig. 7B** Metastatic relapse after surgery visualized by fluorescence imaging in Vegfr3$^{Luc}$ mice implanted with mCherry SK-Mel-147.

**Figure EV1. Identification of BO-110 as an antilymphangiogenic agent in *MetAlert* mice, with controls for selective inhibition of *Vegfr3$^{Luc}$* signaling**

**Fig. EV1C** Effect of BO-110 on the lymphatic vessel density (Prox1 and Lyve1 staining) in lungs of *Vegfr3$^{Luc}$;Tyr: CreERT2; Braf$^{V600E}$; Pten$^{flox/flox}$* –bearing melanomas.
**Fig. EV1D** Lymphatic vessel density in lymph nodes of Vegfr3$^{Luc}$ nu/nu mice bearing human xenoimplants and treated with BO-110.
**Fig. EV1E** Differential effect of BO-110 in lymphatic vs blood vessel density in tumor-activated lymph nodes of *Vegfr3$^{Luc}$* nu/nu mice.
**Fig. E1VG** Quantificacion of Vegfr3-Luc emission by BO-110, not recapitulated by expression of Luc by an unrelated SV40 promoter

**Figure EV2. *MDA5* induction and *MDK* repression by BO-110 in a panel of melanoma cell lines**

**Fig. EV2A** Inhibitory effect of BO-110 on *MDK* mRNA expression in four independent melanoma cell lines. Data Complementing **Fig 3A**.
**Fig. EV2B** Induction of *MDA5* by BO-110 in the indicated melanoma cell lines. See also **Fig 3B**.
**Fig. EV2C** Survival of the indicated melanoma cells in the conditions assayed for the anti-lymphangiogenic activity of BO-110. See also **Fig 3D**.

**Figure EV3. -Omic analyses of the BO-110 mechanism of action**

**Fig. EV3B** Results from RNAseq in SK-Mel-147 melanoma cells vs HLEC upon BO-110 treatment. Shown are overlapping and differentially regulated pathways as defined by GSEA through the Hallmarks dataset. See also **Appendix Table II** for the complete gene set list.

**Fig. EV3C** Venn diagrams depicting BO-110-induced changes in the transcriptome of SK-Mel-147 melanoma cells vs HLEC. Shown are overlapping and differentially regulated pathways as defined by GSEA through the REACTOME dataset.

**Fig. EV3D** Representative enrichment plots for IFN and dsRNA-sensor associated gene sets identified in BO-110 treated SK-Mel-147 or HLEC.

See results for proteomic data in SK-Mel-147 and SK-Mel-28 in **Appendix Table I.**

**Figure EV4. IFN-dependent inhibition of lymphangiogenesis by BO-110**

**Fig. EV4C** Histological visualization of the impact of BO-11O on the Lymphatic vessel density (Lyve1 and prox1 dual staining) of lymph nodes of *Ifnar1*$^{+/+}$, *Ifnar1*$^{+/-}$ and *Ifnar1*$^{-/+}$ mice bearing MDK expressing tumors (B16-F10). Higher magnifications are shown at **Fig. 5E**.

**Fig. EV4D** Quantification of Lyve1/Prox1 staining in lymph nodes of *Ifnar1*$^{+/+}$, *Ifnar1*$^{+/-}$, *Ifnar1*$^{-/+}$ mice of Fig. EV4C.

**Figure EV5. Disseminated tumor cells at LN and lung lymphovascular metastatic niches**

**Fig. EV5** Histological visualization of neolymphangiogenesis (Vegfr3 staining, green) and micrometastastes (mCherry SK-Mel-147, red), 4 weeks after tumor cell implantation and before tumor excision.

Appendix Video 1. **Live imaging of the tubulogenic activity of HLEC cells in the absence or presence of BO-110**

Appendix Table I. **Proteomic analyses of the impact of BO-110 in SK-Mel-147 and SK-Mel-28**

Appendix Table II. **Comparative effect of BO-110 in SK-Mel-147 vs HLEC defined by RNAseq**

## REPLY TO REFEREES

Questions of the Referees are herein numbered for a more clear Point-by-point answer to their critiques.

**Referee #1**

We are glad that the Referee considers that "The 'MetAlert' mouse model is a very elegant approach to visualize and monitor lymphatic premetastatic niche formation" and that "this study is well designed and of great novelty for the field of melanoma research". We hope that the large amount of new data provided in the manuscript addresses his/her concerns:

1.1. The discrimination between the therapeutic and adjuvant therapy modality needs to be precisely presented. Your previous publication (Olmeda et al., 2017, Nature - Figure 2) indicates that mice start to develop LN metastases at around 2 weeks and distant metastases at around 4 weeks in the xenotransplant model with SK-Mel-147-mCherry cells. When do mice in the *Tyr:CreERT2;BRAFV600E;Ptenflox/flox* model usually show lymphatic melanoma metastases? Do they even develop distant melanoma metastases at a certain time point in these models?

We apologize for the oversight of not having described in sufficient detail the experimental conditions tested in our *MetAlert VEGFR3*$^{Luc}$ models. The Methods Section has been rewritten to clarify that the starting point for the different treatments in this manuscript follow comprehensive kinetic analyses of tumor dissemination we had reported in Olmeda et al. *Nature* 2017[1]. In that study, we had combined non-invasive imaging with histological and qPCR-based detection of tumor cells, to demonstrate the "MetALert" concept, namely, induction of bioluminescence preceding lymph node and visceral metastasis. Following these and additional indications of the reviewer (see Point 1.2), we now include additional histological analyses of lymph nodes (**Fig. EV5**) as a reference for adjuvant treatments. Moreover, to further ease the reading, figure panels corresponding to all time courses *in vivo* include now arrows marking the starting point of the drug administrations.

In addition, we emphasize more clearly now that while some analyses were performed on advanced full blown tumors (e.g. Fig. 1C, 1G, 1H; Fig. EV1A; Fig. EV1F,G; **Fig. 1B;** new **Fig. 1D,E; Fig. EV1E,** see below), our prime interest was on earlier time points. Specifically, we set to recapitulate adjuvant settings to follow metastatic dissemination after surgical excision of primary lesions (Fig. 7A,C, **Fig. 7B**, new **Fig. EV5**, see Point 1.3).

With respect to the tamoxifen-inducible *Tyr::CreERT2; Braf*$^{V600E}$*;Pten*$^{flox/flox}$, these animals are indeed known in the field for their poorly metastatic behavior. Nevertheless, these mice can still be used for functional and pharmacological analyses of drug response in metastatic settings, as we have previously reported[2]. However, even localized topical administration of tamoxifen induces various primary melanomas in this strain. Therefore, we opted to use xenograft models to define metastatic relapse after surgery with a double consideration: (i) a more homogeneous tumor growth, and (ii) a more controlled excision of single tumors, to monitorize the reactivation of neolymphangiogenesis and metastatic growth at distal sites. Fig. 7 has been reorganized to visualize in parallel bioluminescence (Fig. 7A) and metastatic growth (**Fig. 7B**) prior and post treatment.

1.2. Please stain for melanoma cells in the dissected lymph nodes to prove that it is a premetastatic state or to show whether there are regional lymph node metastases.

This information is now included in **Fig. EV5.** Specifically, we show histological analyses of lymph nodes from Vegfr3$^{Luc}$ mice either at basal conditions (non-tumor bearing) or 4 weeks after subcutaneous implantations of mCherry-labeled SK-Mel-147 melanoma cells, preceding the surgical procedures to assess relapse after surgery. Shown are immunofluorescence images corresponding to dual staining for VEGFR3 (to detect tumor induced-lymphangiogenesis) and for Cherry (to detect the melanoma cells).

1.3. Moreover, please verify that there are no distant metastases, since this would then not be an adjuvant treatment setting. Suitable markers for immunohistochemistry or immunofluorescence stainings would be MART-1 or TRP-2. If there have been any distant metastases found, I would recommend repeating the experiment with an earlier timepoint of tumor excision, for example day 14, to really make sure that the melanomas are treated in an adjuvant setting.

Adjuvancy was considered in this manuscript to recapitulate a key clinical situation of metastatic growth in stage III patients[3].

We would like to emphasize that here we took advantage of our previous Olmeda et al. *Nature* 2017, were relapse after surgery in the *Vegfr3$^{Luc}$-MetAlert* mice was comprehensively analyzed in the absence of treatment. This was performed by visualizing tumor cells at different sites by histology and quantifying disseminated cells by qRT-PCR. This study revealed time points where removal of the cutaneous lesions nearly abrogated systemic neo-lymphangiogenesis, and that reactivation of luciferase emission invariably preceded distant metastasis (at lymph nodes, lung or skin; see **Fig. Reviewers 1** below). We refer better to these analyses now and emphasize that the objective of this new manuscript is to monitor this reactivation of bioluminescence (marked with a dashed red line in ***Fig. R1***), as a platform to identify compounds that prevent metastatic relapse, as the reviewer indicated. Fig. 7 was reassembled to demonstrate the viability of this approach, and to show more directly, the potent ability of BO-110 to prevent neo-lymphangiogenesis (Fig. 7A), blunt metastasis (here visualized by fluorescence imaging *in vivo*; **Fig. 7B**) and allow for a significantly extended overall survival (Fig. 7C).

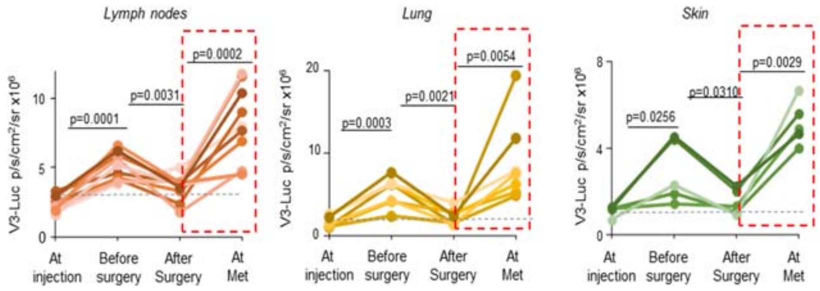


***Figure R1.*** **Imaging metastatic relapse after surgery in *Vegfr3$^{Luc}$*-MetAlert mice reveal.** Emission of Vegfr3-associated luciferase induced by SK-Mel-147-mCherry prior and after surgical resection of the cutaneous lesions. LN (n=9), lung metastases (n=7) and skin metastases (n=5). t-Test. Data from Olmeda et al. *Nature* 2017. The red dotted squares highlight the induction of bioluminescence preceding relapse we aimed to block pharmacologically in this new paper.

1.4. It would be of great interest to investigate MDK levels in the plasma of tumor bearing mice of both mouse models. Can the findings of the in vitro studies be translated to the mouse models presented in Figure2? And if not, what could be the reason?

We have now analyzed the inhibitory effect of BO-110 on secreted MDK measured in blood. This was assessed in two different model systems: in the context of xenografts by murine B16R2L (**Fig. 6G**) and by the human SK-Mel-147 (**Fig. 6O**). Therefore, *in vitro* studies the reviewer mentions (current Fig. 3A,E), can thus be translated *in vivo* (this also strengthened by the abrogation of MDK secretion at the primary tumors shown histologically in Fig. 3F).

As the repression of MDK expression by BO-110 is a central point in this paper, we considered important to further measure MDK in blood at different time points before and after treatment, with a complete analysis of the impact of this compound on tumor growth (**Fig. 6A, Fig. 6I**). We also quantified the inhibition of neolymphangiogenesis at the cutaneous lesions (**Fig. 6B, Fig. 6J**), and distally, in lymph nodes (**Fig. 6C, Fig 7K**), spleen (**Fig. 6D, Fig. 6L**), liver (**Fig. 6E, Fig. 6M**) and lung (**Fig. 6G, Fig. 6O**). Importantly, these inhibitory effects of BO-110 were also associated with a large secretion of IFN-$\beta$, which we also monitored in blood (**Fig. 6H, Fig. 6P**).

We thank the reviewer for having prompted these analyses in **Fig. 6**, as they provide a more mechanistic insight on new actions of BO-110 that could be monitored in liquid biopsies.

1.5. The authors discuss and suggest that treatment with BO-110 could help to improve outcomes of immunotherapy or checkpoint inhibition. An in vivo combinatorial approach of anti-PD-L1 + BO-110 and checkpoint inhibition + BO-110 would be very nice.

We have data that support in fact that BO-110 synergizes with anti-PD-L1 treatment in animal models ***(Fig. R2***). However, we consider that this drug combination is beyond the scope of this paper, as it departs from the effects of this compound on MDK and the lymphatic vasculature.

Figure for referees removed

1.6. The authors used Lyve1 to stain for lymphatic vessels. Please add Prox1 staining as second, highly sensitive marker to confirm the data.

We have now include representative examples of double Lyve1/Prox1 stainings in the absence and presence of BO-110 in right panels of **Fig. EV1C** (lung) and **Fig. EV1D** (lymp nodes). Pseudocoloring showing the overlap between these two markers is shown histologically in **Fig. 1D** (tumor sites) and in the left panels of **Fig. EV1C** (lung) and **Fig. EV1D** (lymph nodes). The quantification of this Lyve1/Prox1 overlap to illustrate the anti-lymphangiogenic effect of BO-110 in tumor and lung is shown in **Fig. 1E** and in lymph nodes in **Fig. EV1E**. To assess for specificity of BO-110 in the vasculature, blood endothelial cells were also quantified in the presence and absence of this compound also at different anatomical sites (**Fig. 1E, Fig. EV1E**).

The stainings above correspond to data in wild-type mice. Dual staining for Prox1 and Lyve1 was also performed in mice with homozygous and heterozygous losses of Ifnar1 (see immunohistochemistry in **Fig. 5E** and **Fig. EV4C**, with quantifications in **Fig. EV4D**).

1.7. Please state, why you only used SK-Mel-147 xenografts and no SK-Mel-103 as a second control? I would not demand all in vivo experiments to be repeated with SK-Mel-103, but the authors should clearly explain the choice of the cell line in the corresponding experiments. For Figure 2G, please add MDH mRNA levels in SK-Mel-103, too.

We appreciate this point on additional controls of the effect of BO-110 in different cell lines. For xenografts, we thought it more informative to reproduce data generated for SK-Mel-147 in immune-deficient mice, with independent cells that could be tested in immune-competent settings. A new entire figure is now devoted to BO-110 on tumor growth, neolymphangiogenesis at different sites, and circulating levels of MDK and IFN-β in the context of SK-Mel-147 (**Fig. 6I-P**) and B16R2L (**Fig. 6A-H**).

Regarding data in Fig 2G, now Fig. 3A (SK-Mel-147), the acute repression of BO-110 on *MDK* mRNA is now shown in additional cell types (**Fig. EV2A**): SK-Mel-103 (both NRAS mutated), as well as in 451LU and WM902B (Braft mutated) and in SK-Mel-28 (p53 mutant). For a more complete analysis, we have also performed an analysis of the induction of the main BO-110 sensor MDA-5 (**Fig. EV2B**) before the induction of cell death (**Fig. EV2C**).

1.8. In general, the manuscript is written precisely. There are minor points I would like to point out to further improve its clarity.

Methods:
As indicated in some figure legends, a t-test was performed. Please add this to your methods section.
Did you check for normal distribution of these groups?
Information about the specific statistical procedures used to define significance has been added to the corresponding Figure Legends and Methods section. In general, data was analyzed with one/two-way Anova and Mann-Whitney tests. In those cases where a parametric t-test was used, normal distribution was verified with a Shapiro-Wilk test.

Figures:
Fig. 1A: please show pictures of the isotype control for anti-PD-L1 therapy and vehicle controls for Vemurafenib and BO in the suppl. figures.
The isotype control pictures for anti-PD-L1 have been added to panels now in **Fig. 1B**, **Fig. 2A**. Vehicle controls for BO-110 and Vem were not shown for simplicity (as they are equivalent to images for the "Induced"-non treated counterparts), but were included in the quantifications of Fig. 1C. All other figure panels for the response to BO-110 in vivo have the vehicle controls (Fig. 1FG-H, **Fig. 2B**, Fig. 3F, Fig. 5D, **Fig. 5E**, **Fig 6A-P,** Fig. 7A-C, Fig. EV1A, Fig. EV1F, **Fig. EV4C, D**).

Fig. 1C + G: details of the scale bar are just stated in the figure legends. Please put the correct scale and unit aside.
All figure panels with histological images include scale bars with the corresponding units.

Fig 1F: Striking effect of BO therapy! However, the pictures are far too small. Please provide details with higher magnification. Please put a legend with "Lyve1" aside to be able to read the figure even without the figure legends.
We have included double Lyve1/Prox stainings (and the corresponding labeling) for histological analyses in this figure panel, now Fig. 1D. We have added high magnifications and pseudocloring of this dual staining for lymphatic vessels in **Fig. EV1C,D**. We also include the full source data as Supplementary information.

Fig. 2 E: Please provide pictures with a higher magnification and an overlay of Lyve1 + Vegfr3.
We have added the overlay as requested in Fig. EV1B

Fig 2 G + H: Please mind the alignment of the letters.
Corrected, now in all panels.

Fig. 4A: Please adjust the scale of melanoma to be able to directly compare mRNA levels of melanoma and HLEC.
Corrected, now in Fig. 5A.

Fig 4E: see commentary Fig 1F
We have included new double stainings for Lyve1/Prox1 at a larger magnification in **Fig. 5E**, with larger areas of lymph nodes shown in **Fig. EV4C** and the corresponding quantification in **Fig. EV4D**.

Text:
Please pay attention to a consistent spelling of "anti-PD-L1" instead of "anti-PDL1".
p. 6, l. 6: There are two blanks in front of VEGFR3.
p. 6, l. 15: "hence the concept...": please revise the phrasing of this sentence
p. 6, l. 25: Please precisely state that it is oncogenic BrafV600E
p. 9, l. 13: "we questioned whether mechanistically...": please revise the phrasing of this sentence

p. 11, l. 27: There is a break in between "Ifnar1-/-"
p. 13, l. 5: Please emphasize that Vegfr3 mRNA levels are downregulated by writing "repressing" or "downregulating" instead of "acting on"
p. 13, l. 21: "distant lymphovascular" instead of "distal lymphovascular"
p. 14, l. 22: Please remove the comma after GEMM in the subheading
p. 15, l. 1: Fullstop in front of Vemurafenib. Two separate sentences, please.
p. 15, l. 2: The "A" of "Animals" seems to have a different style.
p. 15: Paragraph "non-invasive imaging": the company is "Perkin Elmer" not "Perkin Helmer"
p. 16, l. 3: "metastases" instead of "metastasis"

The text was proofread to ensure proper grammar and avoid typos.

Figure legends: I really do not like the wording of the statistical description in all the legends. Please, write a plain sentence instead, like "statistical significance was determined by ANOVA, p="
We had to simplify the legends as figures are heavy in panels, but tried to indicate the statistical tests performed in a more clear manner.

p. 25, l. 8: "immunofluorescence of Lyve1 (IF, red signal) or Vegfr3 (IF, green signal)" instead of "immunofluorescence (IF, red signal), as well as to immune-detection of Vegfr3 (IF, green signal)"
The text has been corrected accordingly.

**Referee #2**

We thank the Referee for his/her remarks on " Their thorough analysis demonstrates the efficacy of BO-110 in both established melanomas and progressive disease after surgical excision of primary lesions. The correlation of Vegfr3Luc signal and tumor growth/response is very impressive". We hope that the answers below clarify the concerns of this Referee on relationship between Vegfr3-Luc activity and de novo lymphangiogenesis.

SPECIFIC COMMENTS:
2.1. The paper presents live imaging of pathogenic de novo lymphangiogenesis, however, it is unclear whether the Vegfr3Luc reporter activity actually correlates with lymphangiogenesis, rather than simply Vegfr3Luc reporter activity related to function. As well, it may be possible that Vegfr3 transcriptional activation may occur in non-LEC cell types. The high level of Luc reporter signal in Vegfr3Luc mice appears to correlate with areas/tissues which do not have a particularly high density of LECs, but may contain other cell types that can express Vegfr3. While their demonstration of the direct effects of BO-110 on LECs, in vitro, the regulatory mechanism described in LECs may also occur in other cell types. Quantitation of LEC density in tumors (peri- and intratumoral) and other metastatic sites would be helpful to demonstrate in vivo de novo lymphangiogenesis.
We apologize for the oversight of not having provided enough information on the demonstration of our Vegfr3$^{Luc}$ mice as lymphoreporters that visualize pre-metastatic niches. The direct correlation between Vegfr3-Luciferase signaling and the neolymphangiogenesis was shown in Martinez-Corral et al. *PNAS*, 2012[4] and Olmeda D et al. *Nature*, 2017[1]. These studies raised great attention in the field, precisely because they allowed for a non-invasive imaging of the expansion of the lymphatic vasculature at time points and at sites that otherwise would not be detectable with standard imaging techniques (see comments in Refs[5,6,7]).

We would like to mention that in Martinez-Corral et al. *PNAS*, 2012 and Olmeda et al. *Nature*, 2017, we presented evidence that although indeed VEGFR3 can be expressed in different cell types (i.e. tip cells in blood vessels or macrophages), this expression is considerably lower than in lymphatic endothelial cells. Moreover, this tumor-driven activation of lymphangiogenesis was demonstrated to be associated with

Vegfr3 induction (see cutaneous melanoma lesions in ***Fig. R3A***) in areas where MDK accumulate (see lungs and lung sections in ***Fig. R3B***).

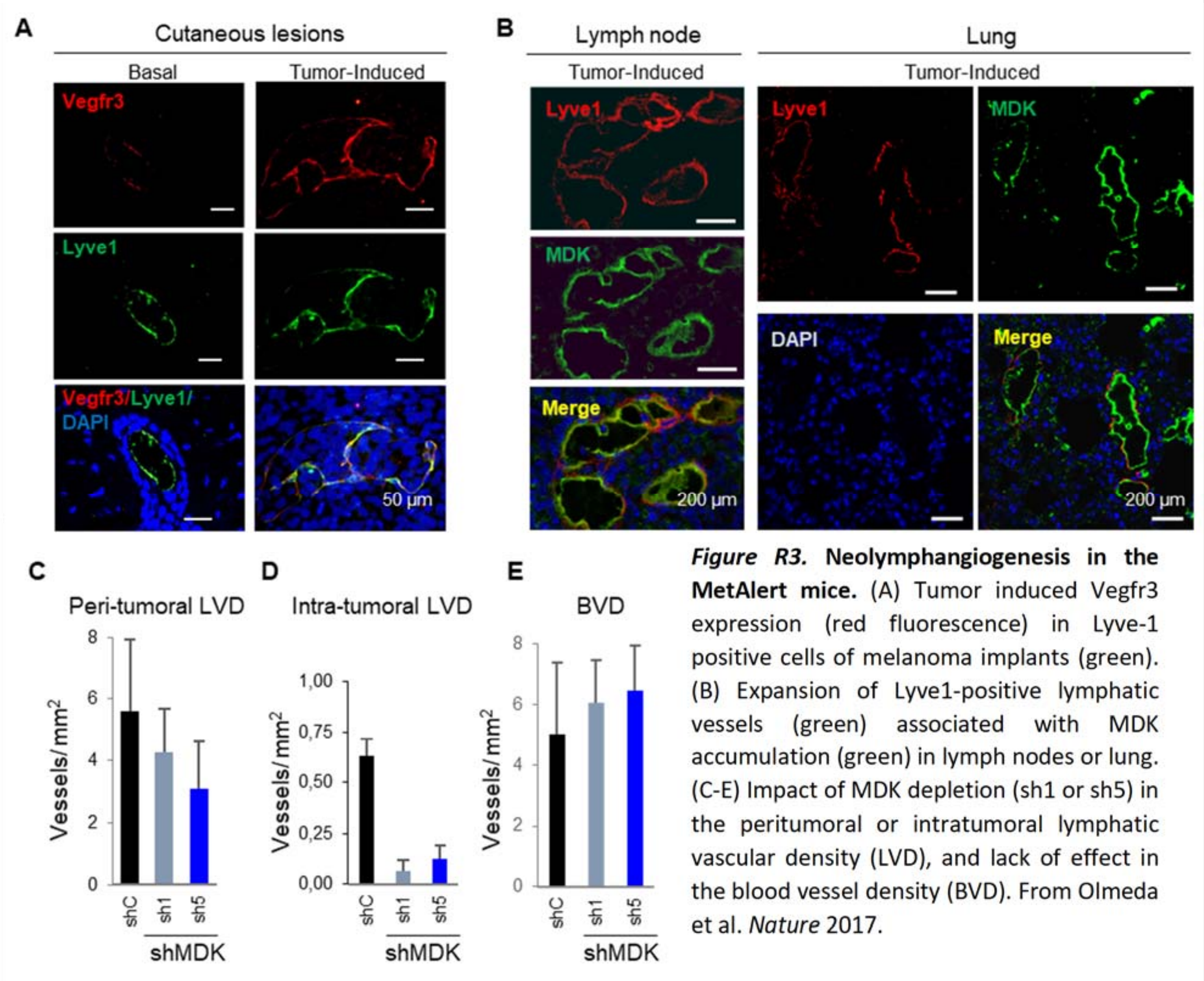


***Figure R3.*** **Neolymphangiogenesis in the MetAlert mice.** (A) Tumor induced Vegfr3 expression (red fluorescence) in Lyve-1 positive cells of melanoma implants (green). (B) Expansion of Lyve1-positive lymphatic vessels (green) associated with MDK accumulation (green) in lymph nodes or lung. (C-E) Impact of MDK depletion (sh1 or sh5) in the peritumoral or intratumoral lymphatic vascular density (LVD), and lack of effect in the blood vessel density (BVD). From Olmeda et al. *Nature* 2017.

The peri-tumoral and intratumoral lymphatic vascular density were also studied and found to depend on MDK, as defined by depletion with two independent shRNA constructs (see ***Fig. R3C,D***). Of note, MDK shRNAs did not affect the blood tumoral density (***Fig. R3E***). Having published these data, this EMM manuscript focuses now on how to use the *MetAlert* mice, and the associated Vegfr3-linked bioluminescence, to screen for new compounds. This concept is now better emphasized in the Introduction.

2.2. Figs. 1F and 4E demonstrates immunostaining for Lyve1 in lymph nodes, but it is unclear which lymph nodes, and whether there are early differences in tumor lymphatic density (or at other sites of Vegfr3Luc activity).

The lymph nodes in previous Figs 1F and 4E indeed corresponded to sentinel lymph nodes. To provide more insight on the effect of BO-110 on the lymphatic vasculature, and also in response to Point 1.6 of Referee #1, we have performed new histological analyses, showing larger magnifications of Lyve1/Prox1 staining in sentinel lymph nodes (**Fig. EV1D**), lung (**Fig. EV1C**) and tumor sites (**Fig. 1D**).

Quantification of the inhibitory effect of BO-110 on the lymphatic vessel density (and on the angiogenic blood vasculature) is shown at different anatomical sites in **Fig. 1E**, **Fig. EV1E**. We have also provided additional information for the original Fig. 4E (response to BO-110 in *Ifnar1 +/+, +/-* and *-/-* mice) in **Fig. 5E and Fig. EV4C,D**. Quantifications of early differences (24 h after treatment) Vegfr3-Luc at the implantation sites vs distal sites are shown in new **Fig. EV1G** (note also that this fig shows no effect in a model where luciferase is driven not by VEGFR3, but by an unrelated promoter).

2.3. As well, Fig. 1G shows mCherry-SK-Mel-147 cells in the brachial, axillary and sentinel lymph nodes, but it is unclear at what time point these lymph nodes were collected/analyzed

This figure panel (now Fig. 1H) corresponds to lymph nodes collected at the endpoint of the experiment, to visualize the extent of the effect of BO-110 at proximal and distal metastases.

Regarding time-dependent quantifications of neolymphangiogenesis, Olmeda et al. *Nature,* 2017, showed a detailed kinetic analyses of tumor-induced Vegfr3-Luc at multiple organs in untreated mice. Here we have now added time-dependent analyses of Vegfr3-Luc signal at tumor, lymph nodes, spleen, liver and lung in the context of BO-110 effects on xenografts driven by B16R2L (**Fig. 6B-F**) and SK-Mel-147 (**Fig. 6I-N**).

2.4. It would be interesting to understand whether early activation of Vegfr3Luc and de novo lymphangiogenesis may precede metastatic seeding. Further, are there differences in lymphatic vessel morphology in addition to increased lymphatic density? Is de novo lymphangiogenesis present in other pre-metastatic niches (i.e. lung, skin, liver, brain)?

As mentioned before, Olmeda et al *Nature*, 2017 demonstrated that systemic neolymphangiogenesis and Vegfr3-Luc emission precede metastatic seeding (see also ***Fig. R1*** in the context of metastatic relapse after surgery).

Regarding the lymphatic vessel density and morphology, this was also shown during development (Martinez-Corral et al *PNAS*, 2012), and in response to tumor induction (Olmeda et al *Nature,* 2017; see for example the left and right panels of ***Fig. R3A*** for cutaneous lesions and in ***Fig. R3B*** in lymph nodes and lungs).

2.5. In their use of αPDL1 and vemurafenib in Vegfr3Luc mice crossed with Tyr:CreERT2;BrafV600E;Ptenflox/flox (immunocompetent) mice, there is an apparent preserved Vegfr3Luc activity at metastatic sites. Is there metastatic growth at these sites?

The signal detected in response to αPD-L1 and vemurafenib corresponds to residual lymphangiogenesis after treatment. At the time points analyzed, metastases were not yet induced in these animals (IgG controls had to be euthanized for humane reasons). The text describes better the main objectives of this experiment (now in **Fig. 1B**): (i) Demonstrate that Vegfr3-Luc emission in these mouse models can be used as a platform to assess drug response in vivo, (ii) show that αPD-L1 and vemurafenib treatments in these animals recapitulate partial responses observed in the clinic and (iii) identify more potent anti-cancer agents (in this case, BO-110).

2.6. Further, using this immunocompetent model, it would be of interest to see the de novo lymphangiogenesis at the primary tumor site and (pre-)metastatic niches with and without BO-110 treatment.

The effect of BO-110 on local and systemic neolymphangiogenesis in this immunocompetent model is shown now separated in mid-term and short-term responses (**Fig. 1B** and **Fig. 2A**, respectively). Histological quantifications of lymphatic (and angiogenic) vasculature at primary tumors is shown in **Fig. 1E** (left panels), and for premetastatic sites at the lung and lymph nodes in **Fig. 1E** (right panels) and **Fig. EV1E**, respectively.

2.7. In this setting, how does BO affect immune cell infiltration/trafficking?

We have performed genome-wide proteomic and transcriptomic analyses that support a role of BO-110 in immune modulation via melanoma cells, and importantly, also by lymphatic endothelial cells that are now shown in **Fig. 4D,E**; **Fig. EV3B-D** (see below). These data include a variety of dsRNA stress response factors and IFN-associated effectors, which we consider that provide mechanistic insight to the previous version of the manuscript, where we linked these processes to the inhibition of MDK and VEGFR3 as a new and unexpected mechanism of action of BO-110.

We have also found that sustained treatment with BO-110, as the case of the clinical derivative BO-112[8,9] does indeed increase the mobilization of various immune cell types which can be detected in blood of tumor-bearing mice (***Fig. R4***). We also have evidence that this effect on immune cells (particularly on CD8$^+$ T cells) may underlie the synergistic effect of BO-110 on the response of checkpoint blockade ($\alpha$PD-L1 treatment), as mentioned above for Referee #1 (***Fig. R2***). However, we consider that these data depart from the main concept put forward on this paper, namely, the *MetAlert* mice as a platform for antitumoral agents that have an acute effect on the lymphatic vasculature. We hope the Referee agrees that immune modulation would be a subject for future studies on BO-110.

Figure for referees removed

2.8. What are the direct effects of BO-110 on melanoma growth? While Fig. 2D shows BO-110 does not significantly reduce SK-Mel-147 cell viability in vitro, is there an effect on proliferation? Previous work from this group has demonstrated use of BO-110 induces self-killing of melanoma cells (Tormo D et al. Cancer Cell. 2009; Alonso-Curbelo D & Soengas MS. Autophagy. 2010). While BO-110 reduces MDK mRNA expression in vitro and in vivo, could there be another factor modulating tumor cell growth/function in this study (downstream of type 1 interferon signaling)?

The text has been rewritten and more data was added (**Fig. 3A-D; Fig. 4D,E**) to highlight new mechanisms of action of this compound on tumor-induced lymphangiogenesis, which had not been anticipated in Tormo D et al. *Cancer Cell*, 2009[10]. We had indeed shown that BO-110 blocks melanoma growth *in vitro* and *in vivo*. Indeed, we selected BO-110 for this current study, because we had found that this compound was a potent inducer of tumor cell autophagy and apoptosis, as the Referee indicates. Here we wanted to explore the *MetAlert* mice to interrogate possible additional effects of this compound, and particularly for whole-body imaging in vivo. This was because as for the case for other dsRNA mimics in clinical trials[8,11-14] , there is no biomarker to gauge early responses to this compound in patients.

What was rather unexpected, was that using the *MetAlert* mice we could uncover an acute and nearly complete blockade of melanoma-driven neolymphangiogenesis just 24 h after one single administration of BO-110, before tumor size was affected (**Fig. 2A,B**). Finding that BO-110 represses *MDK* mRNA expression (Fig. 3A) and secretion (Fig. 3E) so acutely, may have therefore translational implications as an indicator to monitor early clinical responses. These conclusions are now strengthened by demonstrating inhibition of *MDK* mRNA expression in 5 independent melanoma cell lines (**Fig. 3A, Fig. EV2A**), in all cases occurring before the detection of cell death (**Fig. 3C, Fig. EV2C**), as mentioned above for Referee #1. Furthermore, we also included new reporter analyses to demonstrate this effect of BO-110 on *MDK* mRNA indeed occurred by repressor activities at the promoter of this gene (**Fig. 3D**).

We also provide new genome-wide transcriptomic analyses (RNA sequencing) in melanoma cell lines and on lymphatic endothelial cells (**Table II**) so readers can have information of the broad impact of BO-110 on these cell types. As mentioned above in point 2.7, here we focused on IFN signaling (**Fig. 4D,E; Fig. EV3B-D**) as a new blocker of both *MDK* mRNA expression in melanoma cells and on *VEGFR3* in lymphatic endothelial cells.

The Discussion was also rewritten to avoid unintentional misinterpretations regarding the antitumoral effect of BO-110 being solely dependent on the blockade of neolymphangiogenesis. This was never our intention. In fact, we believe that this multi-level action of BO-110 (in tumor cell death, lymphangiognenesis

and IFN-driven signaling) may represent an advantage over other compounds (i.e. Vemurafenib or $\alpha$PD-L1 antibodies) which were found less potent in our *MetAlert* system.

2.9. For the data related to tumor excision, followed by BO-110 treatment, were there established (micro)metastases?

Yes, they were. To clarify this point, we have added images of lymph nodes in mice implanted with of SK-Mel-147 melanoma cells at the time of surgery (**Fig. EV5**). As indicated to Referee #1, time points were selected to treat animals already with micrometastases, as if this would not be the case, there could not possibly be a metastatic relapse after surgery. Adjuvancy was here considered to recapitulate a key clinical situation of metastatic growth after surgical excision of the primary tumor.

2.10. Are the striking survival effects due to combined effects on tumor growth? Karg M et al. (Invest Ophthalmol Vis Sci. 2020) report loss of MDK function reduce melanoma cell proliferation, viability and increase apoptosis, and that MDK promotes tumor cell migration, while their previous work using shRNA directed against MDK did not demonstrate an effect on primary tumor growth.

As mentioned above, indeed the effects of BO-110 need to be considered in the context of apoptosis, autophagy, neo-lymphangiogenesis and IFN-dependent signaling. Regarding MDK, in Olmeda et al. Nature, 2017, we reported intravital microscopy imaging in lymph nodes demonstrating that this protein is indeed required for tumor cell motility. Regarding tumor growth, we showed recently that immunogenic melanoma cells do use MDK to rewire macrophage signaling and promote T cell dysfunction, favoring this way immune evasion and promoting tumor cell growth (Cerezo-Wallis et al. *Nature Medicine*, 2020)[15]. We have added this information to the Introduction and Discussion as it further adds relevance to therapeutic agents that blunt MDK expression.

2.11. In Fig. 4B, the authors demonstrate VEGFR3 mRNA levels correlated to HLEC tube formation. In the representative image in Fig. 4C, it appears as though BO-110 with IgG control actually disrupts tube formation, as the cells in the BO-110 micrograph appear to be growing as a monolayer rather than tubes (also seen in the micrographs in Fig. 2C and EV Fig. 3A), however, it actually appears there is a greater number of LECs in the field of view, which suggested increased LEC proliferation in BO-110-treated groups, which is at odds with reduced VEGFR3 mRNA. It would be useful to differentiate LEC proliferation from LEC migration/tube formation, and to demonstrate whether downstream VEGFR3 signaling is actually reduced as a result of BO-110. Does BO-110 alter LEC function, in particular immunomodulation?

The Referee is correct in that this figure was not sufficiently clear. We have repeated the HLEC tube formation to show the inhibitory effect of BO-110 as a time-lapse video (**Appendix Video 1**), with snapshots in **Fig. 2F**. These figs now show similar starting cell numbers and how the monolayer in control cells reorganize while the BO-110 treated counterparts did not. We also included new data in **Fig. 2G** to show that this activity of BO-110 reflects effects on HLEC function rather on cell death (which was not affected in the conditions tested).

Regarding BO-110 and immunomodulation in HLEC, we thank the Referee for prompting these analyses. We have performed RNAseq and indeed show a high degree of overlap in the effect on melanoma cells and HLEC (**Table II; Fig. 4D,** see additional information in **Fig. EV3B,C**). Importantly, both, melanoma cells and HLEC responded to BO-110 activating dsRNA stress signals associated with IFN-related pathways (**Fig. EV3D**). This similarity was striking with respect to various immunomodulatory cytokines and chemokines (**Fig. 3E, D**), including factors with binding sites in the promoters of *MDK* and *VEGFR3/FLT4* (Fig. EV4A).

2.12. Is there altered type 1 interferon levels in metastatic sites? Does this precede Vegfr3 activation and/or de novo lymphangiogenesis?

We appreciate the insightful question of the reviewer. We have now monitored systemic induction of IFN-$\beta$ by BO-110 in two different xenograft models (B16R2L and SK-Mel-147) to test effects in T cell-

immunocompetent and immune-deficient backgrounds, respectively (**Fig. 6H** and **Fig. 6P**). This quantification was performed at different time points, showing indeed that IFN-induction was quite rapid, preceding the reduction of de novo lymphangiogenesis at the tumor site, lymph nodes, spleen, liver and lung (**Fig. 6B-F** and **Fig. 6J-N**) as also mentioned for Referee #1.

2.13. Lucas ED et al. (J Immunol. 2018) demonstrated that type 1 IFN (along with PDL1) coordinate LEC expansion and contraction during an inflammatory immune response, and it would be of interest to understand whether PDL1 is also modulated by BO-110, considering the efficacy of PDL1 blocking antibodies.
Indeed, our new RNAseq data revealed that BO-110 induces PD-L1 (CD274), but not PD1 both in melanoma and LECs (see heatmap of **Fig. 4E**). We mention these results in the Discussion supporting the rationale for future studies of BO-110 with immune checkpoint blockers.

2.14. After administration of BO-110, where do these dsRNA nanoplexes accumulate? Are they present at metastatic sites? Or could effects be due to systemic IFN/MDK modulation?
Regarding dsRNA nanoplex accumulation, this has been extensively analyzed by Bioncotech Therapeutics (now Highlight Therapeutics) for the clinical development of BO-110. Radioactively labeled B0-110 complexes could be detected at different organs 24 h after treatment (see **Fig. R5**, information submitted to EMA and FDA for clinical trials, www.highlighttherapeutics.com/pipeline-2).

Figure for referees removed

**Referee #3**

We appreciate the comments of the Referee in that "the manuscript is of a topic that is of high interest to a broad audience" and provided evidence to address the gobal critique that " a major revision is necessary that required additional assessments of their model".

Major Comments:
3.1. In the first paragraph of the Introduction, the authors state, "Rather unexplored from a therapeutic perspective (in melanoma and other tumor types) is the lymphatic system." This is not true as several groups have looked at blocking lymphangiogenesis, VEGFR3 in melanoma models in particular, to prevent metastasis in mouse models. Many of the references describing this work is missing from the paper. Reliance on reviews should be minimized and primary literature cited.
We apologize if we gave the impression of dismissing previous references. Indeed, the metastasis field in general, and in melanoma in particular, has been highly influenced by studies of lymphangiogenesis in different animal models and in clinical biopsies. Our intention was indeed to emphasize the relevance of this field by indicating that to date, inhibitors of lymphangiogenesis have not yet been approval for clinical

use. The Introduction was rewritten and we included some seminal references regarding VEGFR3 signaling. For limitations of space, we also used recent reviews that cover this topic.

3.2. A better description of the VEGFR3-Luc mouse. Where are the lymphovascular niches developing in the models? What are these lymph nodes? What are the organs? Does this model also have a signal for tumor angiogenesis as VEGFR3 is also expressed and required for tumor angiogenesis?

As indicated to Referee #2, we acknowledge that we had not provided sufficient detail on the extensive previous characterization of our Vegfr3-Luc mice reported in Martinez-Coral et al. *PNAS*, 2012 and Olmeda D et al *Nature*, 2017. Our animal models show a systemic induction of neo-lymphangiogenesis in untreated mice at proximal and distal lymph nodes, lung, liver and spleen, among other sites (see examples in ***Fig. R3***). In the current study, we analyzed how these signals change with treatment, focusing on BO-110. New data was added in **Fig. 6A-H** and **Fig. 6I-P** for Vegfr3-Luc emission, tumor growth and effects on systemic MDK and IFN-β (two different background settings) to strengthen inhibitory effects of this compound in these organs as indicated in points 1.4 and 2.12 above.

Regarding effects on angiogenesis, we showed before that in these mice, the Vegfr3-Luc signal in angiogenic vessels is significantly lower than in lymphatic cells (Martinez-Coral et al. *PNAS*, 2012 and Olmeda et al *Nature*, 2017). We now provide further histological evidence for the effect of BO-110 on lymphatic endothelial cells dual staining for Lyve1 and Prox1 (**Figs. 1D; Fig. EV1C,D**). The differential effect of BO-110 on the lymphatic and angiogenic vessel density is now presented with the corresponding quantifications in the cutaneous lesions, at lungs and lymph nodes (**Fig. 1E; Fig. EV1E**).

3.3. In Figure 1A, it is unclear what the authors are trying to say. It appears that anti-PD-L1 and vemurafenib were anti-lymphangiogenic in their models as there is less luciferase expressed in the long treatment group. What did lymphangiogenesis look like in the organs with signal? Was the presence of tumor cells assessed?

As we indicated for Referee #2, the text describes now better the main objectives of this figure: (i) demonstrate that Vegfr3-Luc emission in these mouse models can be used as a platform to assess drug response in vivo, (ii) show that αPD-L1 and vemurafenib treatments in these animals recapitulate partial responses observed in the clinic and (iii) identify more potent anti-cancer agents (in this case, BO-110).

Fig. 1A has been splitted in two, to better describe comparative effects of αPD-L1, vemurafenib and BO-110 after multiple treatments (now **Fig. 1B**) and the most relevant, just after one single administration of these compounds in xenograft models (**Fig. 2A**). In brief, **Fig. 1B** and the corresponding quantification in Fig. 1C demonstrate αPD-L1 and vemurafenib have a partial and transient effect (thus recapitulating the clinical situation of aggressive melanomas). Instead, the BO-110 was found as more potent and with a longer-term efficacy. **Fig. 2A** in turn, demonstrated that just one single dose of BO-110 was indeed sufficient to blunt tumor-driven neolymphangiogenesis, while αPD-L1 and vemurafenib had virtually no effect.

The data above was generated in autochthonous melanoma models in the inducible *Tyr::CreERT2; Braf*$^{V600E}$*;Pten*$^{flox/flox}$ background. Sustained effects of BO-110 were also found for patient-derived xenografts after multiple treatments (**Fig. 1F**, Fig. 1G) and after a single dosing (**Fig. 2B**). Together, these results prompted all subsequent analyses in this paper, where we focused on this new role of BO-110 on the lymphatic vasculature.

Histological analyses of lymphatic endothelial cells and the quantifications of lymphatic vessel density in organs with signal was performed as indicated in Point 3.2. (**Figs.1D,E; Fig. EV1C-E**). The presence of melanoma cells at different organs was performed in these and other experiments by monitoring mCherry-labeled melanoma cells either by fluorescence (Fig. 1H; **Fig. 2B**; **Fig. 7B**) or histologically **Fig. 5**. Note that as indicated in Point 1.3 and Point 2.1, we have already reported a comprehensive analysis of tumor dissemination and Vegfr3-Luc emission in untreated animals by qPCR-based analyses prior and after surgical excisions of primary tumors (see ***Fig. R3*** above from Olmeda et al. *Nature*, 2017).

3.4. The fact that anti-PD-L1 and vemurafenib were 100% successful at suppressing primary tumor growth is not unexpected as its growth should not be dependent on neo-lymphangiogenesis. Could reduced tumor growth be due to reduced angiogenesis?

We understand that the Referee is asking why vemurafenib and $\alpha$PD-L1 are not completely efficient in treating melanoma tumors in these mice. Certainly, the partial response to these compounds is multifactorial, as abundantly shown in for other systems in the literature. As tumor size was not reduced completely by vemurafenib or with $\alpha$PD-L1, neither was Vegfr3Luc-lymphangiogenesis (**Fig. 1B**). These compounds were used just as a reference, to validate that the *Vegfr3$^{Luc}$-MetAlert* models recapitulate partial antitumoral responses shown in the clinic. The main objective of this current manuscript, was to show that the *Vegfr3$^{Luc}$-MetAlert* can be exploited to find alternative anti-cancer agents. Discovering that BO-110 was so efficient that could blunt tumor-induced neolymphangiogenesis with one single dosing (**Fig. 2A,B**), was novel and unexpected, hence the subsequent mechanistic of the studies in the manuscript. The text was modified to set objectives on BO-110 more clearly. As for angiogenesis, the reduced tumor growth by this compound was not found associated with a selective reduction of the blood vascular density at the cutaneous lesions, the lung or the lymph nodes (Fig. 1E, Fig. EV1B) as mentioned in Point 3.2.

3.5. Figure 1F is not sufficient to show reduced lymphangiogenesis in the lymph node. 1) LYVE1 is not specific to the lymphatic endothelium and is expressed by macrophages and blood endothelial cells. 2) The low magnification of the image makes it impossible to assess cell type and specificity of staining as the images are pixilated when blown up.

We have now include double Lyve1/Prox staining this Figure (tumor sites, now **Fig. 1D**), also presented at a higher resolution. Histological analyses of dual Lyve1/Prox1 are also shown at different magnifications in lung and lymph nodes **Fig. EV1C** and **Fig. EV1D**. Quantifications to show an inhibitory activity of BO-110 on the lymphangiogenic vessel density without significant effects in the angiogenic vasculature are shown in **Fig. 1E** and **Fig. EV1E**.

3.6. The interpretation of the data presented in Figure 2C is not accurate. 1) the Matrigel assay is transient and only assesses HLEC migration and proliferation and does not assess tube formation. Thus, it is not a tubulogenesis assay. 2) It appears that BO induced HLEC proliferation in this assay at the expense of network formation.

We clarified this question also in response Referee #2 (please see Point 2.11). In brief, we now provide a time-lapse video (**Appendix Video 1**), with snapshots in **Fig. 2F** to better show the effect of BO-110 on HLEC tube formation, which was not due to increased HLEC proliferation as there was no change in cell density nor on cell dettachment.

3.7. How was cell viability assessed in Figure 2D? It was not described in the methods and text in figure legend is not sufficient. There are also better more accurate ways to assess cell viability.

In addition to time-lapse microscopy as mentioned above, we have assessed cell viability by flow cytometry analysis of apoptosis by Anexin V and TMRE (Tetramethylrhodamine, Ethyl Ester, Perchlorate) staining at different time points after treatment with BO-110 (now **Fig. 2G**).

3.8. BO effect on LEC proliferation in vitro and in vivo should be assessed. The Matrigel assays throughout suggest BO promotes LEC proliferation. Moreover, the authors show data that BO downregulates VEGFR3 expression. Thus, the loss of LUC in the mouse model may be due to reduced VEGFR3 expression and not due to reduced lymphangiogenesis. In fact, the authors show that there are LYVE1+ vessels that lose VEGFR3 expression after BO treatment.

We now describe these results better. As mentioned above in points 3.6 and 3.7, we have not found BO-110 to promote the proliferation of HLEC, but rather to prevent their ability to form tubes *in vitro* (**Fig. 2F**). These HLECs do not polarize but also do not die (**Fig. 2G**). This is consistent with the in vivo data the Referee indicates (i.e. Lyve1$^+$ collapsed lymphatic endothelial cells what are negative for Vegfr3, Fig. EV1B). Indeed,

the death of lymphatic cells would not be a beneficial effect of BO-110, as this would likely lead to toxicities related to lymphedema, which we do not observe in our treatments *in vivo*.

With respect to VEGFR3, we would like to clarify that in the *MetAlert* mice, the luciferase was engineered as a knock in strategy to be coupled to the endogenous transcription of the *Vegfr3* gene. Our data shows that BO-110 blocks VEGFR3 expression (Fig. EV1B) and luciferase emission in these mouse models (**Fig. 1B,F; Fig. 2A,B**). This was not the case if the luciferase is coupled to an unrelated promoter (Fig. EV2F,G), ruling out off target effects of BO-110 on luciferase expression or emission.

3.9. "compromised sprouting upon BO-110 treatment" is not demonstrated in Figure 2E. Moreover, quantification of lymphatic density would needs to be done to be able to comment on BO's effect on lymphangiogenesis.
Differential structure of BO-110 treated vessels with respect to the controls are shown in **Fig. 1D; Fig. EV1C,D**. The quantification of the effect of this compound in lymphatic and angiogenic blood vasculature is shown as indicated before in point 3.5.

3.10. VEGF C is also expressed by HLECs. Did BO treatment affect VEGF-C transcripts in HLECs? Could increased VEGF-C be the reason for increased HLEC density in the Matrigel assay Figure 2C?
We had previously shown that BO-110 induces VEGFC in two melanoma cell lines (Fig. 2H), and yet, lymphangiogenesis is blocked (we linked this effect instead to the inhibitory effect of this compound on the pro-lymphangiogenic roles of MDK). The effects of BO-110 in matrigel tube formation assay have been described in Point 2.11 and 3.6 (see **Fig. 2F, G**), and shown by live imaging in **Video 1**.

3.11. What is presented in Figure 3F? The figure legend states it is HLECs treated with BO, while the text states that it was melanoma tumor cells?
Sorry for the confusion. The initial Fig. 3F (now Fig. 4C) corresponded to the induction of IFN-related genes in melanoma cells. A comparative time-course analysis of IFN-β induction by melanoma and HLEC is shown in Fig. 5A. The text was corrected accordingly.

3.12. In Figure 5, expression of Cherry should be presented to show that increased lymphangiogenesis correlated with metastatic growth. As currently presented, the authors cannot comment on growth of metastasis in this model.

The Fig. 5 (now Fig. 7) has been reorganized as indicated by the Referee to show recovery of Vegfr3-Luc/lymphangiogenesis (Fig. 7A) with mCherry emission in parallel (**Fig. 7B**), to show metastatic regrowth at different sites. We agree that in this manner the data are more clear.

Minor Comments:

1) The manuscript (abstract) is hard to follow as it uses a large number of "jargon words" making more a collection of key words rather than presenting an easy to follow story.
2) A description and definition of lymphovascular niche should be provided as it is the basis of their entire study.

We have revised the text to avoid jargon, and provide references from groups of K Alitalo, M Detmar, D Lynden and others, and described the concept of lymphovascular niche (basically, a tumor-activated niche whereby a crosstalk is stablished between tumor cells and lymphatic cells that ultimately create a permissive environment –usually immune tolerant- to favor metastatic growth).

3) Description of metastasis is missing when describing VEGFR3-Luc mouse. Where are the lymphovascular niches developing in the models? What are these lymph nodes? What are the organs?

As indicated above, these questions were extensively addressed in our previous publications in *PNAS*[4] and *Nature*[1], with whole-body imaging of systemic activation of neolymphangiogenesis and proximal and distal organs, as well as in lung, liver, spleen and multiple visceral sites). This reported information is described in more detail now in the Introduction (although for the constraints of the word limitations indicated by the publisher). Note that here we provide now an extensive kinetic characterization of the effect of BO-110 in the tumor and different organs (in immunocompetent and immune-deficient MetAlert strains) in **Fig. 6A-P** (16 panels), including effects of MDK and IFN in liquid biopsies (see Points 1.4, 1.7,2.12).

4) The authors state the t the MetAlert model could be used to identify compounds to block tumor growth and lymphangiogenesis. However, they do not use the model as a drug screening method, but as a preclinical model to test specific drugs. Its use as a screening tool would be time consuming and expensive.
We would like to emphasize that showing that the *MetAlert* can be used as a system for preclinical analyses of specific drugs, and demonstrating that these animals can reveal new modes of action of compounds on the lymphatic vasculature is indeed a solid starting point for subsequent large-scale screening. Here are just some advantages of these animals, that have been appreciated by experts in the literature[5,6,7]:

1. The ability to use non-invasive imaging techniques (i.e without having to sacrifice the mice) for spatiotemporal studies of drug response. This reduces the number of animals needed for analyses and consequently costs.

2. Spatio-temporal studies of drug response at different anatomical sites (i.e. to define differential organ-dependent efficacy), again, monitored in living animals.

3. Visualization of pre-metastatic niches before surgical procedures, and metastatic relapse afterwards, from autochtonous tumors (the imaging of these early events is key in the context of main unmet needs in the cancer field related to adjuvant and neoadjuvant therapies).

5) It is unclear why the authors chose PAMP (BO-110). Was there the rationale besides it.
The rationale was that we had demonstrated that BO-110 is a potent antitumoral agent before (Tormo et al. *Cancer Cell* 2009)[10]. However, we had no means to define its effect at the whole-body level *in vivo*, specifically at premetastatic niches. Moreover, PAMPs such as the dsRNA nanoplexes BO-110, are being actively pursued in the clinic[8,16,17], although with no consensus biomarkers to stratify patients, or with indicators to follow early responses that could help clinicians decide on dosing and combination regimens. Therefore, we considered that investigating new mechanisms of action of BO-110 that could involve the lymphatic vasculature was an attractive hypothesis to pursue.

Finding that BO-110 has now a dual effect on tumor cells and on the lymphatic vasculature, by blocking MDK and VEGFR3 simultaneously, separates this compound from other agents and opens new avenues of research. Furthermore, our transcriptomic findings where we identified rather overlapping (although not equivalent) effects on IFN-driven signaling in both cell types, further support combination therapies with immune checkpoint blockers where antitumoral effects should be considered not only at the level of tumor and immune cells, but also the lymphatic vasculature. These concepts are not put forward in the Discussion as mentioned above for Referee #1 and #2.

References


1 Olmeda, D. *et al.* Whole-body imaging of lymphovascular niches identifies pre-metastatic roles of midkine. *Nature* **546**, 676-680, doi:10.1038/nature22977 (2017).
2 Garcia-Fernandez, M. *et al.* Metastatic risk and resistance to BRAF inhibitors in melanoma defined by selective allelic loss of ATG5. *Autophagy* **12**, 1776-1790, doi:10.1080/15548627.2016.1199301 (2016).
3 Han, D. *et al.* Current management of melanoma patients with nodal metastases. *Clin Exp Metastasis*, doi:10.1007/s10585-021-10099-7 (2021).

4 Martinez-Corral, I. *et al.* In vivo imaging of lymphatic vessels in development, wound healing, inflammation, and tumor metastasis. *Proc Natl Acad Sci U S A* **109**, 6223-6228, doi:1115542109 [pii] 10.1073/pnas.1115542109 (2012).

5 Hoshino, A. & Lyden, D. Metastasis: Lymphatic detours for cancer. *Nature* **546**, 609-610, doi:546609a [pii]10.1038/546609a (2017).

6 Karaman, S. & Alitalo, K. Midkine and Melanoma Metastasis: A Malevolent Mix. *Dev Cell* **42**, 205-207, doi:10.1016/j.devcel.2017.07.015 (2017).

7 Perez-Guijarro, E. & Merlino, G. Lymphangiogenesis: From passive disseminator to dynamic metastatic enabler. *Pigment Cell Melanoma Res* **30**, 509-510, doi:10.1111/pcmr.12621 (2017).

8 Aznar, M. A. *et al.* Immunotherapeutic effects of intratumoral nanoplexed poly I:C. *J Immunother Cancer* **7**, 116, doi:10.1186/s40425-019-0568-2 (2019).

9 Marquez-Rodas, I. *et al.* Intratumoral nanoplexed poly I:C BO-112 in combination with systemic anti-PD-1 for patients with anti-PD-1-refractory tumors. *Sci Transl Med* **12**, doi:10.1126/scitranslmed.abb0391 (2020).

10 Tormo, D. *et al.* Targeted activation of innate immunity for therapeutic induction of autophagy and apoptosis in melanoma cells. *Cancer Cell* **16**, 103-114, doi:10.1016/j.ccr.2009.07.004 (2009).

11 Salazar, A. M., Erlich, R. B., Mark, A., Bhardwaj, N. & Herberman, R. B. Therapeutic In-Situ Autovaccination against Solid Cancers with Intratumoral Poly-ICLC: Case report, Hypothesis, and Clinical Trial. *Cancer Immunol Res*, doi:2326-6066.CIR-14-0024 [pii]10.1158/2326-6066.CIR-14-0024 (2014).

12 Hur, S. Double-Stranded RNA Sensors and Modulators in Innate Immunity. *Annu Rev Immunol* **37**, 349-375, doi:10.1146/annurev-immunol-042718-041356 (2019).

13 Ming Lim, C., Stephenson, R., Salazar, A. M. & Ferris, R. L. TLR3 agonists improve the immunostimulatory potential of cetuximab against EGFR(+) head and neck cancer cells. *Oncoimmunology* **2**, e24677, doi:10.4161/onci.24677 (2013).

14 Rapoport, A. P. *et al.* Combination immunotherapy after ASCT for multiple myeloma using MAGE-A3/Poly-ICLC immunizations followed by adoptive transfer of vaccine-primed and costimulated autologous T cells. *Clin Cancer Res* **20**, 1355-1365, doi:1078-0432.CCR-13-2817 [pii]10.1158/1078-0432.CCR-13-2817 (2014).

15 Cerezo-Wallis, D. *et al.* Midkine rewires the melanoma microenvironment toward a tolerogenic and immune-resistant state. *Nat Med* **26**, 1865-1877, doi:10.1038/s41591-020-1073-3 (2020).

16 Hervas-Stubbs, S. *et al.* Direct effects of type I interferons on cells of the immune system. *Clin Cancer Res* **17**, 2619-2627, doi:1078-0432.CCR-10-1114 [pii]10.1158/1078-0432.CCR-10-1114 (2011).

17 Akira, S. & Takeda, K. Toll-like receptor signalling. *Nat Rev Immunol* **4**, 499-511, doi:10.1038/nri1391 [pii] (2004).

6th Oct 2021

Dear Dr Soengas,

Thank you for the submission of your revised manuscript to EMBO Molecular Medicine. We have received the enclosed reports from referees #1 and #2, and as you will see, they are now supportive of publication. I am therefore pleased to inform you that we will be able to accept your manuscript once the following minor editorial points will be addressed:

1/ Please address the minor remaining comments from the referees.

2/ Main manuscript text:
- Please address the queries from our data editors in track changes mode in the main manuscript file labelled 'Data edited MS file'. Please use this file for any further modification and only keep in track changes mode the new modifications.
- We can accommodate a maximum of 5 keywords, please adjust accordingly.
- Please remove the abbreviation list and instead define abbreviations the first time they appear in the text.
- Please remove the coloured text, and only keep in track changes any new modification.
- Material and methods:
o Mice: please make sure that the gender of the mice is indicated for all experiments. Please also indicate the housing and husbandry conditions (food and day/light cycle).
o When you refer to previously published methods, make sure that the information is accessible to the public and/or provide enough information in the manuscript to ensure reproducibility of the experiments.
o Cells: please indicate the origin of the cells.
- Data Availability section: Please note that the datasets must be publicly available before online publication of the manuscript.

3/ Figures:
- Please provide a legend for your movie that should be zipped with the file.
- Please upload tables 1 and 2 as individual files and rename them Dataset EV1 and Dataset EV2 (the reference to these tables should also be updated in the manuscript text). Both need titles and legends added to the files in a separate tab.
- Figure 7: Most mice (but not all) in panels A and B seem identical. Please clarify.

4/ Checklist:
- in section F18: indicate there that you provided the Data availability section.
- in section F19: provide the url and accession numbers for the datasets generated in this study.

5/ Please note that all corresponding authors are required to supply an ORCID ID for their name upon submission of a revised manuscript. An ORCID identifier is missing for M. Soengas.

6/ Thank you for providing Source Data, please upload them as one file per figure.

7/ Thank you for providing a nice synopsis image. Please remove it from the manuscript and upload it separately as a tiff/png file 550px wide x 300-600px high.
Please also upload the synopsis text separately (and remove it from the main manuscript file).

8/ As part of the EMBO Publications transparent editorial process initiative (see our Editorial at http://embomolmed.embopress.org/content/2/9/329), EMBO Molecular Medicine will publish online a Review Process File (RPF) to accompany accepted manuscripts.
This file will be published in conjunction with your paper and will include the anonymous referee reports, your point-by-point response and all pertinent correspondence relating to the manuscript. Let us know whether you agree with the publication of the RPF and as here, IF YOU WANT TO REMOVE OR NOT any figures from it prior to publication.
Please note that the Authors checklist will be published at the end of the RPF.

I look forward to receiving your revised manuscript.

Yours sincerely,

Lise Roth

Lise Roth, PhD
Editor

To submit your manuscript, please follow this link:

Link Not Available

The system will prompt you to fill in your funding and payment information. This will allow Wiley to send you a quote for the article processing charge (APC) in case of acceptance. This quote takes into account any reduction or fee waivers that you may be eligible for. Authors do not need to pay any fees before their manuscript is accepted and transferred to our publisher.

***** Reviewer's comments *****

Referee #1 (Comments on Novelty/Model System for Author):

The study by Olmeda et al was well conducted. The authors made use of their previously generated "MetAlert" mouse model. Hence, technical quality was medium. However, this model was used as a tool to study effects of BO-110, a dsRNA nanoplex, with strong anti-tumoral effects. As stated by the authors transfer of these findings into clinical studies would be intresting especially for patients at risk for metastasis such as stage III melanoma patients.

Referee #1 (Remarks for Author):

Thank you for this thorough revision. Congratulations, the manuscript gained quality and clarity by the added experiments and remarks!
Previously raised concerns were adequately adressed. Therefore, I now recommend this manuscript for publication after adressing the minor comments below. I believe that these can be resolved during final formatting without the need for a second, minor revision.

Minor comments:

- p. 8: there is a quite extensive paragraph about dsRNA nanoplexes, esp. BO-110, which in my opinion should be put into the introduction (e.g. at the end of it) instead of the results section.
- co-stainings of Prox1 and Lyve1 were included as demanded last year. Unfortunately, the quality of the pictures (Fig. 5E, EV Fig. 1 C,D) is quite poor and contrast very low. It is hard to distinguish light blue-colored "Lyve1" from violett-colored "Prox1". This should be improved prior to publication by enhancing the contrast of the pictures. For the future I recommend using immunofluorescence stainings instead of dual-immunohistochemistry.
- page 9: please correct the typo "we sought out to rule out".
- page 10, first paragraph: To my mind the explanation of gene nomenclature should be clear to the audience of scientific manuscripts. This note is rather distracting and should be deleted.
- page 16, first sentence in discussion: please correct the typo ""MetAlert"" into "MetAlert"

Thank you very much.

Referee #2 (Comments on Novelty/Model System for Author):

The revised manuscript by Olmeda et al. significantly expands upon the initial submission to provide a more detailed and comprehensive view of the efficacy of their dsRNA polyplex (synthetic (poly)inosinic:polycytidylic acid; BO-110) in reducing early neolymphangiogenesis in murine melanoma models. This is facilitated using transgenic MetAlert mice, which provide a novel tool to screen for neolymphangiogenesis. The authors have adequately addressed all the concerns raised in the initial review and the manuscript is suitable for publication.

Referee #2 (Remarks for Author):

MINOR COMMENT:
With respects to response 2.7, the authors provide an interesting new result that shows that BO-110 greatly increases inflammatory gene signatures in both melanoma cells (SK-Mel-147) and HLECs. While the authors discuss the upregulated inflammatory signatures, of note, metabolic gene pathways such as fatty acid metabolism, glycolysis and oxidative phosphorylation are reduced in SK-Mel-147 and HLECs. The role of metabolic pathways in melanoma (and other cancers) has recently garnered much attention (Bergers G & Fendt SM. Nat Rev Cancer. 2021) and it has previously been demonstrated that metabolic pathways can regulate lymphangiogenesis (Wong BW et al. Nature. 2017; Yu P et al. Nature. 2017; Ma W et al. Sci

Adv. 2021). It would be of benefit to briefly discuss these findings, as the relationship between MDK/VEGFR3 and cellular metabolism would be interesting areas for future investigation.

The authors performed the requested changes.

******* Reviewer's comments *******

Referee #1 (Remarks for Author):

Minor comments:
- p. 8: there is a quite extensive paragraph about dsRNA nanoplexes, esp. BO-110,

which in my opinion should be put into the introduction (e.g. at the end of it) instead of the results section.
We consider the paragraph is in the appropriate section (Results) as BO-110 was identified as a new anti-lymphangiogenic factor once testing the MetAlert mice for pharmacological analyses, not before these studies were initiated.

- co-stainings of Prox1 and Lyve1 were included as demanded last year. Unfortunately, the quality of the pictures (Fig. 5E, EV Fig. 1 C,D) is quite poor and contrast very low. It is hard to distinguish light blue-colored "Lyve1" from violett-colored "Prox1". This should be improved prior to publication by enhancing the contrast of the pictures. For the future I recommend using immunofluorescence stainings instead of dual-immunohistochemistry.
The contrast has been enhanced, and higher magnification images were included. We have also made available the original high definition images as source data.

- page 9: please correct the typo "we sought out to rule out".
The typo has been corrected.

- page 10, first paragraph: To my mind the explanation of gene nomenclature should be clear to the audience of scientific manuscripts. This note is rather distracting and should be deleted.
The text has been corrected

- page 16, first sentence in discussion: please correct the typo ""MetAlert"" into "MetAlert"
The typo has been corrected

Referee #2 (Remarks for Author):

MINOR COMMENT:
With respects to response 2.7, the authors provide an interesting new result that shows that BO-110 greatly increases inflammatory gene signatures in both melanoma cells (SK-Mel-147) and HLECs. While the authors discuss the upregulated inflammatory signatures, of note, metabolic gene pathways such as fatty acid metabolism, glycolysis and oxidative phosphorylation are reduced in SK-Mel-147 and HLECs. The role of metabolic pathways in melanoma (and other cancers) has recently garnered much attention (Bergers G & Fendt SM. Nat Rev Cancer. 2021) and it has previously been demonstrated that metabolic pathways can regulate lymphangiogenesis (Wong BW et al. Nature. 2017; Yu P et al. Nature. 2017; Ma W et al. Sci Adv. 2021). It would be of benefit to briefly discuss these findings, as the relationship between MDK/VEGFR3 and cellular metabolism would be interesting areas for future investigation.
We appreciate the suggestion of the reviewer and we agree on the relevance of the findings on metabolic changes that may be induced by BO-110. However, without functional analyses we feel that a thorough discussion would be too speculative at this point and would distract from the main focus of the article (effects on lymphangiogenesis and immune modulation).

We hope this manuscript is now ready for publication.

15th Oct 2021

Dear Dr. Soengas,

Thank you for sending the eTOC and synopsis. We are pleased to inform you that your manuscript is now accepted for publication and is being sent to our publisher to be included in the next available issue of EMBO Molecular Medicine!

Congratulations on your interesting work,

With my best wishes,

Lise Roth

Lise Roth, Ph.D
Scientific Editor
EMBO Molecular Medicine

Follow us on Twitter @EmboMolMed
Sign up for eTOCs at embopress.org/alertsfeeds

EMBO PRESS

YOU MUST COMPLETE ALL CELLS WITH A PINK BACKGROUND

PLEASE NOTE THAT THIS CHECKLIST WILL BE PUBLISHED ALONGSIDE YOUR PAPER

Corresponding Author Name: Marisol Soengas
Journal Submitted to: EMBO Molecular Medicine
Manuscript Number: EMM-2020-12924

### Reporting Checklist For Life Sciences Articles (Rev. June 2017)

This checklist is used to ensure good reporting standards and to improve the reproducibility of published results. These guidelines are consistent with the Principles and Guidelines for Reporting Preclinical Research issued by the NIH in 2014. Please follow the journal's authorship guidelines in preparing your manuscript.

### A- Figures
### 1. Data
### The data shown in figures should satisfy the following conditions:

- the data were obtained and processed according to the field's best practice and are presented to reflect the results of the experiments in an accurate and unbiased manner.
- figure panels include only data points, measurements or observations that can be compared to each other in a scientifically meaningful way.
- graphs include clearly labeled error bars for independent experiments and sample sizes. Unless justified, error bars should not be shown for technical replicates.
- if n< 5, the individual data points from each experiment should be plotted and any statistical test employed should be justified
- Source Data should be included to report the data underlying graphs. Please follow the guidelines set out in the author ship guidelines on Data Presentation.

### 2. Captions

### Each figure caption should contain the following information, for each panel where they are relevant:

- a specification of the experimental system investigated (eg cell line, species name).
- the assay(s) and method(s) used to carry out the reported observations and measurements
- an explicit mention of the biological and chemical entity(ies) that are being measured.
- an explicit mention of the biological and chemical entity(ies) that are altered/varied/perturbed in a controlled manner.

- the exact sample size (n) for each experimental group/condition, given as a number, not a range;
- a description of the sample collection allowing the reader to understand whether the samples represent technical or biological replicates (including how many animals, litters, cultures, etc.).
- a statement of how many times the experiment shown was independently replicated in the laboratory.
- definitions of statistical methods and measures:
  - common tests, such as t-test (please specify whether paired vs. unpaired), simple χ2 tests, Wilcoxon and Mann-Whitney tests, can be unambiguously identified by name only, but more complex techniques should be described in the methods section;
  - are tests one-sided or two-sided?
  - are there adjustments for multiple comparisons?
  - exact statistical test results, e.g., P values = x but not P values < x;
  - definition of 'center values' as median or average;
  - definition of error bars as s.d. or s.e.m.

Any descriptions too long for the figure legend should be included in the methods section and/or with the source data.

**In the pink boxes below, please ensure that the answers to the following questions are reported in the manuscript itself. Every question should be answered. If the question is not relevant to your research, please write NA (non applicable). We encourage you to include a specific subsection in the methods section for statistics, reagents, animal models and human subjects.**

### USEFUL LINKS FOR COMPLETING THIS FORM

http://www.antibodypedia.com
http://1degreebio.org
http://www.equator-network.org/reporting-guidelines/improving-bioscience-research-repor
http://grants.nih.gov/grants/olaw/olaw.htm
http://www.mrc.ac.uk/Ourresearch/Ethicsresearchguidance/Useofanimals/index.htm
http://ClinicalTrials.gov
http://www.consort-statement.org
http://www.consort-statement.org/checklists/view/32-consort/66-title
http://www.equator-network.org/reporting-guidelines/reporting-recommendations-for-tumo
http://datadryad.org
http://figshare.com
http://www.ncbi.nlm.nih.gov/gap
http://www.ebi.ac.uk/ega
http://biomodels.net/
http://biomodels.net/miriam/
http://jjj.biochem.sun.ac.za
http://oba.od.nih.gov/biosecurity/biosecurity_documents.html
http://www.selectagents.gov/

### B- Statistics and general methods

Please fill out these boxes (Do not worry if you cannot see all your text once you press return)

| | |
|---|---|
| 1.a. How was the sample size chosen to ensure adequate power to detect a pre-specified effect size? | NA |
| 1.b. For animal studies, include a statement about sample size estimate even if no statistical methods were used. | Figure legends and Methods |
| 2. Describe inclusion/exclusion criteria if samples or animals were excluded from the analysis. Were the criteria pre-established? | No exclusion was performed |
| 3. Were any steps taken to minimize the effects of subjective bias when allocating animals/samples to treatment (e.g. randomization procedure)? If yes, please describe. | For each of the animal experiments, mice were randomized into the different groups (treatment arms) before starting treatment. |
| For animal studies, include a statement about randomization even if no randomization was used. | Included in Methods section |
| 4.a. Were any steps taken to minimize the effects of subjective bias during group allocation or/and when assessing results (e.g. blinding of the investigator)? If yes please describe. | For quantifications of imuhostochesmitry slides reserchers where blind to the treatments cohorts |
| 4.b. For animal studies, include a statement about blinding even if no blinding was done | No blinding was performed. |
| 5. For every figure, are statistical tests justified as appropriate? | The appropriate statistical test is indicated in each figure panel and Methods section. |
| Do the data meet the assumptions of the tests (e.g., normal distribution)? Describe any methods used to assess it. | Normal distribution of the data was tested using the Shapiro-Wilk test. All assumptions were met. |
| Is there an estimate of variation within each group of data? | Yes |

| | |
|---|---|
| Is the variance similar between the groups that are being statistically compared? | Yes |

## C- Reagents

| | |
|---|---|
| 6. To show that antibodies were profiled for use in the system under study (assay and species), provide a citation, catalog number and/or clone number, supplementary information or reference to an antibody validation profile. e.g., Antibodypedia (see link list at top right), 1DegreeBio (see link list at top right). | The corresponding statistical tests are indicated in each figure panel.<br>AF734: https://www.antibodypedia.com/gene/3429/FLT4/antibody/2365891/AF743<br>Ab14917: https://www.abcam.com/lyve1-antibody-ab14917.html<br>sc-46701: https://www.antibodypedia.com/gene/4105/MDK/antibody/12445/sc-46701<br>AF2727:<br>https://www.antibodypedia.com/gene/34616/PROX1/antibody/695989/AF2727 |
| 7. Identify the source of cell lines and report if they were recently authenticated (e.g., by STR profiling) and tested for mycoplasma contamination. | Source and identification test for each cell line is indicated in methods. All cell lines were authenticated using The GenePrint 10 System (Promega, MA). |

* for all hyperlinks, please see the table at the top right of the document

## D- Animal Models

| | |
|---|---|
| 8. Report species, strain, gender, age of animals and genetic modification status where applicable. Please detail housing and husbandry conditions and the source of animals. | Mouse breeding, induction of nevi and melanomas in Vegfr3Luc GEMM, and drug treatments in vivo are described in the Methods Section. |
| 9. For experiments involving live vertebrates, include a statement of compliance with ethical regulations and identify the committee(s) approving the experiments. | All experiments with mice were performed in accordance with protocols approved by the Institutional Ethics Committee of the CNIO and the Instituto de Salud Carlos III. |
| 10. We recommend consulting the ARRIVE guidelines (see link list at top right) (PLoS Biol. 8(6), e1000412, 2010) to ensure that other relevant aspects of animal studies are adequately reported. See author guidelines, under 'Reporting Guidelines'. See also: NIH (see link list at top right) and MRC (see link list at top right) recommendations. Please confirm compliance. | ARRIVE guidelines have been considered and applied. |

## E- Human Subjects

| | |
|---|---|
| 11. Identify the committee(s) approving the study protocol. | NA |
| 12. Include a statement confirming that informed consent was obtained from all subjects and that the experiments conformed to the principles set out in the WMA Declaration of Helsinki and the Department of Health and Human Services Belmont Report. | NA |
| 13. For publication of patient photos, include a statement confirming that consent to publish was obtained. | NA |
| 14. Report any restrictions on the availability (and/or on the use) of human data or samples. | NA |
| 15. Report the clinical trial registration number (at ClinicalTrials.gov or equivalent), where applicable. | NA |
| 16. For phase II and III randomized controlled trials, please refer to the CONSORT flow diagram (see link list at top right) and submit the CONSORT checklist (see link list at top right) with your submission. See author guidelines, under 'Reporting Guidelines'. Please confirm you have submitted this list. | NA |
| 17. For tumor marker prognostic studies, we recommend that you follow the REMARK reporting guidelines (see link list at top right). See author guidelines, under 'Reporting Guidelines'. Please confirm you have followed these guidelines. | NA |

## F- Data Accessibility

| | |
|---|---|
| 18: Provide a "Data Availability" section at the end of the Materials & Methods, listing the accession codes for data generated in this study and deposited in a public database (e.g. RNA-Seq data: Gene Expression Omnibus GSE39462, Proteomics data: PRIDE PXD000208 etc.) Please refer to our author guidelines for 'Data Deposition'.<br><br>Data deposition in a public repository is mandatory for:<br>a. Protein, DNA and RNA sequences<br>b. Macromolecular structures<br>c. Crystallographic data for small molecules<br>d. Functional genomics data<br>e. Proteomics and molecular interactions | We provide a data availability section at the end of Material & Methods section |
| 19. Deposition is strongly recommended for any datasets that are central and integral to the study; please consider the journal's data policy. If no structured public repository exists for a given data type, we encourage the provision of datasets in the manuscript as a Supplementary Document (see author guidelines under 'Expanded View' or in unstructured repositories such as Dryad (see link list at top right) or Figshare (see link list at top right). | The RNAseq data is deposited in NCBI's Gene Expression Omnibus database, with accession number GSE180629 (https://www.ncbi.nlm.nih.gov/geo/query/acc.cgi?acc=GSE180629) . The mass spectrometry proteomics data have been deposited to the ProteomeXchange Consortium via the PRIDE (http://www.proteomexchange.org/) partner repository with the dataset identifier PXD007000. |
| 20. Access to human clinical and genomic datasets should be provided with as few restrictions as possible while respecting ethical obligations to the patients and relevant medical and legal issues. If practically possible and compatible with the individual consent agreement used in the study, such data should be deposited in one of the major public access-controlled repositories such as dbGAP (see link list at top right) or EGA (see link list at top right). | NA |
| 21. Computational models that are central and integral to a study should be shared without restrictions and provided in a machine-readable form. The relevant accession numbers or links should be provided. When possible, standardized format (SBML, CellML) should be used instead of scripts (e.g. MATLAB). Authors are strongly encouraged to follow the MIRIAM guidelines (see link list at top right) and deposit their model in a public database such as Biomodels (see link list at top right) or JWS Online (see link list at top right). If computer source code is provided with the paper, it should be deposited in a public repository or included in supplementary information. | NA |

## G- Dual use research of concern

| | |
|---|---|
| 22. Could your study fall under dual use research restrictions? Please check biosecurity documents (see link list at top right) and list of select agents and toxins (APHIS/CDC) (see link list at top right). According to our biosecurity guidelines, provide a statement only if it could. | NA |
